# Effects of exercise modalities and doses on brachial artery flow-mediated dilation in adults with overweight or obesity: a Bayesian dose-response network meta-analysis

**DOI:** 10.3389/fcvm.2026.1916772

**Published:** 2026-07-23

**Authors:** Dongze Li, Wen Li, Tianfu Yu, Xiaoxiao Xu

**Affiliations:** 1 Department of Health Economics, General Hospital of Eastern Theater Command, Nanjing, China; 2 School of Sports Science and Engineering, East China University of Science and Technology, Shanghai, China; 3 School of Physical Education, North East Normal University, Changchun, China; 4 Department of Public Basic Courses, Sichuan College of Traditional Chinese Medicine, Mianyang, China

**Keywords:** Bayesian dose-response network meta-analysis, brachial artery flow-mediated dilation, endothelial function, exercise prescription, obesity, overweight

## Abstract

**Background:**

Endothelial dysfunction is an early vascular abnormality in adults with overweight or obesity. Although exercise can improve vascular function, the most favorable exercise modality and weekly dose for improving brachial artery flow-mediated dilation (brachial FMD) remain unclear. This study compared exercise modalities and weekly dose-response relationships for brachial FMD.

**Methods:**

We conducted a systematic review and Bayesian dose-response network meta-analysis of parallel-group randomized controlled trials. Eligible trials enrolled adults with overweight or obesity, compared exercise lasting at least 4 weeks with non-exercise control or another modality, and reported pre- and post-intervention brachial FMD. Exercise dose was expressed as MET-min/week. Effects were expressed as mean differences (MDs) in change in brachial FMD. Eight dose-response functions were compared using MBNMAdose in R, and certainty of evidence was assessed with CINeMA.

**Results:**

Forty-seven trials (2,211 participants) were included. The quadratic random-effects model provided the best fit (DIC = 322.6). Overall, the largest model-estimated improvement occurred at 1,500 MET-min/week (MD: 4.72 percentage points, 95% CrI: 3.43, 5.91). Among modalities, combined aerobic and resistance training (AE-RT) showed the largest estimated improvement at 1,500 MET-min/week (MD: 5.77, 95% CrI: 4.04, 7.52). AE-RT at 1,200–1,600 MET-min/week was most consistently ranked among the highest predicted responses, with 1,400 MET-min/week ranked first. Subgroup patterns were broadly consistent across baseline brachial FMD, BMI, health status, and duration. Sensitivity analysis excluding high-risk-of-bias trials remained consistent (1,500 MET-min/week; MD: 4.84, 95% CrI: 3.53, 6.05). Meta-regression did not identify statistically clear effect modification by age, baseline brachial FMD, intervention length, or baseline BMI. Across all 13 network comparisons, the certainty of evidence was low or very low. The mind-body exercise optimum lay below the observed range, an unreliable extrapolation.

**Conclusion:**

In adults with overweight or obesity, exercise showed dose-dependent improvement in brachial FMD, with AE-RT around 1,500 MET-min/week showing the most favorable model-estimated profile. These estimates should be interpreted as provisional, model-based prescription anchors rather than ready-to-apply clinical thresholds, and should be individualized according to clinical status, exercise tolerance, and safety considerations. The low-to-very-low certainty of evidence warrants cautious interpretation and confirmation in higher-quality head-to-head randomized trials using standardized brachial FMD protocols.

**Systematic Review Registration:**

https://www.crd.york.ac.uk/PROSPERO/view/CRD420261402756, PROSPERO CRD420261402756.

## 1 Introduction

In adults who are overweight or obese, vascular dysfunction tends to emerge early, often preceding overt cardiometabolic disease. Excess adiposity drives insulin resistance and chronic low-grade inflammation, raises oxidative stress, and disturbs endothelial signaling; these changes can lower nitric oxide bioavailability and undermine vascular homeostasis (1–3). Dysregulated adipokine signaling and related metabolic inflammatory mediators may further contribute to this endothelial impairment, particularly in obesity and type 2 diabetes (4–6). Because the global burden of overweight and obesity remains high (7), identifying interventions that improve vascular function in adults with overweight or obesity is clinically relevant for cardiometabolic prevention.

Brachial artery flow-mediated dilation (brachial FMD) is a non-invasive ultrasound index of conduit artery endothelial function, capturing how the brachial artery dilates once a brief period of ischemia is released. Because it is straightforward to obtain, it has become a common vascular endpoint in exercise and cardiometabolic trials (8, 9). Reduced brachial FMD tracks with later cardiovascular events, and pooled analyses indicate that it adds prognostic information on top of conventional risk factors (10–12). Taken together, these properties make brachial FMD a useful intermediate target for judging whether a given strategy is likely to benefit vascular health in adults with overweight or obesity.

Exercise is a modifiable intervention that can improve endothelial function through repeated hemodynamic and metabolic stimuli. Regular exercise increases episodic shear stress, may enhance nitric oxide-mediated vasodilatory signaling, and can induce vascular adaptation beyond changes in conventional risk factors (13–15). In adults with overweight or obesity, trials have tested a range of approaches: moderate- or high-intensity continuous aerobic training, resistance training, high-intensity interval training, combined aerobic and resistance training, and mind-body exercise. Yet modality and weekly dose differ widely from one trial to the next, and lumping all of these together as a single “exercise” category risks obscuring how the vascular response actually varies.

Most existing systematic reviews and meta-analyses have asked whether exercise improves endothelial function (16–18), and far fewer have looked at how the brachial FMD response shifts across exercise modalities and weekly doses in adults with overweight or obesity. That gap matters for prescription. Adding more weekly volume does not necessarily buy proportionally more vascular benefit, and the dose linked to the largest response may not be the same from one modality to another. A conventional pairwise meta-analysis cannot resolve this, since it has no way to compare several modalities at once while also treating dose as a continuous exposure across the network.

Bayesian dose-response network meta-analysis provides a framework for addressing this gap by integrating direct and indirect evidence across exercise modalities. This approach is particularly useful when evidence networks include multiple exercise modalities and heterogeneous intervention protocols, because it explicitly quantifies uncertainty around the estimated dose-response relationships. Compared with conventional pairwise meta-analyses, this framework permits simultaneous ranking of exercise modalities and modeling of how brachial FMD responses change as weekly exercise dose, expressed as MET-min/week, varies across the observed range, which may support more actionable prescription targets than overall effect estimates alone.

Therefore, this systematic review and Bayesian dose-response network meta-analysis aimed to evaluate the effects of different exercise modalities and weekly exercise doses on brachial FMD in adults with overweight or obesity. By jointly comparing modality and dose, we sought to move beyond the question of whether exercise improves brachial FMD and toward dose-informed evidence that may inform more targeted exercise prescription for the management of endothelial function in this population.

## 2 Methods

### 2.1 Protocol and registration

This systematic review and Bayesian dose-response network meta-analysis was pre-registered in PROSPERO (registration number: CRD420261402756) and conducted in accordance with the PRISMA 2020 statement and the PRISMA-NMA extension (19, 20). Methodological procedures were guided by the Cochrane Handbook for Systematic Reviews of Interventions (21).

### 2.2 Search strategy and selection

Electronic databases searched for the literature review included PubMed, Web of Science, Embase, Cochrane CENTRAL, Scopus, SPORTDiscus, and ClinicalTrials.gov, covering the period from database inception to 20 May 2026, with no language or publication-date restriction. The search strategy used relevant keywords and controlled vocabulary terms related to the participants, including overweight, obesity, and common obesity-related cardiometabolic conditions; the intervention, exercise or training; the outcome, brachial FMD or endothelial function; and the study design, randomized controlled trials. These concepts were combined using Boolean operators. Detailed search strategies for each database are provided in Supplementary 1.1–1.7. In addition, snowballing techniques were applied, including backward citation tracking of included studies and relevant reviews and forward citation searching, to identify further eligible studies not indexed in the databases.

Two reviewers independently screened titles and abstracts following the predefined eligibility criteria using Zotero 7.0 for reference management and duplicate removal. Full texts of potentially eligible records were subsequently reviewed by the same two reviewers. Disagreements at any stage were resolved through discussion, with a third reviewer arbitrating when consensus could not be reached.

### 2.3 Eligibility criteria

Eligibility criteria were established using the PICOS format (Population, Intervention, Comparison, Outcomes, Study Design) to minimize clinical bias in study selection (22).

(1) Population: adults with overweight or obesity (BMI ≥25 kg/m^2^), whether or not they also had cardiometabolic comorbidities, for example hypertension, type 2 diabetes, metabolic syndrome, coronary artery disease, heart failure, polycystic ovary syndrome, or other obesity-related metabolic disorders.
(2) Intervention: The intervention was exercise, defined as planned, structured, and repetitive physical activity intended to improve or maintain physical fitness. To allow dose–response coding, an eligible program had to report exercise type, intensity, session duration, weekly frequency, and total length in sufficient detail, and it had to last at least 4 weeks, a threshold used to focus on training-related rather than acute vascular responses and consistent with prior randomized-trial meta-analyses of exercise training and endothelial function (23). Where exercise was combined with diet, medication changes, education, or other non-exercise components, the study qualified only if the comparator group received the same co-intervention, which isolates the independent effect of exercise.
(3) Comparison: eligible comparators were non-exercise control, usual care, attention control, or a different exercise modality in head-to-head trials; an alternative modality or dose could itself serve as the comparator.
(4) Outcome: brachial FMD, reported as a percentage, assessed before and after the intervention. Studies were required to provide sufficient data to calculate change in brachial FMD.
(5) Study design: only parallel-group randomized controlled trials were included. Non-randomized trials, quasi-experimental studies, observational studies, reviews, conference abstracts, letters, dissertations, duplicate reports, and secondary publications without additional eligible data were excluded.

### 2.4 Data extraction and coding

Two reviewers (D.L. and W.L.) independently extracted data using a pre-designed form, capturing study details, including author, publication year, country, and study design; participant characteristics, including age, sex distribution, sample size, health status, BMI, and baseline brachial FMD; intervention characteristics, including exercise modality, intensity, session duration, weekly frequency, and intervention length; comparator characteristics; and outcome data, including pre- and post-intervention brachial FMD means and SDs, adherence, and adverse events. Mean difference and SD for the change from baseline to post-intervention were extracted or calculated for both exercise and comparator groups. When SDs for change scores were not reported, they were estimated from baseline and post-intervention SDs using a prespecified correlation coefficient of 0.5 (22). Missing SDs were derived from confidence intervals, *p* values, or *t* values when available; otherwise, the original authors were contacted where necessary. For dose-response analyses, standard errors were required. SDs were converted to SEs using the formula:

$$\mathrm{SE}=\frac{\mathrm{SD}}{\sqrt{N}}$$

where N represents the sample size. Data accuracy was cross-checked by the reviewers; any discrepancies in the extracted data were resolved by discussion, with a third reviewer consulted when consensus could not be reached, and the third reviewer verified the final dataset.

For the dose-response network meta-analysis, interventions were categorized at three levels. At the first level, interventions were classified as exercise or control. At the second level, exercise interventions were further categorized into six modalities according to the original trial descriptions: aerobic exercise (AE), resistance training (RT), combined aerobic and resistance training (AE-RT), high-intensity interval training (HIIT), moderate-intensity continuous training (MICT), and mind-body exercise (MBE). At the third level, interventions were classified by total weekly exercise dose, calculated as the product of exercise intensity, session duration, and weekly frequency, and expressed as MET-min/week.MET-min/week was used as a common external-dose metric because it allowed interventions with different session durations, weekly frequencies, and nominal intensities to be compared on a shared weekly scale. This metric was not intended to imply that the same MET-min/week value produces an equivalent internal physiological stimulus across exercise modalities. MET values were assigned using the 2024 Adult Compendium of Physical Activities according to the exercise type and intensity reported in the original studies (24); the specific activity codes applied to each modality are provided in Supplementary Tables S1 and S3. For resistance training, which the Compendium represents with a limited set of values, we applied the general resistance (weight) training code, using higher-intensity codes only when studies explicitly described vigorous or circuit protocols. For multi-component interventions, total weekly dose was calculated by summing component-specific MET-min/week values. Control groups were assigned a dose of 0 MET-min/week. The specific allocation process is provided in Supplementary Table S3.

Within this scheme, we reserved the label MICT for interventions that the source reports themselves described as moderate-intensity continuous training, and coded the remaining continuous aerobic programs as AE. Keeping the two apart let us stay faithful to the wording each trial used, and it preserved the HIIT-vs.-MICT contrast that is standard in exercise physiology.

### 2.5 Statistical analysis

The dose-response analysis, which examined the relationship between weekly exercise dose and exercise modality, was conducted using the MBNMAdose package in R (version 4.5.2, https://www.r-project.org).

This approach allows different exercise modalities and doses to be modeled simultaneously while integrating direct and indirect evidence across the treatment network. Because brachial FMD is a continuous outcome measured on the same scale across studies, treatment effects were expressed as mean differences in change in brachial FMD, in percentage points. Positive values indicated greater improvement in brachial FMD.

For the dose-response network meta-analysis, we first assessed whether the analysis met key assumptions for network meta-analysis, including network connectivity, consistency, and transitivity (25–27). Transitivity was assessed by comparing the distribution of potential effect modifiers across treatment groups, including age, BMI, baseline brachial FMD, and female percentage; health status, medication use, disease severity, exercise supervision, and exercise tolerance were considered qualitatively because they were not uniformly reported across trials.The distribution of these modifiers across treatment nodes is summarized in Supplementary Table S15. Several recommended dose-response functions were then compared, including Emax, restricted cubic spline, log-linear, quadratic, exponential, non-parametric monotonic, spline, and fractional polynomial functions. Model fit was evaluated using the deviance information criterion, residual deviance, residual SD, model complexity, and visual agreement with observed data (27). Given the anticipated clinical and methodological heterogeneity across exercise protocols and study populations, a random-effects model was used as the default analysis model to provide more conservative estimates. The quadratic random-effects model had the lowest DIC (322.6) and was selected as the primary model.

In the dose-response analysis, an effect was considered statistically significant when its 95% credible interval did not include zero. Model convergence was assessed using Brooks-Gelman-Rubin diagnostics (28). Under the dose-response network meta-analysis framework, all modality-dose combinations were ranked according to their posterior mean ranks to identify pairings most likely to produce the largest improvement in brachial FMD. Lower posterior mean ranks indicate a higher probability of producing the largest brachial FMD response. For clinical interpretation, model-estimated MET-min/week doses were translated into approximate weekly minutes using the same MET values applied during dose coding.

### 2.6 Additional analyses

Sensitivity analysis excluding studies with a high risk of bias was conducted to assess the robustness of the findings. Potential effect modification was explored using subgroup analyses stratified by baseline brachial FMD (≤6.5% vs. >6.5%), BMI category (25.0–29.9 vs. ≥30 kg/m^2^), health status (apparently healthy vs. cardiometabolic disease), and intervention duration (4–8, 10–12, and >12 weeks). The baseline brachial FMD cutoff was prespecified according to the median baseline value in the eligible dataset; this stratification was used because brachial FMD is a prognostically relevant marker of endothelial function and may influence interpretation of vascular responsiveness (8, 10–12). BMI categories followed standard adult overweight and obesity classifications (7). Health-status subgrouping was used because obesity-related endothelial dysfunction often coexists with hypertension, type 2 diabetes, metabolic syndrome, and cardiovascular disease, which may alter vascular adaptation to exercise (1–3). Intervention-duration categories were selected to distinguish shorter, commonly used 10–12-week, and longer exercise-training periods, consistent with previous exercise and endothelial function reviews (17, 18). The number of contributing trials and data points is reported for each subgroup in Table 1; subgroup estimates informed by fewer than 15 trials were regarded as exploratory and interpreted with particular caution.

**Table 1:** Summary of dose-response network meta-analysis result.

Category	Trials (n)	Data points	Outcome	Modeled dose range (MET-min/week)	Optimal dose (MET-min/week)	Observed dose range (MET-min/week)	Mean difference and 95% CrI (optimal dose)
Overall exercise	47 (48 cohorts)^a^	107	FMD	∼2,300	1,500	150–2,500	4.72%-points (3.43, 5.91)
Baseline FMD ≤6.5%	26	60	FMD	∼1,400	1,400	200–1,450	4.30%-points (1.87, 6.85)
Baseline FMD >6.5%	19	40	FMD	∼2,200	1,400	150–2,500	5.94%-points (3.86, 8.14)
BMI 25.0–29.9 kg/m^2^	29	65	FMD	∼2,300	1,400	150–2,500	4.99%-points (3.71, 6.27)
BMI ≥30 kg/m^2^	14	30	FMD	∼1,000	1,000	250–1,200	3.51%-points (0.53, 6.53)
Apparently healthy	15	32	FMD	∼2,000	1,400	150–2,500	5.49%-points (2.22, 8.84)
Cardiometabolic disease	33	75	FMD	∼1,900	1,800	200–1,900	4.87%-points (1.90, 7.88)
Length (4–8 weeks)	13	30	FMD	∼1,000	1,000	150–1,000	5.10%-points (1.37, 9.02)
Length (10–12 weeks)	24	54	FMD	∼2,200	1,500	150–2,500	3.82%-points (2.16, 5.32)
Length (>12 weeks)	11	23	FMD	∼1,900	1,900	300–1,900	6.33%-points (0.63, 12.16)
Sensitivity analysis	44	98	FMD	∼2,300	1,500	150–2,500	4.84%-points (3.53, 6.05)
AE	18	18	FMD	∼1,400	1,200	400–1,450	4.15%-points (2.53, 5.68)
RT	9	9	FMD	∼900	900	450–900	4.16%-points (2.25, 6.05)
HIIT	18	18	FMD	∼1,000	1,000	150–1,000	3.75%-points (1.93, 5.65)
MICT	11	11	FMD	∼760	760	300–850	1.86%-points (0.04, 3.62)
AE-RT	11	11	FMD	∼2,400	1,500	250–2,500	5.77%-points (4.04, 7.52)
MBE	2	2	FMD	∼140	140	300–500	1.31%-points (0.002, 2.65)

Data are model-based estimates.

a Trial counts reflect independent study cohorts; the two cohorts reported by Alvarez et al. (2024) are counted separately, so the total of 48 cohorts corresponds to 47 published reports. In modality-specific rows, each contributing trial provided one arm of that modality, and shared control arms were counted only in the overall row; therefore, data points equal the number of trials. In the overall and subgroup rows, trials could contribute multiple arms, so data points exceed the number of trials. The model-estimated optimal dose for MBE (140 MET-min/week) fell below its observed dose range (300–500 MET-min/week) and should be regarded as an unreliable extrapolation.

AE, aerobic exercise; AE-RT, combined aerobic and resistance training; RT, resistance training; HIIT, high-intensity interval training; MICT, moderate-intensity continuous training; MBE, mind-body exercise.

Meta-regression analyses were also conducted to examine whether age, baseline brachial FMD, intervention length, and baseline BMI significantly modified the dose-response relationship. Study-level covariates were centered before modeling. Regression coefficients with 95% credible intervals were estimated, with a credible interval crossing zero indicating no statistically clear effect modification. Finally, comparison-adjusted funnel plots, Begg's test, and Egger's test were used to assess publication bias and small-study effects (29–31).

### 2.7 Risk of bias and certainty of evidence

Risk of bias was assessed using the Cochrane Risk of Bias tool 2.0, which evaluates five domains: randomization process, deviations from intended interventions, missing outcome data, outcome measurement, and selection of the reported result (32). Studies were classified as having low risk, some concerns, or high risk of bias. Two reviewers independently conducted the assessment, with disagreements resolved by discussion or adjudication by a third reviewer. Reviewer initials were D.L. and W.L., and the third reviewer was X.X.

The confidence in the results was assessed using the Confidence in Network Meta-Analysis web application. This framework is based on the GRADE approach and is specifically designed for network meta-analysis (33–35). It evaluates each comparison across six domains: within-study bias, reporting bias, indirectness, imprecision, heterogeneity, and incoherence. The overall confidence in the evidence was rated as high, moderate, low, or very low. Because no universally accepted minimum clinically important difference exists for brachial FMD, imprecision judgments considered the width of the credible interval and its relationship to the null value.

## 3 Results

### 3.1 Study selection and characteristics

**Figure 1:**
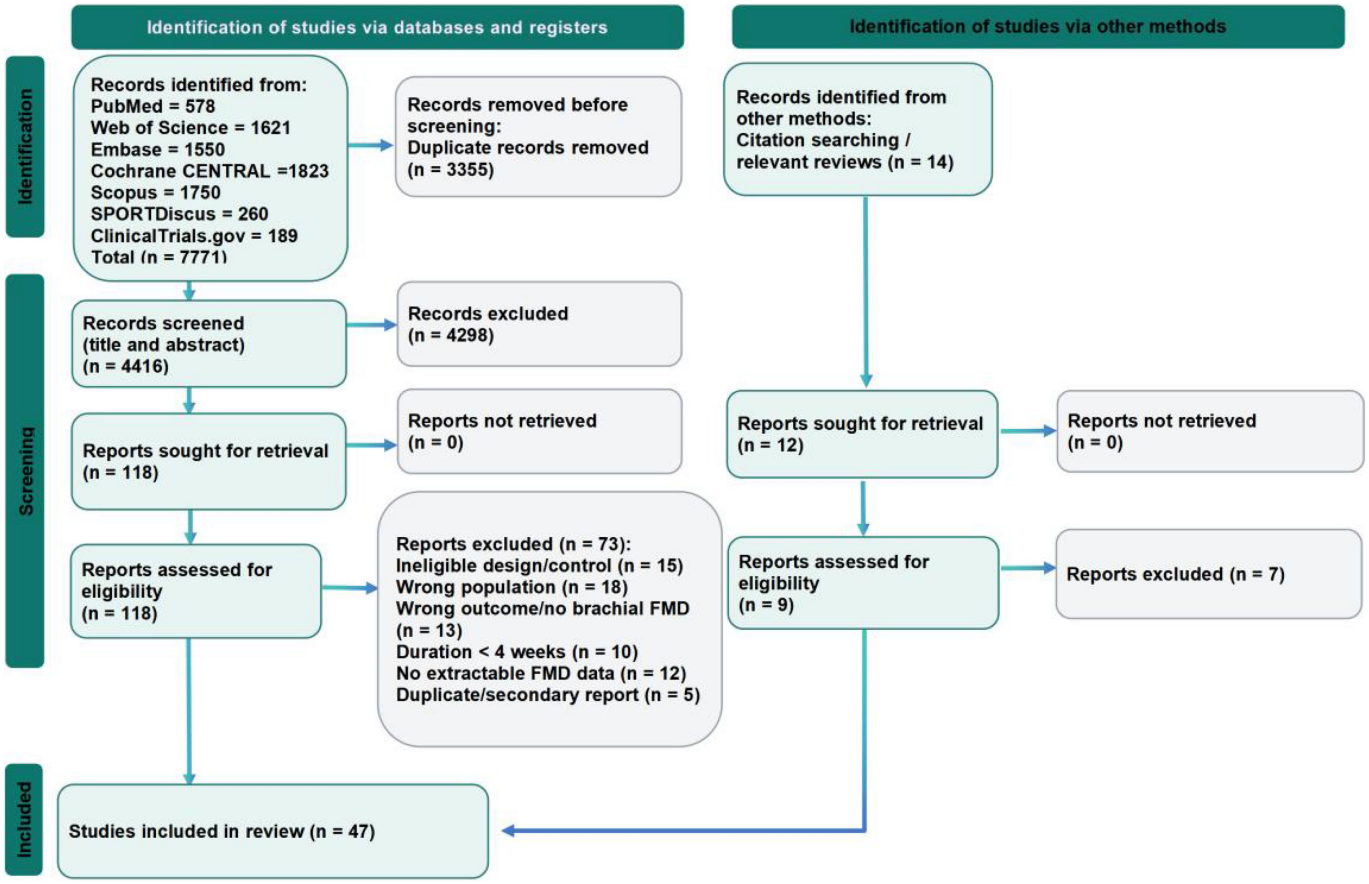
PRISMA flowchart of study selection.

Database searches identified 7,771 records: PubMed (*n* = 578), Web of Science (*n* = 1,621), Embase (*n* = 1,550), Cochrane CENTRAL (*n* = 1,823), Scopus (*n* = 1,750), SPORTDiscus (*n* = 260), and ClinicalTrials.gov (*n* = 189). After removing 3,355 duplicates, 4,416 records were screened by title and abstract, of which 4,298 were excluded. Full texts were assessed for 118 reports, and 73 were excluded: ineligible design or control condition (*n* = 15), wrong population (*n* = 18), wrong outcome or no brachial FMD assessment (*n* = 13), intervention duration shorter than 4 weeks (*n* = 10), no extractable brachial FMD data (*n* = 12), and duplicate or secondary report (*n* = 5). Citation tracking and snowballing identified 14 additional records; 12 remained after initial screening, 9 were assessed in full text, 7 were excluded, and 2 were included. A total of 47 randomized controlled trials were included in this review (Figure 1).

The 47 included trials comprised 2,211 participants. Trial populations included adults with overweight or obesity (BMI ≥25 kg/m^2^), spanning apparently healthy participants with elevated BMI alone and participants with comorbid cardiometabolic conditions such as hypertension, type 2 diabetes, coronary artery disease, heart failure, metabolic syndrome, polycystic ovary syndrome, and obesity-related metabolic disorders. The eligibility definition was based on BMI because this was the most consistently reported adiposity-related measure across the eligible trials. More direct body-composition or fat-distribution indicators, such as body fat percentage, waist circumference, waist-to-hip ratio, or dual-energy x-ray absorptiometry-derived measures, were not consistently available across the included trials. Across study arms, mean age ranged from 20.2 to 72.9 years, reported mean BMI ranged from 25.2 to 38.2 kg/m^2^, and the proportion of female participants ranged from 0% to 100%. Exercise interventions included AE, RT, AE-RT, HIIT, MICT, and MBE.Of the included trials, three combined exercise with a dietary or weight-loss co-intervention; consistent with the eligibility criteria, the co-intervention was applied equally to the exercise and comparator arms, so the exercise contrast was not confounded by diet. Adherence to the prescribed exercise was reported in 26 of the included trials; where reported, it was generally high (median 90%, range 50% to 100%), with most trials reporting at least 80% adherence.Detailed study and intervention characteristics are provided in Supplementary Table S2.

### 3.2 Risk of bias

Overall, 28 trials were judged at low risk of bias, 15 had some concerns, and 4 were judged at high risk of bias. For the randomization process domain, 36 trials were judged low risk, 10 had some concerns, and 1 was high risk. For deviations from intended interventions, 44 trials were low risk and 3 had some concerns. For missing outcome data, 38 trials were low risk, 6 had some concerns, and 3 were high risk. All 47 trials were judged low risk for measurement of the outcome. For selection of the reported result, 42 trials were low risk and 5 had some concerns. Detailed and summary risk-of-bias assessments are shown in Supplementary Figures S1, S2.

### 3.3 Network assumptions and model selection

The dose-level and agent-level networks were connected (Supplementary Figures S3, S4).Consistency assessment using unrelated mean effects models and node-splitting did not suggest important inconsistency between direct and indirect evidence (Supplementary Tables S4, S5). Additional transitivity assessment did not suggest statistically significant imbalance across treatment groups for age *p* = 0.88, BMI *p* = 0.94, baseline brachial FMD *p* = 0.66, or female percentage *p* = 0.57 (Supplementary Table S15 and Figures S19–S22). Among the eight candidate dose-response functions, the quadratic random-effects model provided the best overall fit, with the lowest DIC (322.6), acceptable residual deviance and residual SD, and visual agreement with observed data (Supplementary Table S6 and Figure S5). This model was therefore used for the primary dose-response analyses.

### 3.4 Overall and modality-specific dose-response relationships

**Figure 2:**
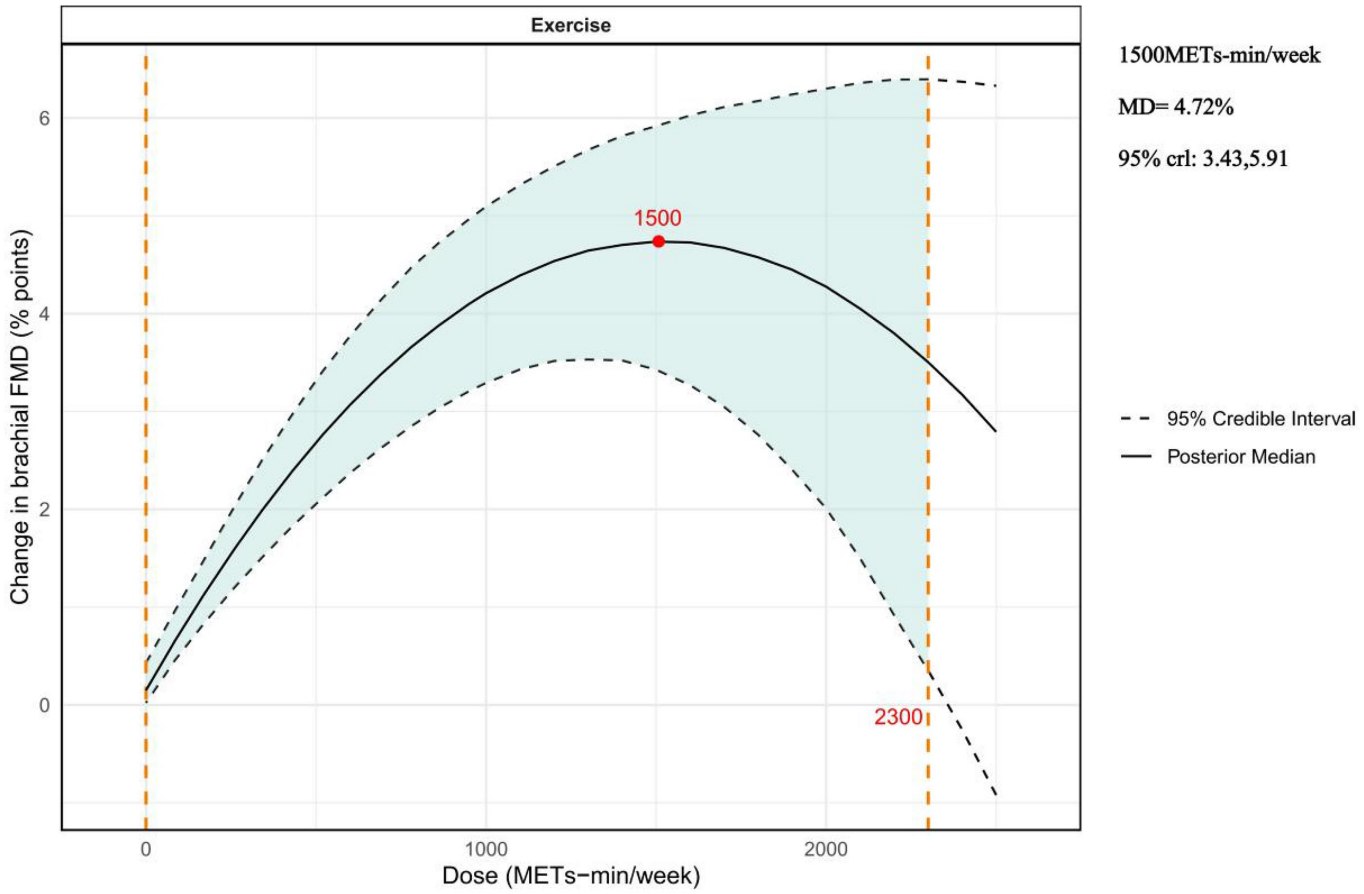
Dose-response relationship between exercise and brachial flow-mediated dilation in adults with overweight or obesity. The orange dashed lines indicate the modeled dose range from ∼2,300 METs-min/week. The solid line represents the posterior median, and the dashed curves represent the 95% credible interval. The light green shaded area shows the uncertainty band around the dose-response curve. The red dot marks the dose associated with the largest estimated improvement in brachial FMD within the modeled range, 1,500 METs-min/week.

**Figure 3:**
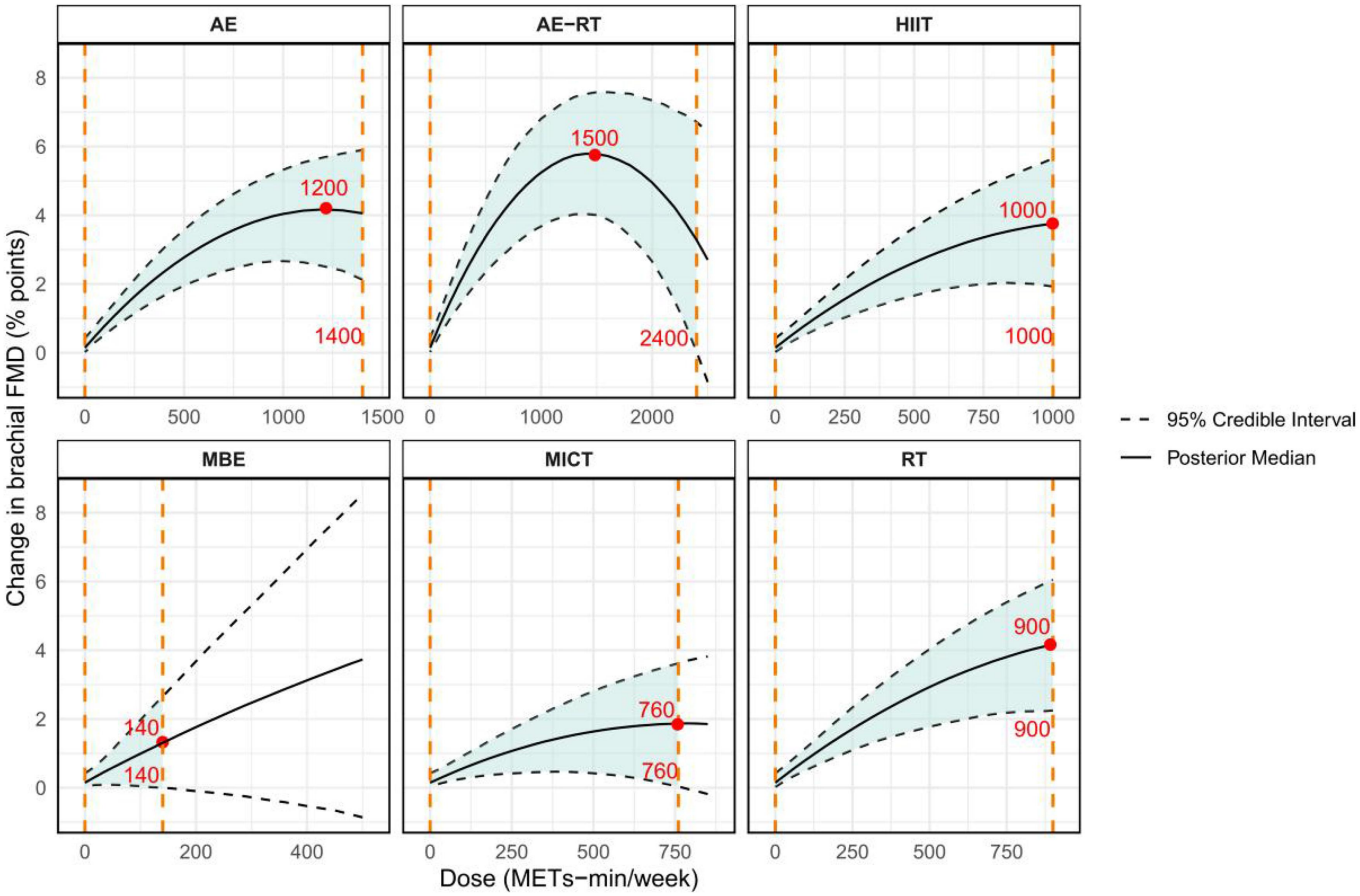
Modality-specific dose-response relationships between exercise and brachial flow-mediated dilation in adults with overweight or obesity. The orange dashed lines indicate the modeled dose range for each exercise modality. The solid lines represent the posterior medians, and the dashed curves represent the 95% credible intervals. The light green shaded areas show the uncertainty bands around the dose-response curves. The red dots mark the doses associated with the largest estimated improvement in brachial FMD within each modeled range. For mind-body exercise, the estimated optimal dose (140 MET-min/week) lies below the observed dose range (300–500 MET-min/week) and represents an unreliable extrapolation that should not be interpreted as an effective dose. AE, aerobic exercise; AE-RT, combined aerobic and resistance training; RT, resistance training; HIIT, high-intensity interval training; MICT, moderate-intensity continuous training; MBE, mind-body exercise; brachial FMD, brachial artery flow-mediated dilation.

Across all exercise modalities, the dose associated with the largest model-estimated improvement in brachial FMD was 1,500 MET-min/week (MD: 4.72 percentage points, 95% CrI: 3.43, 5.91), with the analysis covering weekly doses up to approximately 2,300 MET-min/week based on the observed dose distribution (Figure 2). Modality-specific analyses showed positive model-estimated improvements across all exercise modalities, but the magnitude and modeled dose range differed substantially (Table 1; Figure 3).

Among the exercise modalities, AE-RT showed the largest estimated improvement at 1,500 MET-min/week (MD: 5.77 percentage points, 95% CrI: 4.04, 7.52). AE and RT showed similar estimated improvements, with AE reaching its largest estimate at 1,200 MET-min/week (MD: 4.15, 95% CrI: 2.53, 5.68) and RT at 900 MET-min/week (MD: 4.16, 95% CrI: 2.25, 6.05). HIIT showed a positive estimate at 1,000 MET-min/week (MD: 3.75, 95% CrI: 1.93, 5.65). MICT showed a smaller positive estimate within its observed dose range, whereas the MBE estimate was positive but its model-estimated optimum lay below the observed dose range and should be treated as an unreliable extrapolation. The detailed modality-specific dose ranges and estimates are presented in Table 1.

### 3.5 Treatment ranking and model-based prescription anchors

Under the dose-response network meta-analysis, all 168 modality-dose combinations were ranked according to their posterior mean ranks (Supplementary Table S7). AE-RT dominated the highest-ranked positions: AE-RT at 1,400 MET-min/week ranked first (posterior mean rank 6.12), followed by AE-RT at 1,500, 1,300, 1,600, and 1,200 MET-min/week (mean ranks 6.38, 6.99, 7.66, and 8.83, respectively). The next non-AE-RT modality to appear was RT at 900 MET-min/week (ranked 16th; mean rank 30.21), followed by AE at 1,200 MET-min/week (ranked 17th; mean rank 30.38). Placebo or no exercise ranked last at position 168 (mean rank 166.15). Lower posterior mean ranks indicate higher posterior probability of producing the largest improvement in brachial FMD.

**Figure 4:**
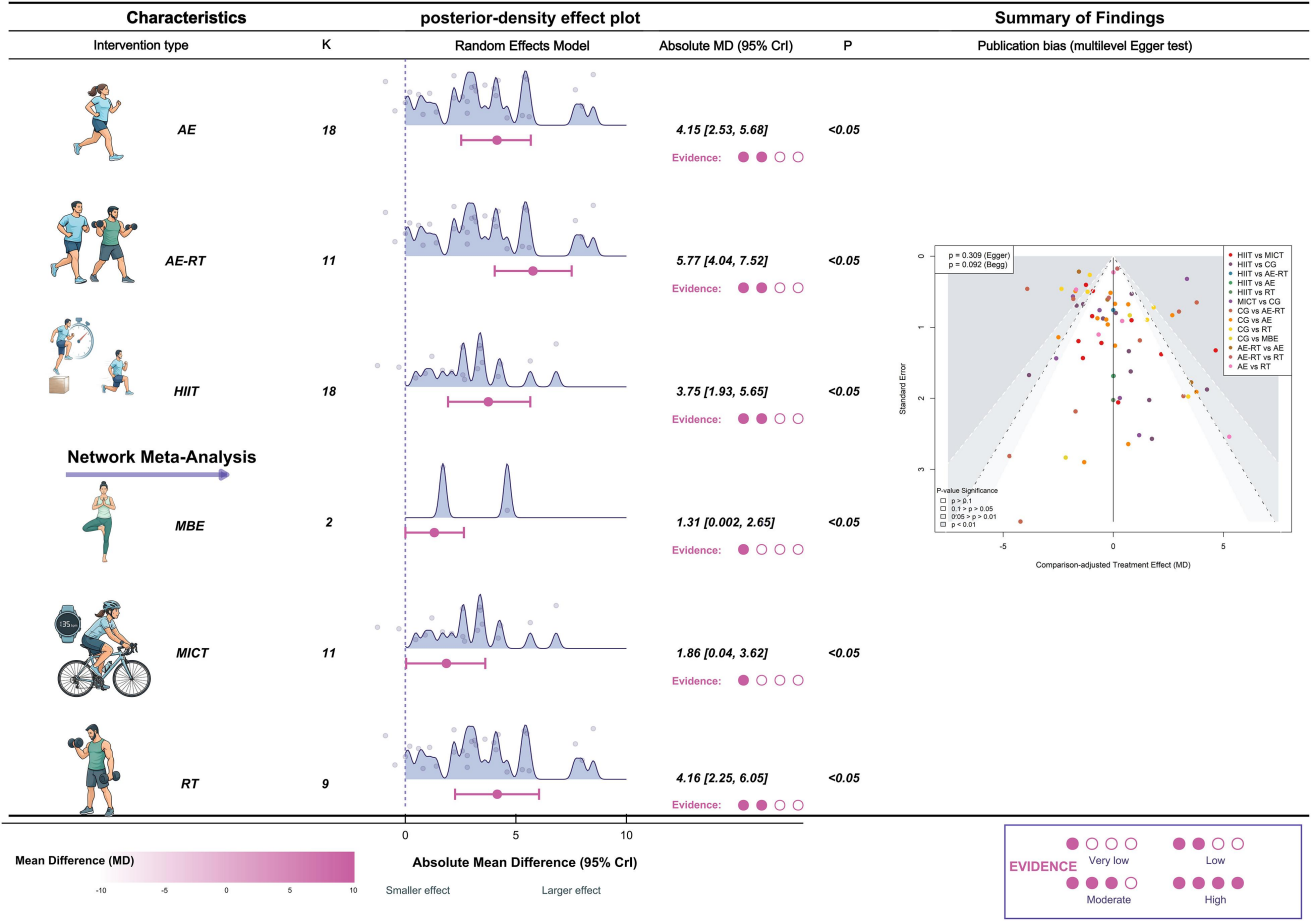
Network-based graphical evidence summary of modality-specific effects on brachial FMD. Each row presents one exercise modality with: K (arm-level data points), posterior-density effect plot (shaded violin = posterior density; point estimate = model-estimated mean difference; horizontal bar = 95% credible interval); absolute MD with 95% CrI; and CINeMA evidence ratings (4 filled circles = high to 1 = very low). The right inset shows the network plot and the comparison-adjusted funnel plot with Egger's test (*p* = 0.309) and Begg's test (*p* = 0.092). Positive values indicate improvement in brachial FMD. AE, aerobic exercise; AE-RT, combined aerobic and resistance training; RT, resistance training; HIIT, high-intensity interval training; MICT, moderate-intensity continuous training; MBE, mind-body exercise; brachial FMD, brachial artery flow-mediated dilation; MD, mean difference; CrI, credible interval.

For clinical interpretation, model-estimated MET-min/week doses were translated into approximate weekly minutes by exercise modality (Table 2). These translations were intended to aid clinical interpretation of the model-estimated dose values and should not be interpreted as fixed prescriptions or safety thresholds. Overall exercise at 1,500 MET-min/week corresponded approximately to 200 min/week cycling or 250 min/week jogging. For AE-RT, the model-estimated 1,500 MET-min/week dose could be translated into a combined prescription such as 105 min/week cycling plus 215 min/week resistance training, depending on the MET values assigned to each activity. These results suggest that AE-RT at weekly doses of approximately 1,200–1,600 MET-min/week was most consistently predicted to produce the largest brachial FMD response. A network-based graphical evidence summary integrating modality-specific estimates, certainty ratings, and small-study effect assessment is provided in Figure 4.

**Table 2:** Model-based exercise-dose translations for improving brachial FMD.

Outcome	Exercise modality	Energy expenditure (METs)^a^^,^^*^	Recommended accumulation (min/week)	Optimal recommended accumulation (min/week)	Certainty of evidence (grade)
Brachial FMD	Cycling	7 (code: 01016)^b^	∼170	200	Low
	Jogging	4.8(code:12025)^c^	∼290	250	Low
	Walking	3.5(code:17160)^d^	∼400	345	Low
	Resistance training	3.5(code:02054)^e^	∼255	255	Low
	Cycling combined with resistance training	7(code: 01016) 3.5(code:02054)	Cycling: ∼170; Resistance training: ∼345	Cycling: 105; Resistance training: 215	Low
	Jogging combined with resistance training	4.8(code:12025) 3.5(code:02054)	Jogging: ∼250; Resistance training: ∼345	Jogging: 155; Resistance training: 215	Low
	Yoga	2.3(code:02175)^f^	∼60	60	Very low
	HIIT	11.0(code:02214)^g^	∼90	90	Low

* Exercise intensity was coded based on the 2024 standardized physical activity intensity tables.

a METs: Metabolic equivalent of task.

b Cycling: self-selected moderate pace.

c Jogging, in place.

d Walking for pleasure.

e Resistance (weight) training, multiple exercises, 8–15 reps at varied resistance.

f Yoga: general.

g HIIT: High intensity interval exercise, burpees, mountain climbers, squat jumps, Tabata, vigorous effort. 3–6 METs represents moderate-intensity activity, and >6 METs represents high-intensity activity, serving as a reference for absolute exercise intensity.

### 3.6 Subgroup and sensitivity analyses

Subgroup analyses suggested broadly consistent dose-response patterns across baseline brachial FMD, BMI category, health status, and intervention duration. Among participants with baseline brachial FMD ≤6.5%, 60 data points contributed to the model, and the dose associated with the largest estimated improvement was 1,400 MET-min/week (MD: 4.30 percentage points, 95% CrI: 1.87, 6.85). Among participants with baseline brachial FMD >6.5%, 40 data points contributed, and the corresponding dose was 1,400 MET-min/week (MD: 5.94, 95% CrI: 3.86, 8.14).

For BMI category, participants with BMI 25.0–29.9 kg/m^2^ contributed 65 data points; the dose associated with the largest estimated improvement was 1,400 MET-min/week (MD: 4.99, 95% CrI: 3.71, 6.27). Participants with BMI ≥30 kg/m^2^ contributed 30 data points; the corresponding dose was 1,000 MET-min/week (MD: 3.51, 95% CrI: 0.53, 6.53). For health status, apparently healthy adults with overweight or obesity contributed 32 data points; the dose associated with the largest estimated improvement was 1,400 MET-min/week (MD: 5.49, 95% CrI: 2.22, 8.84). Adults with overweight or obesity and cardiometabolic disease contributed 75 data points; the corresponding dose was 1,800 MET-min/week (MD: 4.87, 95% CrI: 1.90, 7.88).

For intervention duration, trials lasting 4–8 weeks contributed 30 data points; the dose associated with the largest estimated improvement was 1,000 MET-min/week (MD: 5.10, 95% CrI: 1.37, 9.02). Trials lasting 10–12 weeks contributed 54 data points; the corresponding dose was 1,500 MET-min/week (MD: 3.82, 95% CrI: 2.16, 5.32). Trials lasting more than 12 weeks contributed 23 data points; the corresponding dose was 1,900 MET-min/week (MD: 6.33, 95% CrI: 0.63, 12.16).Several subgroup estimates were markedly imprecise: the >12-week credible interval (0.63 to 12.16 percentage points) spans effects ranging from trivial to very large, and similarly wide intervals were observed for the 4–8-week (1.37 to 9.02) and BMI ≥30 kg/m^2^ (0.53 to 6.53) strata. Subgroup curves are shown in Supplementary Figures S7–S15.

Sensitivity analysis excluding high-risk-of-bias trials included 98 effect-size data points. The dose associated with the largest estimated improvement remained 1,500 MET-min/week, with an MD of 4.84 percentage points (95% CrI 3.53, 6.05), consistent with the main analysis (Supplementary Figure S16).

### 3.7 Meta-regression

Meta-regression did not identify statistically clear effect modification by the four examined study-level covariates. The regression coefficient for age was *β* = −0.07 (95% CrI: −0.18, 0.05; DIC: 360.1). For baseline brachial FMD, *β* = 0.23 (95% CrI: −0.17, 0.61; DIC: 369.3). For intervention length, *β* = −0.13 (95% CrI: −0.29, 0.02; DIC: 350.1). For baseline BMI, *β* = −0.15 (95% CrI: −0.47, 0.15; DIC: 332.4). Because all 95% CrIs included 0, none of these covariates showed sufficient evidence of modifying the dose-response relationship (Supplementary Table S11).

### 3.8 Certainty of evidence and publication bias

CINeMA assessment covered 13 network comparisons. Ten comparisons were rated as low certainty and three as very low certainty, and no comparison reached moderate or high certainty. Very low certainty was mainly assigned to MBE- and MICT-related comparisons. The main reasons for downgrading were imprecision, heterogeneity, incoherence, and within-study bias in selected comparisons. Detailed CINeMA judgments are summarized in Table 3 and provided in full in Supplementary Table S9 and Figure S18.

**Table 3:** Summary of evidence (certainty of the network estimates).

Comparison	Certainty (CINeMA)	Main reason(s) for downgrading
AE vs. AE-RT	Low	Imprecision
AE vs. CG	Low	Heterogeneity
AE vs. HIIT	Low	Imprecision
AE vs. RT	Low	Imprecision
AE-RT vs. CG	Low	Heterogeneity
AE-RT vs. HIIT	Low	Imprecision
AE-RT vs. RT	Low	Imprecision
HIIT vs. CG	Low	Heterogeneity
HIIT vs. RT	Low	Imprecision
RT vs. CG	Low	Heterogeneity
MICT vs. CG	Very low	Within-study bias; heterogeneity
MBE vs. CG	Very low	Within-study bias; imprecision; incoherence
HIIT vs. MICT	Very low	Within-study bias; imprecision; incoherence

All 13 network comparisons were rated low (*n* = 10) or very low (*n* = 3) certainty (CINeMA); none reached moderate or high certainty. CG, control group; other abbreviations as in Table 1. Full domain-level judgments are provided in Supplementary Table S9.

Comparison-adjusted funnel plot inspection did not show marked asymmetry (Supplementary Figure S17). Using a significance threshold of *p* < 0.05, neither Egger's test *p* = 0.309 nor Begg's test *p* = 0.092 indicated significant small-study effects, although the Begg *p*-value was close to this threshold. Because few studies contributed to each comparison, these tests have limited statistical power, so the absence of significance does not exclude small-study effects. In a trim-and-fill sensitivity analysis of the pairwise exercise-vs.-control comparison, two studies were imputed and the adjusted pooled estimate (2.84, 95% CI: 2.15 to 3.53 percentage points) was essentially unchanged from the unadjusted estimate (2.95, 95% CI: 2.22 to 3.67), suggesting that the auxiliary pairwise estimate was not materially changed by trim-and-fill adjustment.

## 4 Discussion

### 4.1 Principal findings and implications

To our knowledge, no previous review has applied a Bayesian dose-response network meta-analysis to simultaneously examine exercise modality, weekly MET-min/week dose, and brachial FMD in adults with overweight or obesity. By synthesizing evidence across different exercise types and dose levels, this study provides provisional, model-based prescription anchors that, given the low-to-very-low certainty of the underlying evidence, may help inform rather than direct exercise-based strategies for improving endothelial function in this population. Overall exercise showed a positive dose-response association with brachial FMD, with the largest model-estimated improvement observed at 1,500 MET-min/week (MD: 4.72 percentage points, 95% CrI: 3.43, 5.91). When stratified by modality, AE-RT showed the largest estimated improvement at 1,500 MET-min/week (MD: 5.77 percentage points, 95% CrI: 4.04, 7.52), and the modality-dose ranking identified AE-RT at approximately 1,200–1,600 MET-min/week as the most consistently highest-ranked prescription range.

From a practical standpoint, this matters because exercise advice for adults with overweight or obesity is usually built around broad weekly activity targets and rarely says which modality or dose is most favorable for vascular function (36, 37). In our data, 1,500 MET-min/week works out to roughly 200 min/week of cycling, 250 min/week of jogging, or a combined AE-RT plan such as 105 min/week of cycling alongside 215 min/week of resistance training, with the exact figures depending on the MET value assigned to each activity. These numbers are best read as model-estimated anchors, not fixed thresholds. In everyday practice they would still have to be tuned to a person's baseline fitness, comorbidities, medication use, exercise tolerance, and preferences.

The subgroup analyses pointed to several clinically relevant signals. A positive dose-response pattern appeared in both baseline brachial FMD strata, which suggests that exercise can benefit endothelial function regardless of where vascular function starts. Participants with a BMI of 25.0 to 29.9 kg/m^2^ had a larger estimated response than those at 30 kg/m^2^ or above, possibly because more severe obesity carries a heavier metabolic and inflammatory load. Positive responses also showed up in both the apparently healthy group and the group with cardiometabolic disease, though the latter needed a higher weekly dose to reach its largest estimated improvement. By intervention length, the effect stayed positive across 4 to 8, 10 to 12, and beyond 12 weeks, but several subgroup credible intervals were very wide. This was most striking for trials lasting more than 12 weeks (MD: 6.33, 95% CrI: 0.63 to 12.16 percentage points), where the true effect could range from trivial to large; the corresponding point estimates should therefore not be over-interpreted. Given that data points were spread unevenly across several subgroups and that dichotomizing continuous variables can lose information and inflate error, these subgroup results are exploratory; in particular, the strata informed by fewer than 15 trials (BMI ≥30 kg/m^2^, 14 trials; 4 to 8 weeks, 13 trials; >12 weeks, 11 trials) should be considered unreliable and are reported for completeness only (38).

Dropping the high-risk-of-bias trials in the sensitivity analysis left the result close to the main estimate, which speaks to its stability. Meta-regression turned up no clear effect modification by age, baseline brachial FMD, intervention length, or baseline BMI. That is not to say these factors do not shape how an individual responds. It more likely reflects that the study-level data on hand were too limited to reveal any clear modification of the modeled dose-response relationship.

### 4.2 Interpretation and comparison with existing literature

Previous reviews have shown that exercise training improves endothelial function in adults, including populations with heart failure, type 2 diabetes, and other cardiometabolic disorders (16, 17, 23, 39). Our analysis pushes this work forward on two fronts. The first is its focus: it deals specifically with adults who are overweight or obese, a group in whom endothelial dysfunction sits close to insulin resistance, low-grade inflammation, oxidative stress, and downstream cardiometabolic risk (1–3). The second is its method: rather than asking simply whether exercise helps, it carries weekly dose through the network as a continuous exposure.

Ashor et al. reported pooled weighted mean differences in FMD of 2.79 percentage points for aerobic exercise, 2.52 for resistance training, and 2.07 for combined training across an unrestricted dose range, with aerobic exercise showing the largest effect (23). In contrast, our dose-response analysis tells a somewhat different story. At each modality's best-performing dose the estimated effects were larger (AE reached an MD of 4.15 percentage points at 1,200 MET-min/week, and AE-RT reached 5.77 at 1,500 MET-min/week), and the ordering shifted as well, with combined training rising to the top of the modality-dose hierarchy. The contrast makes sense once the two approaches are seen for what they ask. A pooled estimate averages over interventions that were underdosed, well dosed, and perhaps pushed too far, while dose-response modeling asks the more prescription-relevant question of where along the dose range each modality looks strongest in the available evidence.

These larger model-estimated effects should be interpreted cautiously. Unlike pooled estimates from previous meta-analyses, the present estimates reflect predicted responses at specific weekly dose values within the observed evidence base, rather than average effects across all exercise prescriptions. The difference from prior pooled estimates may partly reflect lower baseline endothelial function in some trials, variation in brachial FMD measurement protocols, small-study influences, and the tendency of dose-response models to identify the most favorable portion of the observed dose range. Therefore, the estimated improvements of 4–5 percentage points should be viewed as physiologically plausible under selected study conditions, but potentially optimistic as generalizable clinical effects.

The finding that AE-RT ranked highest is also physiologically coherent from an exercise physiology perspective. Aerobic exercise repeatedly increases conduit artery blood flow and shear stress, a stimulus that may enhance nitric oxide-dependent vasodilatory signaling and endothelial adaptation, while resistance training may improve skeletal muscle perfusion, insulin sensitivity, blood pressure regulation, and peripheral vascular remodeling (39–41). Combining these modalities may therefore provide a broader vascular and metabolic stimulus than either component alone.

Metabolic and inflammatory pathways may also contribute to the vascular response to exercise. In adults with obesity or type 2 diabetes, physical activity and resistance-based training have been linked to changes in adipokines, insulin resistance, inflammatory cytokines, and skeletal-muscle- or liver-derived factors. Prior work has suggested that exercise may influence adipokine profiles in type 2 diabetes, Meteorin-like protein, fetuin-B, retinol-binding protein 4, tumor necrosis factor-alpha, gremlin 1, macrophage migration inhibitory factor, and WISP proteins (4–6, 42–44). These biomarkers are not direct measures of brachial FMD, but they support the biological plausibility that exercise, particularly resistance or combined training, may improve the metabolic and inflammatory milieu in which endothelial function is regulated. This interpretation fits with the broader “exercise as medicine” literature, which emphasizes that different exercise modes act through partly overlapping but not identical physiological pathways (45, 46).

The comparison between HIIT and MICT also deserves attention from an exercise physiology perspective. HIIT may produce larger intermittent changes in blood flow, shear stress, sympathetic activation, and metabolic demand than steady continuous exercise, which could partly explain its larger model-estimated effect. Ramos et al. reported that HIIT may produce greater vascular benefits than MICT in some settings (18). In our analysis, HIIT showed a larger estimated improvement than MICT, but both modalities had 95% CrIs above 0 at their model-estimated doses. Therefore, the result should not be interpreted as showing that MICT is ineffective. A more cautious reading is that HIIT may achieve a larger brachial FMD response within the observed dose range, whereas the MICT evidence was concentrated within a narrower weekly dose range. Similar caution applies to MBE, where the estimated effect was positive but based on fewer data points, lower external dose coverage, and a limited observed dose range.

Mechanistically, the dose-dependent pattern is consistent with shear stress acting as the principal stimulus for endothelial adaptation. Repeated bouts of exercise raise conduit-artery shear rate, which upregulates endothelial nitric oxide synthase and improves nitric oxide bioavailability, while regular training also attenuates oxidative stress and thereby protects nitric oxide from degradation (40, 47). As weekly dose increases, this cumulative shear-stress and antioxidant stimulus accumulates, which may account for the rising portion of the dose-response curve; at the highest volumes, diminishing returns, incomplete recovery, or transient increases in oxidative stress may blunt further gains, consistent with the plateau observed at higher doses. Differences between modalities plausibly reflect the pattern of shear that each delivers: combined aerobic and resistance training superimposes resistance-induced increases in muscle perfusion and metabolic demand on the aerobic shear stimulus, whereas high-intensity interval training produces larger intermittent shear pulses than steady moderate-intensity exercise.

The subgroup findings also align with what is known about vascular dysfunction in metabolic disease. Adults with obesity, diabetes, hypertension, or established cardiovascular disease often have more advanced endothelial impairment, and exercise-induced improvements may depend on both the baseline vascular state and the amount of repeated hemodynamic stimulus delivered (39, 40, 47, 48). This may explain why the cardiometabolic disease subgroup showed its largest estimated improvement at a higher weekly dose than the apparently healthy subgroup. At the same time, the data do not allow us to say that higher-risk patients necessarily require more exercise. Medication use, baseline fitness, disease duration, and measurement protocols were not evenly reported across trials, and these factors may have influenced the subgroup estimates.

### 4.3 Limitations

This study has several limitations. To begin with, any dose-response meta-analysis is only as good as the data feeding it and the model chosen to fit them. The quadratic random-effects model fit best overall, but data thinned out at the higher doses, especially for RT, MICT, and MBE, so estimates past the observed dose range remain exploratory. The edge that AE-RT appears to hold is therefore best taken as the pattern suggested by currently available, low-certainty evidence, not as a settled prescription rule.

Heterogeneity is a second concern. We used subgroup and meta-regression analyses to probe it, but the trials still varied in participants' health status, medication use, baseline vascular function, degree of exercise supervision, adherence, and how the intervention was delivered. In addition, three of the included trials paired exercise with a dietary or weight-loss co-intervention while the remainder did not; although the eligibility criteria ensured that any co-intervention was balanced across arms within each trial, its presence in a small number of trials remains a minor additional source of between-trial heterogeneity. The samples ran from apparently healthy adults with overweight or obesity to people with hypertension, type 2 diabetes, coronary artery disease, heart failure, metabolic syndrome, and related conditions. That spread mirrors real clinical practice, yet it also caps how precise any modality- or subgroup-specific conclusion can be.

Health-status subgroup analyses partly addressed this issue by separating apparently healthy adults with overweight or obesity from those with cardiometabolic disease, including cardiac and metabolic conditions. The model-estimated dose associated with the largest improvement differed between these strata, occurring at 1,400 MET-min/week in apparently healthy participants and 1,800 MET-min/week in those with cardiometabolic disease. This difference may reflect greater baseline vascular impairment or different exercise tolerance in clinical populations, but it should not be interpreted as evidence that patients with cardiometabolic disease necessarily require higher exercise doses. Disease severity, volume status, medication use, and baseline fitness were not consistently reported and may have influenced these subgroup estimates. Accordingly, the overall estimate of 1,500 MET-min/week should not be applied directly to patients with heart failure or other cardiovascular disease without consideration of disease severity, functional capacity, contraindications, and clinical supervision. Age, BMI, and baseline brachial FMD were broadly comparable across treatment nodes, whereas the proportion of female participants was less evenly distributed and the mind-body exercise node comprised only two, predominantly female arms; comparisons involving mind-body exercise and, to a lesser extent, moderate-intensity continuous training were therefore the most susceptible to transitivity violations, consistent with their very-low certainty ratings. Although these measured effect modifiers did not show statistically significant imbalance across treatment groups, transitivity could still have been affected by other incompletely reported clinical factors.

The reliance on BMI to define overweight or obesity is another limitation. BMI was the most consistently available eligibility measure across the included trials, but it is an indirect marker of adiposity and does not distinguish fat mass from lean mass or capture fat distribution. This limitation is particularly relevant for heart failure populations, in whom fluid retention or edema may increase body weight and BMI independently of adiposity. The relationship between BMI and adiposity may also vary by sex, age, ethnicity, and country. Because more direct body-composition or fat-distribution measures were not consistently available, we could not examine whether exercise effects differed according to body fat percentage, waist circumference, waist-to-hip ratio, or imaging-derived fat mass.

Measurement of brachial FMD is another issue. Brachial FMD protocols were not uniform across studies, differing in cuff position, occlusion time, image acquisition, and edge-detection procedures, and these methodological choices can move FMD values around and add to between-study variability (8, 9). This is particularly relevant when interpreting the larger model-estimated improvements, because protocol variation may affect the absolute magnitude of brachial FMD responses across trials. A further limitation concerns the dose metric itself. MET-min/week provides a practical way to standardize prescribed external exercise load, but it does not fully capture the internal physiological stimulus generated by different exercise modalities. In addition, assigning a single MET value to heterogeneous protocols introduces measurement error, which is greatest for resistance training, whose energy cost varies with load, volume, and rest intervals that the Compendium does not capture. Aerobic exercise, resistance training, HIIT, mind-body exercise, and combined training differ in vascular shear stress, autonomic activation, metabolic demand, muscle recruitment, and post-exercise hemodynamic responses. The same MET-min/week dose may therefore produce different endothelial stimuli across modalities and across individuals with different fitness levels or cardiometabolic profiles. This limitation is particularly relevant when interpreting the apparent advantage of AE-RT, because combined training may accumulate a larger or more diverse external dose while still producing vascular stimuli that are not fully interchangeable with those generated by single-modality exercise.Furthermore, because MET-min/week reflects the prescribed rather than the completed exercise dose, and adherence was reported in only about half of the trials, the dose actually received by participants may have differed from that prescribed; incomplete or unreported adherence would tend to attenuate the true dose-response relationship.

Several further constraints should be noted. We included only parallel-group randomized trials and excluded crossover designs; although this avoids potential carryover effects, it may have reduced the precision obtainable from the available evidence. Because the included trials assessed brachial FMD after a training period, our findings reflect chronic training adaptations and cannot be used to distinguish them from the acute vascular responses that follow a single exercise session. In addition, exercise-induced weight loss, which was not consistently reported and may vary across modalities and doses, could have contributed to the observed improvements independently of the direct vascular effects of exercise.

Where change-score SDs were missing, we imputed them with a prespecified correlation of 0.5, which will have added some uncertainty of its own. The certainty of evidence was also generally low to very low, driven mainly by imprecision, heterogeneity, incoherence, and within-study bias in particular comparisons. One last caveat concerns reporting bias. The comparison-adjusted funnel plot showed no marked asymmetry, and neither Egger's test p=0.309 nor Begg's test p=0.092 reached the *p* < 0.05 threshold, although the Begg value was close to it. These tests have low power when few studies contribute to each comparison, so a non-significant result does not rule out reporting bias; however, a trim-and-fill sensitivity analysis imputed only two studies and left the pooled estimate essentially unchanged. Publication bias and small-study effects therefore cannot be fully excluded but do not appear to have materially distorted the overall finding.

## 5 Conclusions

In adults with overweight or obesity, exercise was associated with dose-dependent improvement in brachial FMD. Among the modeled modality-dose combinations, AE-RT around 1,500 MET-min/week showed the most favorable profile, with AE-RT at approximately 1,200–1,600 MET-min/week consistently ranked among the highest predicted responses. These findings may help translate MET-min/week estimates into approximate weekly exercise volumes, but they should be used as provisional model-based anchors rather than fixed clinical thresholds and should be individualized according to clinical status, exercise tolerance, and safety. Interpretation should remain cautious because the certainty of evidence was low to very low, several modality-dose combinations were sparsely informed, and the model-estimated MBE optimum lay outside the observed dose range. Future head-to-head randomized trials using standardized brachial FMD protocols and prespecified dose levels are needed before these estimates can support firm clinical or public health recommendations.

## Data Availability

The original contributions presented in the study are included in the article/Supplementary Material, further inquiries can be directed to the corresponding author.
