# Supplementary material for: Effects of exercise modalities and doses on brachial artery flow-mediated dilation in adults with overweight or obesity: a Bayesian dose-response network meta-analysis
Source: Front Cardiovasc Med. 2026 Jul 23;13:1916772. doi: 10.3389/fcvm.2026.1916772 (PMC13441802; doi:10.3389/fcvm.2026.1916772)
Supplement: Supplementary file 1 [file Datasheet1.pdf]

Supplementary content

Supplementary content 1
1. Search syntax for each database 1
1.1 Pubmed(578) 1
1.2 Web of Science(1621) 1
1.3 Embase(1550) 2
1.4 Cochrane(1823) 2
1.5 Scopus(1750) 3
1.6 SPORTDiscus(260) 3
1.7 ClinicalTrials.gov(189) 4
2. Definitions of exercise modalities 5
3. Study characteristics 6
4. Supplementary methods 14
5.Risk of bias 16
6. Dose-response network meta-analyses 17
6.1 Connectivity 17
6.2 Consistency 18
6.3 Inconsistency assessment 18
6.4 Model selection 23
6.5 Subgroup analysis 26
6.6 Sensitivity analysis 32
6.7 Effectiveness ranking of different exercises and doses 33
6.8 Meta-regression 37
8. Publication bias 38
9. GRADE Summary of All Studies 38
9.1 Within-study Bias 38
9.2 Reporting Bias 38
9.3 Indirectness 39
9.4 Imprecision 39
9.5 Heterogeneity 39
9.6 Incoherence 39
9.7 Summarizing Judgments Across CINeMA Domains 39
10.Network transitivity assessment 44
Reference 47

1. Search syntax for each database

Search strategies were designed to maximize sensitivity. Terms for overweight and obesity were not mandatory in the electronic searches because many potentially eligible cardiometabolic trials did not report BMI status in titles or abstracts; population eligibility was determined during title/abstract and full-text screening.

1.1 Pubmed(578)

("flow-mediated dilation"[Title/Abstract] OR"flow mediated dilation"[Title/Abstract] OR"flow-mediated dilatation"[Title/Abstract] OR"flow mediated dilatation"[Title/Abstract] OR FMD[Title/Abstract] OR"endothelial function"[Title/Abstract] OR"endothelium-dependent vasodilation"[Title/Abstract] OR"endothelium dependent vasodilation"[Title/Abstract] OR"vascular function"[Title/Abstract])

AND

("Exercise"[Mesh] OR"Exercise Therapy"[Mesh] OR "Motor Activity"[Mesh] OR"Sports"[Mesh] OR"Resistance Training"[Mesh] OR "Yoga"[Mesh] OR "Tai Ji"[Mesh] OR "Walking"[Mesh] OR "Physical Exertion"[Mesh] OR exercise[Title/Abstract] OR training[Title/Abstract] OR "physical activity"[Title/Abstract] OR "aerobic exercise"[Title/Abstract] OR "resistance training"[Title/Abstract] OR "strength training"[Title/Abstract] OR "combined training"[Title/Abstract] OR "concurrent training"[Title/Abstract] OR "high-intensity interval training"[Title/Abstract] OR HIIT[Title/Abstract] OR MICT[Title/Abstract] OR "endurance training"[Title/Abstract] OR yoga[Title/Abstract] OR "Tai Chi"[Title/Abstract] OR qigong[Title/Abstract] OR pilates[Title/Abstract] OR walking[Title/Abstract] OR cycling[Title/Abstract] OR treadmill[Title/Abstract])

AND

(randomized controlled trial[Publication Type] OR controlled clinical trial[Publication Type] OR randomized[Title/Abstract] OR randomised[Title/Abstract] OR randomly[Title/Abstract] OR trial[Title/Abstract] OR "controlled trial"[Title/Abstract] OR"control group"[Title/Abstract])

NOT

(animals[MeSH Terms] NOT humans[MeSH Terms])

1.2 Web of Science(1621)

(TS= ("flow-mediated dilation" OR"flow mediated dilation" OR"flow-mediated dilatation" OR"flow mediated dilatation" ORFMD OR"endothelial function" OR"endothelium-dependent vasodilation" OR"endothelium dependent vasodilation" OR"vascular function"))

AND

(TS=(exercise ORtraining OR"physical activity" OR"aerobic exercise" OR"resistance training" OR"strength training" OR"combined training" OR"concurrent training" OR"high-intensity interval training" ORHIIT ORMICT OR"endurance training" ORyoga OR"Tai Chi" OR qigong OR pilates OR walking OR cycling OR treadmill))

AND

(TS=(random* OR trial* OR"controlled trial" OR"control group"))

1.3 Embase(1550)

('flow mediated dilation':ti,ab,kw OR 'flow-mediated dilation':ti,ab,kw OR' flow mediated dilatation':ti,ab,kw OR 'flow-mediated dilatation':ti,ab,kw OR fmd:ti,ab,kw OR 'endothelial function':ti,ab,kw OR 'endothelium dependent vasodilation':ti,ab,kw OR 'vascular function':ti,ab,kw)

AND

('exercise'/exp OR 'exercise therapy'/exp OR 'physical activity'/exp OR 'resistance training'/exp OR 'aerobic exercise'/exp OR 'high intensity interval training'/exp OR 'yoga'/exp OR 'tai chi'/exp OR 'walking'/exp OR exercise:ti,ab,kw OR training:ti,ab,kw OR 'physical activity':ti,ab,kw OR 'aerobic exercise':ti,ab,kw OR 'resistance training':ti,ab,kw OR 'strength training':ti,ab,kw OR 'combined training':ti,ab,kw OR 'concurrent training':ti,ab,kw OR 'high-intensity interval training':ti,ab,kw OR hiit:ti,ab,kw OR mict:ti,ab,kw OR 'endurance training':ti,ab,kw OR yoga:ti,ab,kw OR 'tai chi':ti,ab,kw OR qigong:ti,ab,kw OR pilates:ti,ab,kw ORwalking:ti,ab,kw OR cycling:ti,ab,kw OR treadmill:ti,ab,kw)

AND

('randomized controlled trial'/exp OR 'controlled clinical trial'/exp OR random*:ti,ab,kw OR trial*:ti,ab,kw OR 'controlled trial':ti,ab,kw OR 'control group':ti,ab,kw)

NOT

('animal'/exp NOT 'human'/exp)

1.4 Cochrane(1823)

#1 ("flow-mediated dilation" OR "flow mediated dilation" OR "flow-mediated dilatation" OR "flow mediated dilatation"):ti,ab,kw

#2 (FMD OR "brachial FMD" OR "brachial artery FMD"):ti,ab,kw

#3 ("endothelial function" OR "endothelium-dependent vasodilation" OR "endothelium dependent vasodilation"):ti,ab,kw

#4 ("vascular function" OR "vascular endothelial function" OR "arterial function"):ti,ab,kw

#5 #1 OR #2 OR #3 OR #4

#6 MeSH descriptor: [Exercise] explode all trees

#7 MeSH descriptor: [Exercise Therapy] explode all trees

#8 MeSH descriptor: [Motor Activity] explode all trees

#9 MeSH descriptor: [Sports] explode all trees

#10 MeSH descriptor: [Resistance Training] explode all trees

#11 MeSH descriptor: [Yoga] explode all trees

#12 MeSH descriptor: [Tai Ji] explode all trees

#13 MeSH descriptor: [Walking] explode all trees

#14 MeSH descriptor: [Physical Exertion] explode all trees

#15 (physical activity OR movement OR fitness OR exercise OR training):ti,ab,kw

#16 ("aerobic exercise" OR "aerobic training" OR "endurance exercise" OR "endurance training"):ti,ab,kw

#17 ("high-intensity interval training" OR "high intensity interval training" OR HIIT OR MICT OR "moderate-intensity continuous training" OR "moderate intensity continuous training"):ti,ab,kw

#18 ("resistance training" OR "strength training" OR "weight training" OR "circuit training"):ti,ab,kw
#19 ("combined training" OR "concurrent training" OR "combined exercise" OR "aerobic and resistance" OR "aerobic plus resistance"):ti,ab,kw
#20 (yoga OR "tai chi" OR taiji OR qigong OR pilates OR dance):ti,ab,kw
#21 (walking OR jogging OR running OR cycling OR treadmill OR swimming OR stretching OR "mind-body exercise"):ti,ab,kw
#22 #6 OR #7 OR #8 OR #9 OR #10 OR #11 OR #12 OR #13 OR #14 OR #15 OR #16 OR #17 OR #18 OR #19 OR #20 OR #21
#23 MeSH descriptor: [Randomized Controlled Trial] explode all trees
#24 (randomized OR randomised OR randomly OR RCT OR RCTs OR "randomized trial" OR "randomised trial" OR "randomized controlled trial" OR "randomised controlled trial" OR "randomized clinical trial" OR "randomised clinical trial" OR "controlled clinical trial" OR "controlled trial" OR "randomized study" OR "randomised study" OR "intervention trial" OR "randomized intervention" OR "randomised intervention" OR trial OR "experimental study" OR "control group"):ti,ab,kw
#25 #23 OR #24
#26 #5 AND #22 AND #25

1.5 Scopus(1750)

TITLE-ABS-KEY(("flow-mediated dilation" OR"flow mediated dilation" OR "flow-mediated dilatation" OR "flow mediated dilatation" OR FMD OR "endothelial function" OR "endothelium-dependent vasodilation" OR "endothelium dependent vasodilation" OR "vascular function")

AND

(exercise OR training OR "physical activity" OR "aerobic exercise" OR "resistance training" OR "strength training" OR "combined training" OR "concurrent training" OR "high-intensity interval training" OR HIIT OR MICT OR "endurance training" OR yoga OR "Tai Chi" OR qigong OR pilates OR walking OR cycling OR treadmill)

AND

(random* OR trial* OR "controlled trial" OR "control group"))

1.6 SPORTDiscus(260)

(TI ("flow-mediated dilation" OR "flow mediated dilation" OR "flow-mediated dilatation" OR "flow mediated dilatation" OR FMD OR "endothelial function" OR "endothelium-dependent vasodilation" OR "vascular function")OR AB ("flow-mediated dilation" OR "flow mediated dilation" OR "flow-mediated dilatation" OR "flow mediated dilatation" OR FMD OR "endothelial function" OR "endothelium-dependent vasodilation" OR "vascular function"))

AND

(TI (exercise OR training OR "physical activity" OR "aerobic exercise" OR "resistance training" OR "strength training" OR "combined training" OR "concurrent training" OR "high-intensity interval training" OR HIIT OR MICT OR "endurance training" OR yoga OR "Tai Chi" OR qigong OR pilates OR walking OR cycling OR treadmill)

OR
AB (exercise OR training OR "physical activity" OR "aerobic exercise" OR"resistance training" OR "strength training" OR "combined training" OR "concurrent training" OR "high-intensity interval training" OR HIIT OR MICT OR "endurance training" OR yoga OR "Tai Chi" OR qigong OR pilates OR walking OR cycling OR treadmill))
AND
(TI (random* OR trial* OR "controlled trial" OR "control group")
OR
AB (random* OR trial* OR "controlled trial" OR "control group"))

1.7 ClinicalTrials.gov(189)
("flow-mediated dilation" OR"flow mediated dilation" OR FMD OR "endothelial function" OR "vascular function")
AND
(exercise OR training OR "physical activity" OR aerobic OR "resistance training" OR "strength training" OR "high-intensity interval training" OR HIIT OR yoga OR "Tai Chi" OR qigong OR pilates OR walking OR cycling OR treadmill)

2. Definitions of exercise modalities

Table S1. Definitions of exercise modalities

Abbreviation	Full name	Definitions
AE	Aerobic Exercise	Aerobic exercise is performed by repeating sequences of light-to-moderate intensity activities for extended periods of time. e.g., walking, bicycle, and treadmill training etc.
CG	Control group	Non-exercise intervention, usual care, or health education
MBE	Mind-body exercises	A form of exercise that combines body movement, mental focus, and controlled breathing to improve strength, balance, flexibility, and overall health. Examples of mind -body exercises are yoga, tai chi, and qigong.
RT	Resistance Training	Exercise training designed to improve the strength, power, endurance and size of skeletal muscles.
AE-RT	Aerobic combined resistance training	During the same training phase or cycle, a training regimen combines aerobic exercises designed to enhance cardiopulmonary function with resistance training aimed at improving muscle strength and endurance.
HIIT	High-intensity intermittent	It typically consists of alternating high-intensity exercise lasting 15 seconds to 4 minutes with passive or low-intensity recovery periods. The heart rate during the high-intensity phase approaches 80% to 95% of the maximum heart rate.
MICT	Moderate-Intensity Continuous Training	Moderate-intensity continuous training is continuous aerobic exercise performed at moderate intensity without planned high-intensity intervals.

3. Study characteristics

Table S2. Study characteristics

Study	Interventions (n)	Length (week)	Age (mean±sd)	BMI	Adherence (%)	Country	Test_site	Health status	Frequency (times/week)	Duration (min)	Exercise intensity	Female percent (%)
Afousi et al. 2018 [1]	HIIT(18)	12	54.78(6.19)	29.38(0.93)	70	Iran	brachial artery	T2D + hypertension	3	62	85-90% HRmax (work) / 55-60% HRmax (recovery)	51.9
	MICT(17)	12	53.12(4.84)	28.93(1.02)	70	Iran	brachial artery	T2D + hypertension	3	42	70% HRmax	
	CG(17)	12	54.24(5.61)	29.29(1.30)	NR	Iran	brachial artery	T2D + hypertension	0	0	NA	
Alvarez et al. 2024-A [2]	AE-RT(10)	6	44.8(12.8)	28.4(4.9)	NR	Chile	brachial artery	Hypertension	3	10	80-100% HRpeak	NR
	CG(10)	6	44.8(12.8)	28.4(4.9)	NR	Chile	brachial artery	Hypertension	0	0	NA	
Alvarez et al. 2024-B [2]	AE-RT(10)	6	37.5(11.6)	26.0(2.2)	NR	Chile	brachial artery	Normotensive	3	10	80-100% HRpeak	NR
	CG(10)	6	37.5(11.6)	26.0(2.2)	NR	Chile	brachial artery	Normotensive	0	0	NA	
Anagnostakou et al. 2011 [3]	AE(14)	12	52(11)	28.6(4.6)	68	Greece	brachial artery	Heart failure	3	40	50% of peak workload at SRT (Watt-based)	17.9
	AE-RT(14)	12	54(10)	28.1(3.8)	68	Greece	brachial artery	Heart failure	3	40	interval @ 50% SRT_W; RT 55-65% 2RM / 10RM	
Azadpour et al. 2017 [4]	MICT(12)	10	57.58(4.29)	32.15(1.78)	NR	Iran	brachial artery	Postmenopausal obesity + prehypertension	3	35	50-70% HRR	100

	CG(12)	10	56.58(4.17)	31.29(1.40)	NR	Iran	brachial artery	Postmenopausal obesity + prehypertension	0	0	NA	
Barone Gibbs et al. 2012 [5]	AE-RT(49)	26	58(5)	32.3(5.3)	94	USA	brachial artery	T2D + hypertension	3	45	aerobic 60-90% HRmax; resistance 50% 1RM	38.4
	CG(63)	26	56(6)	33.5(4.3)	NR	USA	brachial artery	T2D + hypertension	0	0	NA	
Benda et al. 2015 [6]	HIIT(10)	12	63(8)	28.1(7.5)	100	Netherlands	brachial artery	Heart failure	2	10	90% of maximal workload	17.2
	MICT(10)	12	64(8)	28.9(4.7)	100	Netherlands	brachial artery	Heart failure	2	30	60-75% of maximal workload	
Bircher et al. 2007 [7]	AE(13)	12	50.1(7.8)	33.0(6.1)	90	Germany	brachial artery	Obesity	3	45	Highest fat oxidation intensity (~53% VO2max)	50
	CG(13)	12	51.8(7.5)	34.8(3.6)	NR	Germany	brachial artery	Obesity	0	0	NA	
Boeno et al. 2020 [8]	AE(15)	12	45.8(6.8)	32.9(4.5)	NR	Brazil	brachial artery	Hypertension	3	45	60% HRR -80% HRR (progressive)	54.8
	RT(15)	12	46.1(7.2)	31.5(4.5)	NR	Brazil	brachial artery	Hypertension	3	45	60% HRR -80% HRR (progressive)	
Bouaziz et al. 2019 [9]	HIIT(27)	10	72.9(2.5)	28.7(5.6)	94.7	France	brachial artery	Older adults with chronic disease	2	30	4 min @VT1 + 1 min @40% VT1	73.3
	CG(29)	10	74.3(3.4)	28.8(5.1)	NR	France	brachial artery	Older adults with chronic disease	0	0	NA	
Braith et al. 2008 [10]	AE(9)	12	54.4(13.1)	25.5(3.6)	NR	USA	brachial artery	Heart transplant recipients	3	37.5	RPE 11-13 (6-20 scale)	18.8
	CG(7)	12	54.3(9.5)	28.5(1.4)	NR	USA	brachial artery	Heart transplant recipients	0	0	NA	

Choi et al. 2023 [11]	AE-RT(15)	12	39.70(10.07)	25.46(4.32)	85	Korea	brachial artery	Obesity	aerobic 5 / resistance 2 / flexibility 5	aerobic50/ resistance60/ flexibility10	aerobic 60-85% HRmax; resistance OMNI 7-8	100
	CG(15)	12	39.20(11.63)	26.04(4.58)	NR	Korea	brachial artery	Obesity	0	0	NA	
Collins et al. 2023 [12]	MICT(15)	12	51.1(5.7)	30.6(3.4)	90	Australia	brachial artery	Sedentary overweight/obese adults	3	55	60-70% HRmax	0
	HIIT(15)	12	47.3(5.1)	29.7(4.1)	93	Australia	brachial artery	Sedentary overweight/obese adults	3	25	~85% HRmax (30s work / 2.5min recovery)	
	CG(14)	12	51.2(7.0)	28.7(3.6)	NR	Australia	brachial artery	Sedentary overweight/obese adults	0	0	NA	
Cox et al. 2024 [13]	HIIT(11)	8	59.0(8.8)	32.6(5.1)	96.7	Australia	brachial artery	T2D	3	26	aerobic 85-95% HRpeak; resistance RPE≥17	39.1
	AE-RT(15)	8	60.1(7.3)	34.0(6.6)	94.1	Australia	brachial artery	T2D	4	52.5	aerobic 55-69% HRpeak; resistance RPE 11-13	
	CG(15)	8	59.4(10.2)	33.9(6.4)	NR	Australia	brachial artery	T2D	0	0	NA	
Craenenbroeck et al. 2015 [14]	HIIT(100)	12	57.0(8.8)	28.0(4.4)	NR	Belgium	brachial artery	CAD	3	38	90-95% HRpeak (4 min) / 50-70% HRpeak (3 min)	10
	MICT(100)	12	59.9(9.2)	28.5(4.3)	NR	Belgium	brachial artery	CAD	3	37	≥70-75% HRpeak	
Currie et al. 2013 [15]	MICT(11)	12	68(8)	27.3(4.2)	91.7	Canada	brachial artery	CAD	3	40	58% PPO	9.1

	HIIT(11)	12	62(11)	27.9(4.9)	79.2	Canada	brachial artery	CAD	3	20	89% PPO (1 min) / 10% PPO (1 min)	
Davoodi et al. 2022 [16]	HIIT(16)	12	52.5(7.2)	28.98(1.07)	70	Iran	brachial artery	T2D + hypertension	3	18	85-90% HRmax	42.6
	MICT(16)	12	54.9(5.6)	29.32(0.90)	70	Iran	brachial artery	T2D + hypertension	3	42	70% HRmax	
	CG(15)	12	53.7(6.1)	28.92(1.67)	NR	Iran	brachial artery	T2D + hypertension	0	0	NA	
Franklin et al. 2015 [17]	RT(10)	8	30.8(9)	34.2(3)	90	USA	brachial artery	Obesity	2	45	80-90% of 10-RM	100
	CG(8)	8	30.3(5.4)	32.2(6.9)	NR	USA	brachial artery	Obesity	0	0	NA	
Haykowsky et al. 2009 [18]	AE-RT(15)	12	57(10)	27.7	89	Canada	brachial artery	Heart transplant recipients	5	aerobic30-45/ interval10-20/ resistance15-20	aerobic 60-80% VO₂peak; interval 90-100% PPO; resistance 50% 1RM	18.6
	CG(16)	12	59(11)	31.4	NR	Canada	brachial artery	Heart transplant recipients	0	0	NA	
Hermann et al. 2011 [19]	HIIT(14)	8	53(11)	26.3(3.4)	NR	Denmark	brachial artery	Heart transplant recipients	3	60	80-90% VO_2peak (RPE 18-19)	18.5
	CG(13)	8	47(18)	26.1(6.1)	NR	Denmark	brachial artery	Heart transplant recipients	0	0	NA	
Hildreth et al. 2018 [20]	RT(19)	52	66(5)	28.9(3.4)	NR	USA	brachial artery	Apparently healthy older adults	3	50	50-80% 1RM (progressive)	0
	CG(21)	52	67(5)	27.2(3)	NR	USA	brachial artery	Apparently healthy older adults	0	0	NA	
Hovsepian et al. 2021 [21]	HIIT(13)	10	20.23(1.30)	29.7(4.9)	86	Iran	brachial artery	Overweight/obesity	4	16	85-90% HRmax	100
	CG(12)	10	20.69(1.70)	32.1(5.8)	NR	Iran	brachial artery	Overweight/obesity	0	0	NA	
Isaksen et al. 2019 [22]	HIIT(19)	12	66(9)	27.2(3.8)	NR	Norway	brachial artery	Ischemic heart failure	3	16	85% HRmax	6.7

	CG(11)	12	69(9)	27.3(4.2)	NR	Norway	brachial artery	Ischemic heart failure	0	0	NA	
Jang et al. 2023 [23]	MBE(12)	12	41.25(3.31)	27.41(2.45)	99.15	Korea	brachial artery	Obesity	3	50	Main exercise RPE 13-15	100
	CG(12)	12	39.92(2.94)	28.52(3.73)	NR	Korea	brachial artery	Obesity	0	0	NA	
Kitzman et al. 2013 [24]	AE(25)	16	70(7)	32.2(6.7)	88	USA	brachial artery	HFpEF	3	60	40-70% HRR (progressive)	75.9
	CG(30)	16	70(7)	32.0(6.6)	NR	USA	brachial artery	HFpEF	0	0	NA	
Kwon et al. 2011 [25]	AE(13)	12	55.5(8.6)	26.7(2.6)	NR	South Korea	brachial artery	T2D	5	60	4-6 MET (moderate)	100
	RT(12)	12	56.3(6.1)	27.4(2.1)	NR	South Korea	brachial artery	T2D	3	60	40-50% 1RM	
	CG(15)	12	58.9(5.7)	27.0(2.3)	NR	South Korea	brachial artery	T2D	0	0	NA	
Lavrenčič et al. 1999 [26]	AE(14)	12	53(5)	31.9(3.2)	86	Slovenia	brachial artery	Metabolic syndrome	3	50	80% HRmax	0
	CG(15)	12	51(7)	30.0(3.4)	NR	Slovenia	brachial artery	Metabolic syndrome	0	0	NA	
Lee et al. 2019 [27]	HIIT(15)	8	49.1(7.9)	33.1(7.6)	82.3	USA	brachial artery	Breast cancer	3	25	90% PPO (1 min) / 10% PPO (2 min)	100
	CG(15)	8	44.7(11.2)	30.1(7.7)	NR	USA	brachial artery	Breast cancer	0	0	NA	
Mohamed et al. 2021 [28]	AE-RT(20)	20	53.95(5.81)	29.27(2.33)	NR	Egypt	brachial artery	Refractory angina	3	80	aerobic 60-75% HRR; resistance 30-60% 1RM	0
	CG(20)	20	54.30(6.59)	29.91(2.98)	NR	Egypt	brachial artery	Refractory angina	0	0	NA	

Molmen-Hansen et al. 2012 [29]	HIIT(25)	12	52.5(7.4)	26.8(4.1)	NR	Norway	brachial artery	Hypertension	3	38	90-95% HRmax (4 min) / 60-70% HRmax (3 min)	44.3
	MICT(23)	12	53.6(6.5)	27.9(3.2)	NR	Norway	brachial artery	Hypertension	3	47	70% HRmax	
	CG(25)	12	51.3(9.2)	28.8(3.7)	NR	Norway	brachial artery	Hypertension	0	0	NA	
OLSON et al. 2006 [30]	RT(15)	52	38(1)	27.5(0.9)	90	USA	brachial artery	Healthy overweight women	2	50	50-80% 1RM	100
	CG(15)	52	38(2)	27.6(0.7)	NR	USA	brachial artery	Healthy overweight women	0	0	NA	
Orio et al. 2016 [31]	AE(39)	24	25.9(2.7)	26.7(2.8)	NR	Italy	brachial artery	PCOS	3	45	60-70% VO_2max	100
	CG(50)	24	26.0(2.6)	27.0(2.9)	NR	Italy	brachial artery	PCOS	0	0	NA	
Pattyn et al. 2016 [32]	HIIT(70)	12	57.4(8.7)	27.8(4.0)	NR	Belgium	brachial artery	CAD	3	38	85-90% HRpeak (4 min) / 50-70% HRpeak (3 min)	6.7
	MICT(69)	12	59.9(9.2)	28.4(4.2)	NR	Belgium	brachial artery	CAD	3	47	≥70-75% HRpeak	
Peng et al. 2026 [33]	AE(14)	8	60.1(5.5)	25.64(2.7)	NR	China	brachial artery	Apparently healthy older adults	3	45	60-85% HRmax	70.2
	CG(11)	8	62.3(6.9)	25.18(5.09)	NR	China	brachial artery	Apparently healthy older adults	0	0	NA	
Pugh et al. 2014 [34]	AE(13)	16	48(2)	31(2)	92	UK	brachial artery	NAFLD + obesity	3-5	40	30% HRR -60% HRR	47.6
	CG(8)	16	47(2)	30(2)	NR	UK	brachial artery	NAFLD + obesity	0	0	NA	
Rahimianmashhad et al. 2020 [35]	AE(14)	8	30-40	29.73(1.81)	NR	Iran	brachial artery	Obesity	3	40	50-70% HRR	100
	RT(13)	8	30-40	31.27(3.41)	NR	Iran	brachial artery	Obesity	3	45	60-85% 1RM	
Robinson et al. 2016 [36]	AE(10)	8	34(8)	32(5)	NR	USA	brachial artery	Overweight/obesity	3	40	75% HRmax	73.7

Sawyer et al. 2016 [37]	CG(9)	8	28(5)	33(6)	NR	USA	brachial artery	Overweight/obesity	0	0	NA	50
	MICT(9)	8	34.8(7.7)	34.5(3.2)	100	USA	brachial artery	Apparently healthy obesity	3	30	70-75% HRmax	
	HIIT(9)	8	35.6(8.9)	37.4(6.2)	100	USA	brachial artery	Apparently healthy obesity	3	19	90-95% HRmax (1 min) / low-intensity recovery (1 min)	
Silva et al. 2019 [38]	AE(14)	17.14	50(17.2)	25.7(3.58)	NR	Brazil	brachial artery	CKD on hemodialysis	3	30	65-75% HRmax	50
	CG(14)	17.14	58(15.0)	26.7(4.60)	NR	Brazil	brachial artery	CKD on hemodialysis	0	0	NA	
Sixt et al. 2010 [39]	AE-RT(14)	24	62.3(6.2)	29.8(4.0)	NR	Germany	brachial artery	CAD + prediabetes	(7 home + 2 supervised)	120	75% HRmax	21.4
	CG(12)	24	62.3(6.5)	31.3(3.9)	NR	Germany	brachial artery	CAD + prediabetes	0	0	NA	
Turri- Silva et al. 2021 [40]	HIIT(8)	12	60.9(9.7)	29.4(5.2)	93.5	Brazil	brachial artery	Heart failure	3	16	High intensity (above VT2)	27.27
	RT(6)	12	55.0(10.9)	32.9(6.5)	97.2	Brazil	brachial artery	Heart failure	3	25	60-80% 1RM	
	CG(8)	12	56.0(9.7)	28.6(4.5)	NR	Brazil	brachial artery	Heart failure	0	0	NA	
Vaccarino et al. 2013 [41]	MBE(33)	52	51.5(7.5)	38.2(11.2)	50	USA	brachial artery	Metabolic syndrome risk	14	20	Very low intensity	78.9
	CG(35)	52	52.1(7.6)	36.7(5.6)	NR	USA	brachial artery	Metabolic syndrome risk	14	20	Very low intensity	
Valentino et al. 2022 [42]	AE(9)	4	61(9)	29.8(3.3)	NR	Canada	brachial artery	CAD	3	45	60-80% HRR	11.1
	HIIT(9)	4	61(9)	29.7(4.1)	NR	Canada	brachial artery	CAD	3	35	RPE 14-15 (vigorous)	
Vinet et al. 2018 [43]	RT(21)	24	62.7(4.2)	32.3(4.4)	NR	France	brachial artery	Metabolic syndrome	3	60	70% 1RM	58.1
	AE(17)	24	58.2(3.8)	32.8(0.7)	NR	France	brachial artery	Metabolic syndrome	3	60	70% VO_2peak	
	AE-RT(24)	24	59.2(5.1)	33.7(3.6)	NR	France	brachial artery	Metabolic syndrome	3	60	30% 1RM + 30% VO_2peak	

Vona et al. 2004 [44]	AE(28)	12	56(6)	26.5(2)	88	Italy	brachial artery	Post-AMI	3	40	75% peak HR	23.1
	CG(24)	12	57(8)	25.7(6)	NR	Italy	brachial artery	Post-AMI	0	0	NA	
Vona et al. 2009 [45]	AE(52)	4	56(6)	26.5(2.4)	NR	Italy	brachial artery	Post-AMI	4	40	75% peak HR	32.1
	RT(54)	4	57(8)	25.9(2.8)	NR	Italy	brachial artery	Post-AMI	4	45	60% 1RM	
	AE-RT(53)	4	55(9)	26.3(2.5)	NR	Italy	brachial artery	Post-AMI	4	aerobic 40/ resistance ~45	aerobic 75% peak HR; resistance 60% 1RM	
	CG(50)	4	58(7)	25.7(3.4)	NR	Italy	brachial artery	Post-AMI	0	0	NA	
Westhoff et al. 2007 [46]	AE(25)	12	67.8(4.7)	27.7(4.4)	100	Germany	brachial artery	Hypertension	3	33	Lactate threshold (~2.0 mmol/L)	50
	CG(27)	12	68.9(5.2)	30.1(4.4)	NR	Germany	brachial artery	Hypertension	0	0	NA	
Yu et al. 2025 [47]	HIIT(21)	8	22.0(2)	27.1(1.8)	100	China	brachial artery	Overweight/obesity	3	6	80-85% HRmax (week 1), 85-95% HRmax (weeks 2-8)	21.4
	MICT(20)	8	23.8(5.4)	27.4(2.0)	100	China	brachial artery	Overweight/obesity	3	30	70-75% HRmax	
	CG(15)	8	24.9(7.1)	27.4(2.0)	NR	China	brachial artery	Overweight/obesity	0	0	NA	

Note: Values are presented as mean (SD) unless otherwise specified. BMI is reported in kg/m². Exercise frequency refers to sessions per week, and session duration refers to minutes per session. For combined exercise interventions, frequency, duration, and intensity are presented by exercise component when reported. The brachial artery was the assessment site for FMD in all included studies. Control groups were coded as 0 MET-min/week for dose calculation. NR indicates not reported; NA, not applicable.

Abbreviations: AE, aerobic exercise; AE-RT, combined aerobic and resistance training; AMI, acute myocardial infarction; BMI, body mass index; CABG, coronary artery bypass grafting; CAD, coronary artery disease; CG, control group; CKD, chronic kidney disease; FMD, flow-mediated dilation; HIIT, high-intensity interval training; HRmax, maximal heart rate; HRpeak, peak heart rate; HRR, heart rate reserve; MBE, mind-body exercise; MICT, moderate-intensity continuous training; NAFLD, non-alcoholic fatty liver disease; NR, not reported; PAD, peripheral artery

disease; PCI, percutaneous coronary intervention; PCOS, polycystic ovary syndrome; PPO, peak power output; RM, repetition maximum; RPE, rating of perceived exertion; RT, resistance training; T2D, type 2 diabetes; VT, ventilatory threshold; VO_2peak, peak oxygen uptake.

4. Supplementary methods

- **Agent:** The agent level.
- **METs_min:** The energy expenditure per min in the study.
- **Min_session：** The duration of a single exercise intervention.
- **Frequency:** The number of times participants engaged in physical activity (times/week).
- **Exact_dose:** The exact estimated METs accumulated per week by participants (MET-min/week).
- **Dose:** The dose group by approximation (MET-min/week).
- **CG:** Control group

Table S3. Dose categorization table

Study ID	Agent	METs_min	Min_session	Frequency	Prescribed dose	Average adherence rate (%)	Exact_dose	Dose
1	AE	7	30	3	630	80	504	500
1	CG	0	0	0	0	0	0	0
2	RT	3.5	20	3	210	100	210	250
2	CG	0	0	0	0	0	0	0
3	AE-RT	AE: 5.5	20	3	330+210	92	496.8	500
3		RT: 3.5	20	3				

3	CG	0	0	0	0	0	0	0
4	HIIT	7	20	3	420	91	382.2	400
4	CG	0	0	0	0	0	0	0
5	MBE(Yoga)	3.3	30	3	297	90	267.3	250
5	CG	0	0	0	0	0	0	0
6	MICT	7.3	30	3	657	90	591.3	600
6	CG	0	0	0	0	0	0	0

5.Risk of bias

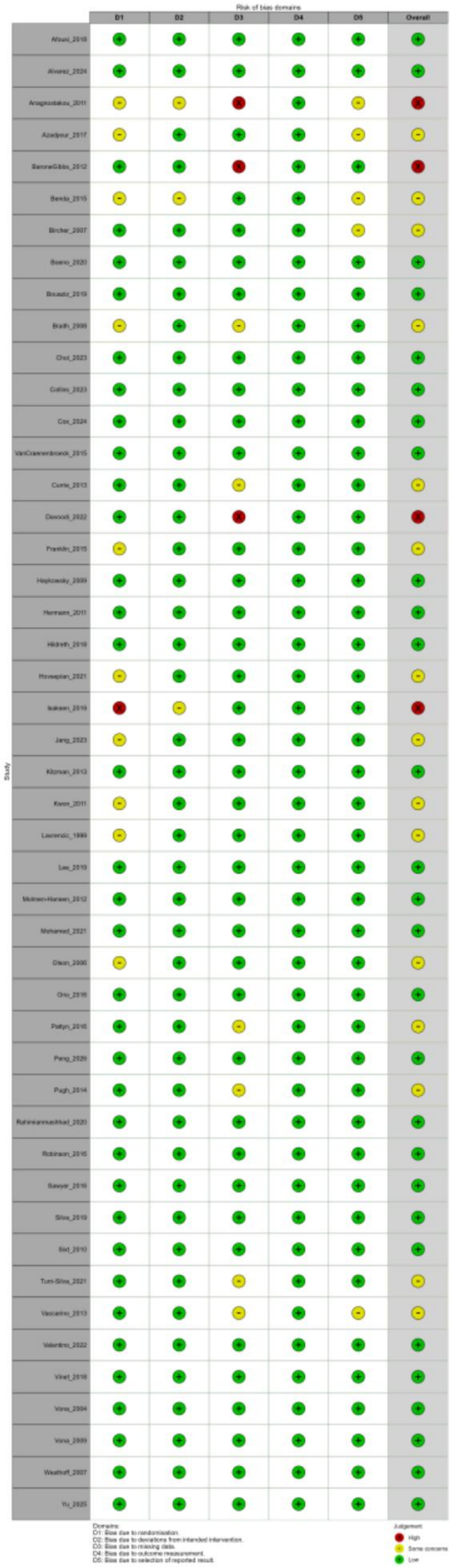

Figure S1. Detailed risk-of-bias assessment.

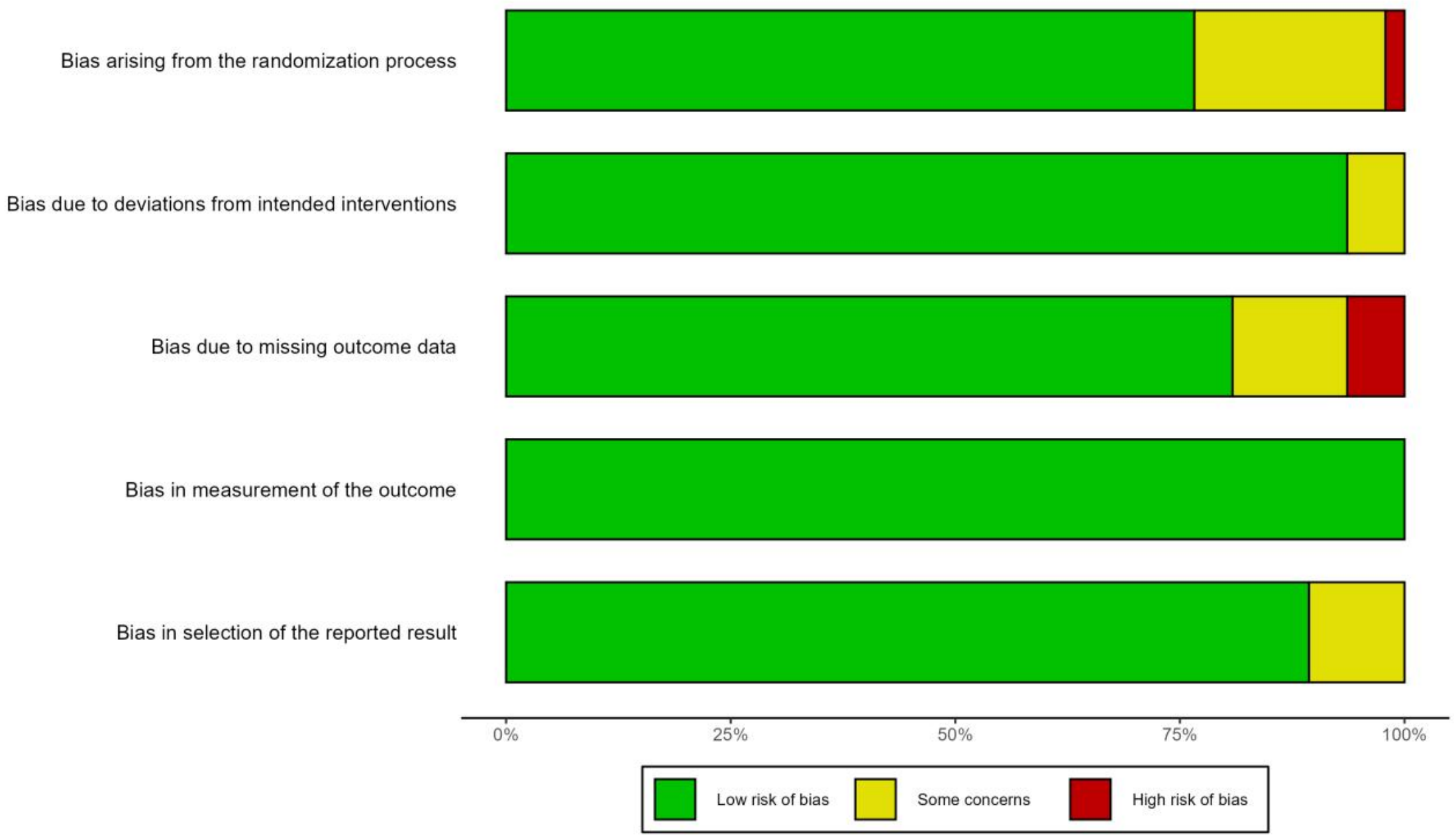

Figure S2. The summary of risk of bias.

6. Dose-response network meta-analyses

6.1 Connectivity

Connectivity is a fundamental assumption in network meta-analysis (NMA). Insufficient connectivity, often resulting from limited direct comparisons, can reduce statistical power and bias results. In our study, connectivity was assessed across all dose and agent levels for each outcome (Figure S3-S4), with no evidence of network disconnection observed.

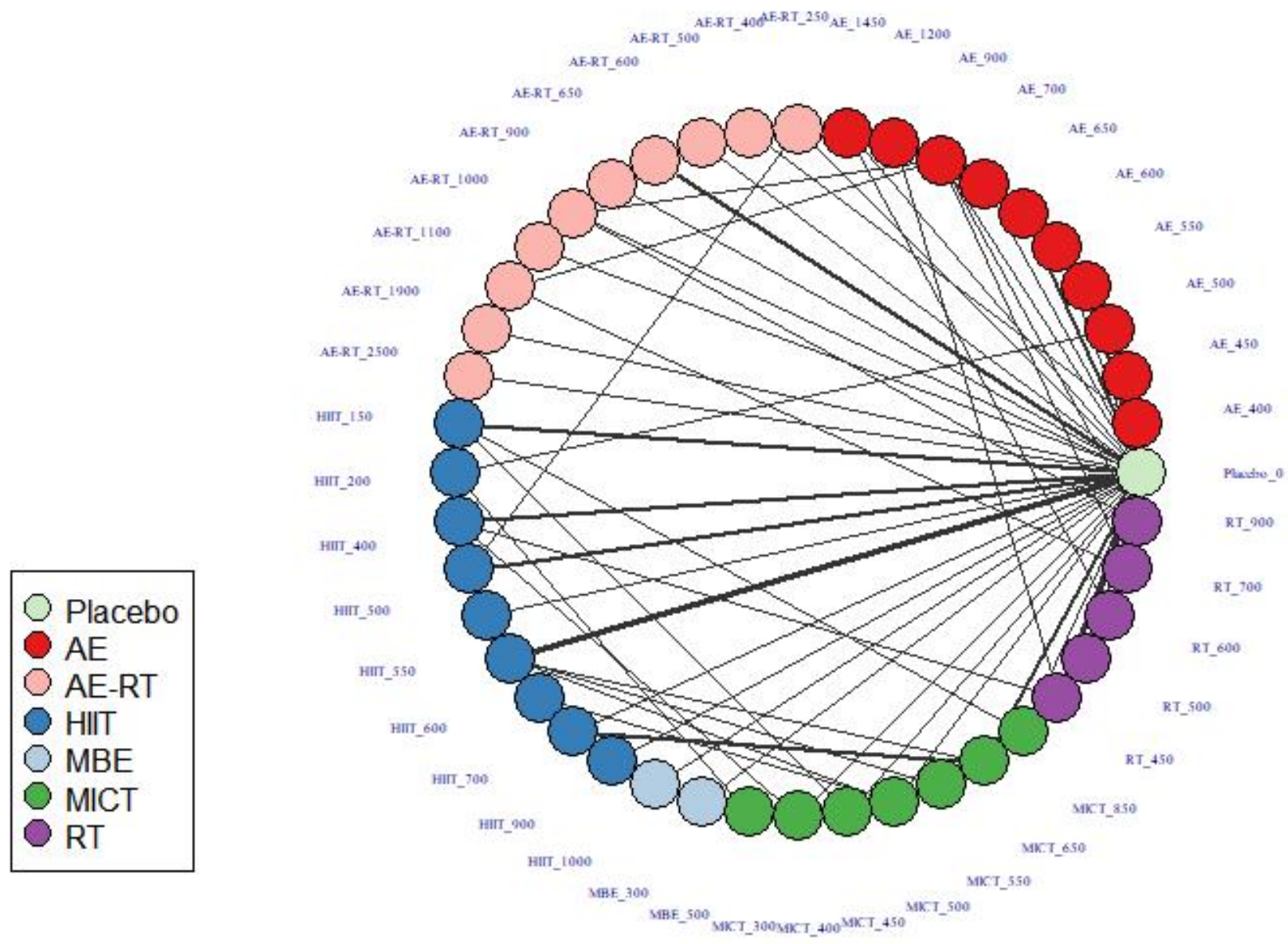

Figure S3. Dose-level network (Flow-Mediated Dilation).

Line thickness represents the number of studies available for comparison. The first value represents the specific intervention, and the second value corresponds to the dose (METs - min/week).

AE: Aerobic Exercise; AE-RT: Combined Aerobic and Resistance Training; RT: Resistance Training;MBE:Mind-body exercise;HIIT: High-Intensity Interval Training;MICT:Moderate-Intensity Continuous Training;CG: Control group

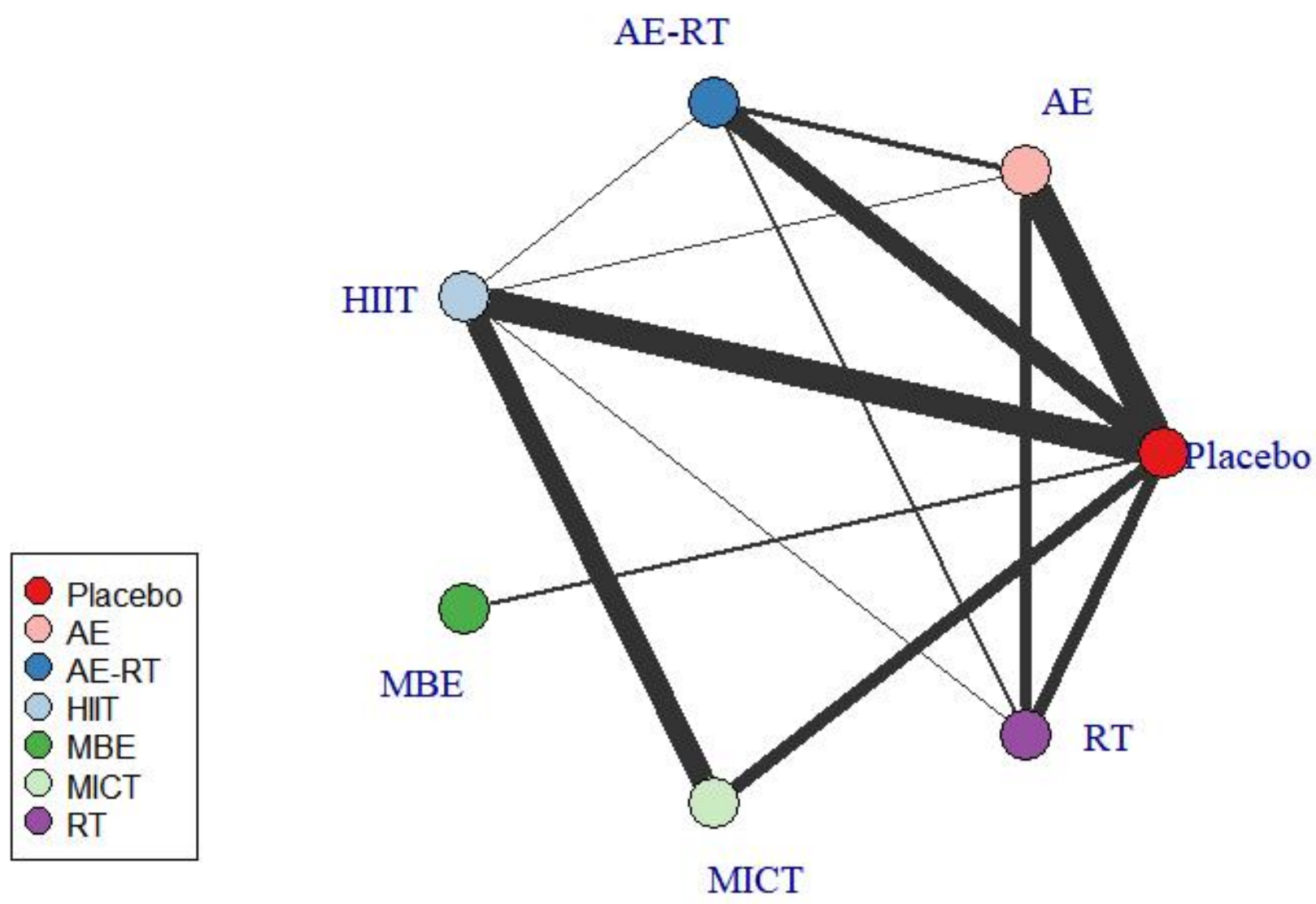

Figure S4. Agent-level network (Flow-Mediated Dilation).

Line thickness represents the number of studies available for comparison.

AE: Aerobic Exercise; AE-RT: Combined Aerobic and Resistance Training; RT: Resistance Training;MBE:Mind-body exercise;HIIT: High-Intensity Interval Training;MICT:Moderate-Intensity Continuous Training;CG: Control group

6.2 Consistency

We performed a consistency analysis of the data by comparing the consistency of the network i.e., network effect size with the unrelated mean effects UME model i.e., pairwise effect sizes. In practice, we checked whether the bias, the number of estimated parameters in the network, and the Deviance Information Criterion DIC metric were similar for both models, indicating a good fit. The comparison of these parameters showed a good agreement between the different models Table S5.

Table S4. Flow-Mediated Dilation Comparison of consistency and UME model fit.

Model	PD	Deviance	Residual deviance	DIC	SD
Consistent	93.0	233.081	109.972	326.6	2.061
UME	94.3	231.642	108.533	325.9	2.134

Note: UME = unrelated mean effects model; DIC = deviance information criterion; PD = effective number of parameters; SD = standard deviation

6.3 Inconsistency assessment

Network meta-analyses (NMAs) rely on the assumption of transitivity, which means that estimates of treatment effects derived from direct and indirect comparisons are consistent, accounting for the usual variability expected under a random-effects model. This assumption is analogous to the concept of heterogeneity in a standard meta-analysis. Following previous

recommendations, we evaluated potential anomalies at a more granular level, specifically at the treatment level. The span of the network was assessed using the MBNMA node-splitting method, which separates the contribution of each treatment contrast into direct and indirect evidence and compares them. Consistent effects between these sources indicate good transitivity. Results of this assessment are presented in Table S5.

Table S5. Flow-Mediated Dilation Node-splitting analysis of consistency.

Comparison	p-value	Median	2.5%	97.5%
MICT_650 vs HIIT_900	**0.619**			
-> direct		-1.859	-4.633	0.974
-> indirect		-1.035	-2.579	0.483
-> MBNMA		-1.175	-2.564	0.106
MICT_500 vs HIIT_700	**0.063**			
-> direct		-5.934	-10.232	-1.655
-> indirect		-0.686	-2.018	0.547
-> MBNMA		-1.166	-2.475	0.091
MICT_550 vs HIIT_600	**0.498**			
-> direct		-0.251	-4.128	3.624
-> indirect		-1.245	-2.645	0.165
-> MBNMA		-1.091	-2.407	0.164
MICT_400 vs HIIT_400	**0.418**			
-> direct		-0.029	-4.741	4.447
-> indirect		-1.056	-2.326	0.208
-> MBNMA		-0.997	-2.240	0.180
MICT_300 vs HIIT_200	**0.452**			
-> direct		0.201	-4.062	4.457
-> indirect		-0.764	-2.077	0.495
-> MBNMA		-0.694	-1.931	0.507
RT_450 vs AE_1200	**0.545**			
-> direct		-0.335	-4.482	3.982
-> indirect		-0.604	-2.212	0.833
-> MBNMA		-0.573	-2.026	0.849
RT_700 vs AE_900	**0.666**			
-> direct		-0.956	-4.191	2.301
-> indirect		-0.379	-1.993	1.228
-> MBNMA		-0.358	-1.832	1.082

AE-RT_1100 vs AE_900	**0.503**			
-> direct		-0.293	-3.849	3.408
-> indirect		1.164	-0.545	2.908
-> MBNMA		0.901	-0.585	2.424
HIIT_200 vs AE_500	**0.394**			
-> direct		-1.426	-6.345	3.507
-> indirect		0.450	-1.144	1.950
-> MBNMA		0.515	-1.069	1.915
RT_500 vs AE_400	**0.082**			
-> direct		-6.189	-12.604	-0.170
-> indirect		0.130	-1.351	1.572
-> MBNMA		-0.186	-1.574	1.208
AE-RT_400 vs AE_400	**0.154**			
-> direct		4.779	0.003	9.514
-> indirect		0.404	-1.032	1.984
-> MBNMA		0.759	-0.623	2.167
RT_900 vs Placebo_0	**0.226**			
-> direct		5.245	2.212	8.240
-> indirect		2.490	0.932	4.087
-> MBNMA		2.806	1.388	4.229
RT_600 vs Placebo_0	**0.453**			
-> direct		1.373	-2.072	4.952
-> indirect		2.886	1.435	4.280
-> MBNMA		2.697	1.332	4.028
RT_450 vs Placebo_0	**0.728**			
-> direct		2.099	-0.254	4.392
-> indirect		2.675	1.073	4.289
-> MBNMA		2.592	1.270	3.892
MICT_850 vs Placebo_0	**0.495**			
-> direct		2.160	-2.673	7.077
-> indirect		1.975	0.345	3.537
-> MBNMA		1.943	0.494	3.467
MICT_500 vs Placebo_0	**0.631**			
-> direct		1.771	-1.621	5.267
-> indirect		1.826	0.304	3.279
-> MBNMA		1.843	0.466	3.252

MICT_450 vs Placebo_0	**0.482**			
-> direct		2.325	-2.512	7.153
-> indirect		1.788	0.293	3.248
-> MBNMA		1.818	0.463	3.205
MBE_500 vs Placebo_0	**0.609**			
-> direct		4.575	-1.918	11.137
-> indirect		1.937	-2.283	6.249
-> MBNMA		2.596	-0.866	6.254
MBE_300 vs Placebo_0	**0.590**			
-> direct		1.701	-2.245	5.587
-> indirect		4.265	-2.097	10.326
-> MBNMA		2.377	-0.795	5.781
HIIT_1000 vs Placebo_0	**0.543**			
-> direct		3.405	-0.409	7.160
-> indirect		3.079	1.715	4.451
-> MBNMA		3.107	1.863	4.493
HIIT_600 vs Placebo_0	**0.697**			
-> direct		3.340	0.943	5.865
-> indirect		2.848	1.431	4.225
-> MBNMA		2.962	1.796	4.190
HIIT_550 vs Placebo_0	**0.342**			
-> direct		1.206	-2.737	4.888
-> indirect		3.120	1.854	4.408
-> MBNMA		2.930	1.780	4.152
HIIT_500 vs Placebo_0	**0.414**			
-> direct		1.577	-1.182	4.356
-> indirect		2.994	1.799	4.295
-> MBNMA		2.893	1.752	4.091
HIIT_150 vs Placebo_0	**0.304**			
-> direct		4.140	0.091	8.052
-> indirect		2.053	1.142	3.235
-> MBNMA		2.180	1.216	3.452
AE-RT_2500 vs Placebo_0	**0.419**			
-> direct		2.531	-1.715	6.860
-> indirect		4.410	2.830	6.126

-> MBNMA		4.161	2.784	5.701
AE-RT_1900 vs Placebo_0	**0.399**			
-> direct		5.583	0.122	11.091
-> indirect		4.044	2.588	5.604
-> MBNMA		4.119	2.768	5.610
AE-RT_1000 vs Placebo_0	**0.065**			
-> direct		7.736	4.446	11.146
-> indirect		3.356	2.020	4.765
-> MBNMA		3.973	2.683	5.342
AE-RT_900 vs Placebo_0	**0.390**			
-> direct		5.618	2.131	8.903
-> indirect		3.782	2.379	5.251
-> MBNMA		3.941	2.667	5.280
AE-RT_650 vs Placebo_0	**0.082**			
-> direct		0.038	-3.629	3.653
-> indirect		4.324	3.016	5.592
-> MBNMA		3.821	2.594	5.112
AE-RT_600 vs Placebo_0	**0.118**			
-> direct		8.248	3.288	13.513
-> indirect		3.489	2.134	4.822
-> MBNMA		3.782	2.572	5.077
AE-RT_500 vs Placebo_0	**0.233**			
-> direct		0.572	-4.711	5.930
-> indirect		3.881	2.594	5.196
-> MBNMA		3.697	2.513	4.968
AE-RT_250 vs Placebo_0	**0.243**			
-> direct		0.816	-2.908	4.786
-> indirect		3.544	2.263	5.033
-> MBNMA		3.237	2.107	4.588
AE_1450 vs Placebo_0	**0.484**			
-> direct		2.726	-1.288	6.708
-> indirect		3.223	2.035	4.480
-> MBNMA		3.216	2.078	4.406
AE_900 vs Placebo_0	**0.361**			
-> direct		4.646	0.780	8.506

|-> indirect		2.957	1.827	4.166
|-> MBNMA		3.112	2.007	4.227
AE_700 vs Placebo_0	**0.449**			
|-> direct		3.225	-0.859	7.214
|-> indirect		3.018	1.900	4.098
|-> MBNMA		3.035	1.945	4.114
AE_650 vs Placebo_0	**0.358**			
|-> direct		4.420	1.568	7.399
|-> indirect		2.794	1.694	3.841
|-> MBNMA		3.008	1.926	4.078
AE_600 vs Placebo_0	**0.433**			
|-> direct		3.136	-0.921	7.160
|-> indirect		2.959	1.898	4.009
|-> MBNMA		2.980	1.915	4.037
AE_550 vs Placebo_0	**0.084**			
|-> direct		-0.106	-3.277	2.856
|-> indirect		3.318	2.245	4.386
|-> MBNMA		2.943	1.886	3.998
AE_500 vs Placebo_0	**0.553**			
|-> direct		2.381	-0.264	4.987
|-> indirect		2.982	1.881	4.125
|-> MBNMA		2.904	1.868	3.944
AE_450 vs Placebo_0	**0.459**			
|-> direct		2.930	-0.777	6.728
|-> indirect		2.846	1.819	3.892
|-> MBNMA		2.857	1.836	3.885

Note: The first value represents the specific intervention, and the second value corresponds to the dose (MET-min/week).

AE: Aerobic Exercise; AE-RT: Combined Aerobic and Resistance Training; RT: Resistance Training;MBE:Mind-body exercise;HIIT: High-Intensity Interval Training;MICT:Moderate-Intensity Continuous Training;CG: Control group

6.4 Model selection

For model selection, we adopted a quadratic function model. In accordance with the default settings in MBNMAdose, we applied vague (non-informative) prior distributions to minimize bias and allow the data to drive the results, thereby enabling the model to adapt flexibly to the underlying data structure. This approach is designed to capture a specific form of non-linear dose–response relationship and to identify an optimal dose under the assumption of a quadratic association. Below, we present a comparison of parameter estimates across different

models (Table S9). In general, lower DIC indicate a better model fit. As the quadratic function model exhibited the lowest DIC value, we selected it for our analysis.

Table S6. Models fit comparison (Flow-Mediated Dilation)

Model	DIC	SD	Deviance	Residual deviance	pD
Emax (random treatment effects)	332.2	1.819	230.473	107.364	91.2
Restricted cubic spline (random treatment effects; 3 knots)	326.9	2.019	231.258	108.149	95.8
Log Linear (random treatment effect)	324.8	1.791	230.665	107.556	90.4
Non-parametric monotonically up (random treatment effects)	326.6	2.009	230.820	107.711	95.9
Exponential dose-response function (random treatment effects)	334.0	1.392	230.392	107.282	103.5
Quadratic function (random treatment effects)	322.6	1.739	231.888	108.779	90.5
Spline dose-response functions (random treatment effects)	329.0	1.922	232.377	109.268	96.6
Fractional polynomial dose-response function	334.1	1.470	230.081	106.972	103.7

(random treatment effects)

Note: DIC: deviance information criterion; pD: effective number of parameters; SD: standard deviation.

We also validated the fit of the quadratic function model to the data. Figure S5 presents the results of the “Split” network meta-analysis at different doses of various exercise modalities on brachial flow-mediated dilation. This step aimed to explore the dose–response pattern and to assess which functional form best fits the data, thereby informing the selection of a model for the subsequent MBNMA. The model that best matches the pattern shown in Figure S5 should be selected accordingly.

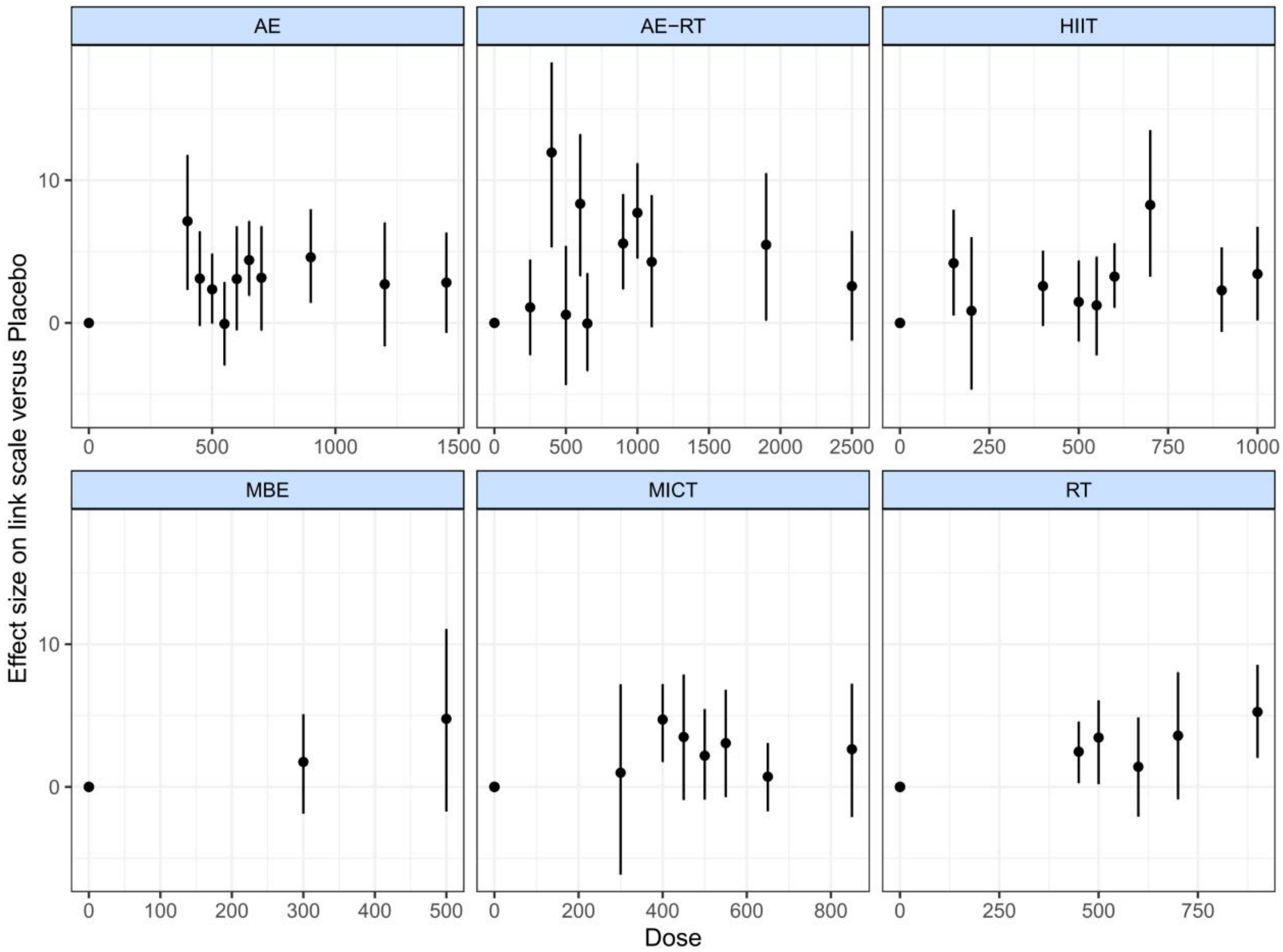

Figure S5. "Split" network meta-analysis of different exercise interventions on flow-mediated dilation.

AE: Aerobic Exercise; AE-RT: Combined Aerobic and Resistance Training; RT: Resistance Training;MBE:Mind-body exercise;HIIT: High-Intensity Interval Training;MICT:Moderate-Intensity Continuous Training;CG: Control group

We observed that the trend of the model curve is generally consistent with the data points from the "Split" network meta-analysis, suggesting a good fit between the model and the data.

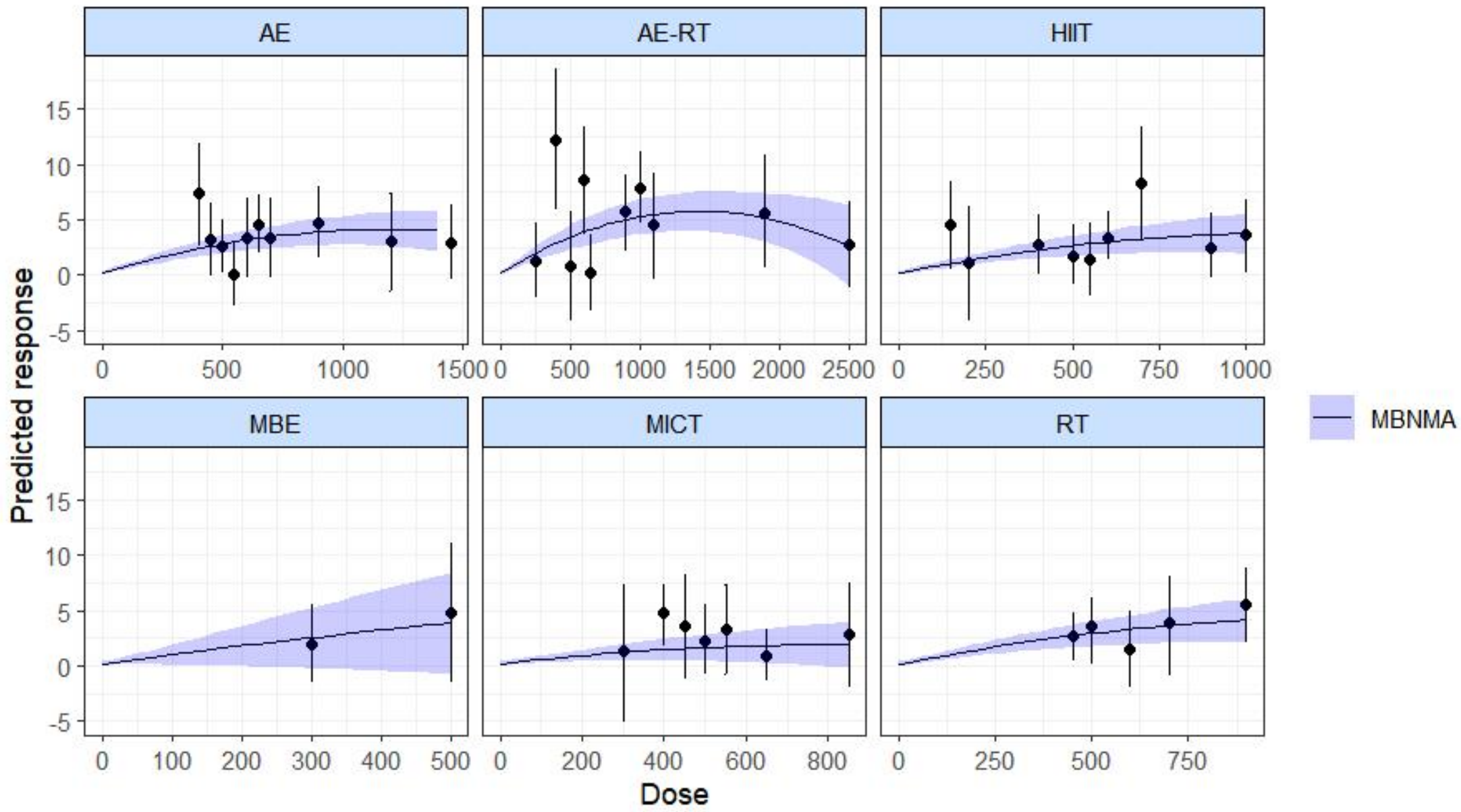

Figure S6. Dose–response relationship between different exercise (METs-min/week) and flow-mediated dilation (quadratic function).

The black dots represent the pooled effect size from the meta-analysis at that dose, while the blue region represents the 95% CrI range.

6.5 Subgroup analysis

6.5.1 Baseline endothelial function (brachial FMD ≤6.5%)

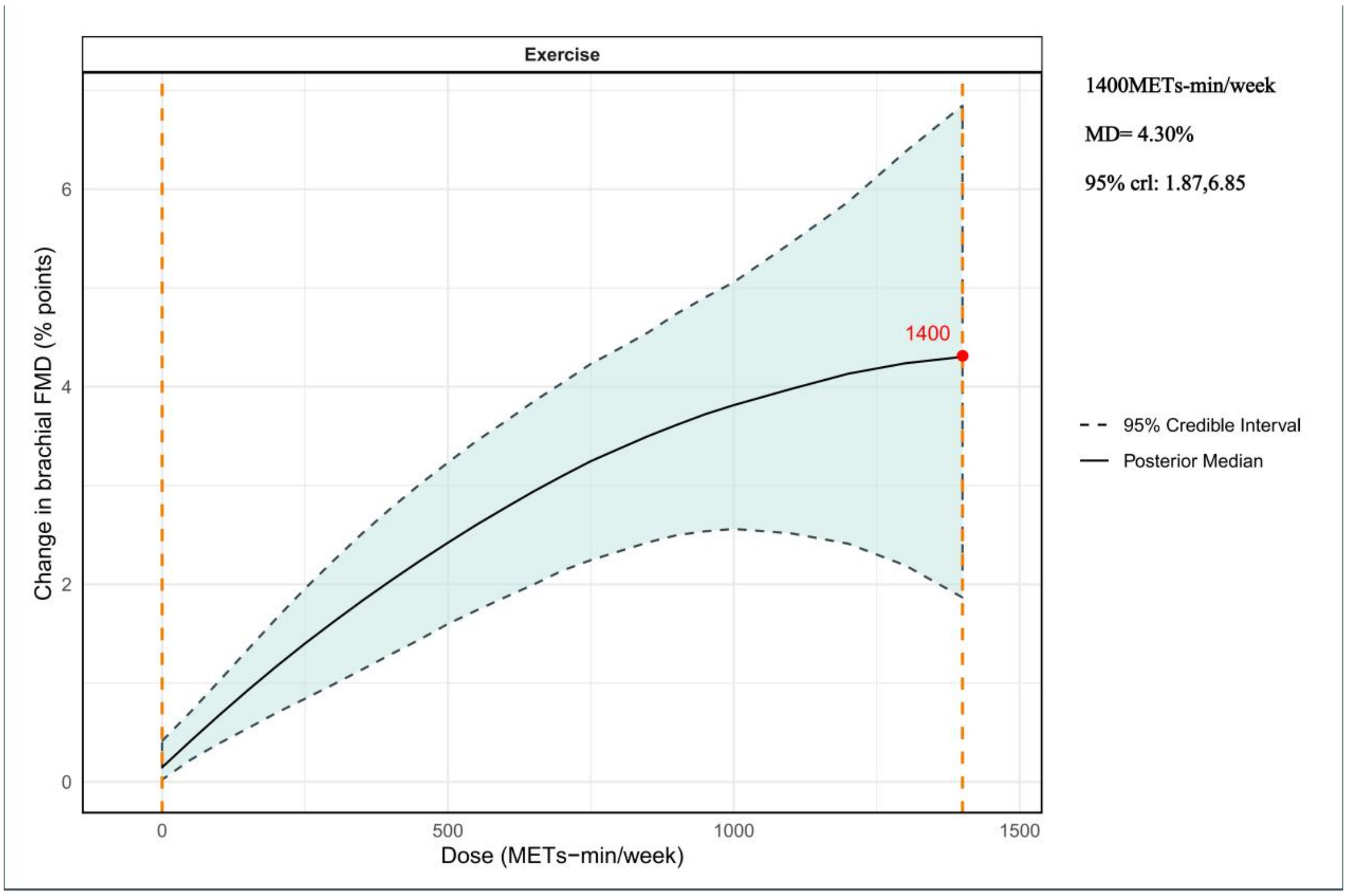

Figure S7. Dose-response relationship between exercise and brachial flow-mediated dilation among participants with baseline brachial FMD ≤6.5%.

The orange dashed lines indicate the modeled dose range from ～1400 METs-min/week. The solid line represents the posterior median, and the dashed curves represent the 95% credible interval. The light green shaded area shows

the uncertainty band around the dose-response curve. The red dot marks the dose associated with the largest estimated improvement in brachial FMD within the modeled range, 1400 METs-min/week.

6.5.2 Baseline endothelial function (brachial FMD>6.5%)

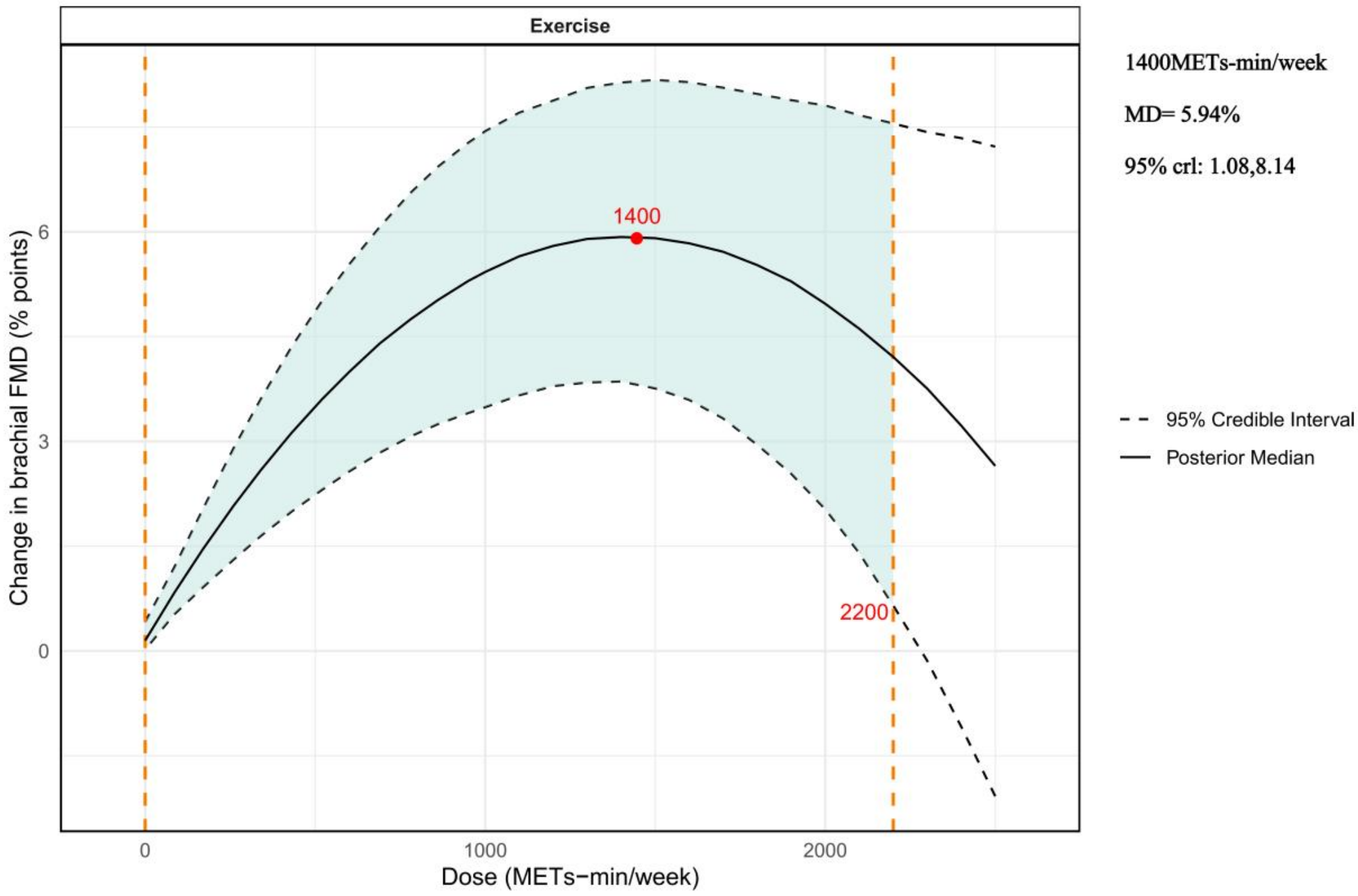

Figure S8. Dose-response relationship between exercise and brachial flow-mediated dilation among participants with baseline brachial FMD＞6.5%.

The orange dashed lines indicate the modeled dose range from ～2200 METs-min/week. The solid line represents the posterior median, and the dashed curves represent the 95% credible interval. The light green shaded area shows the uncertainty band around the dose-response curve. The red dot marks the dose associated with the largest estimated improvement in brachial FMD within the modeled range, 1400 METs-min/week.

6.5.3 Baseline BMI Category (25.0-29.9kg/m²)

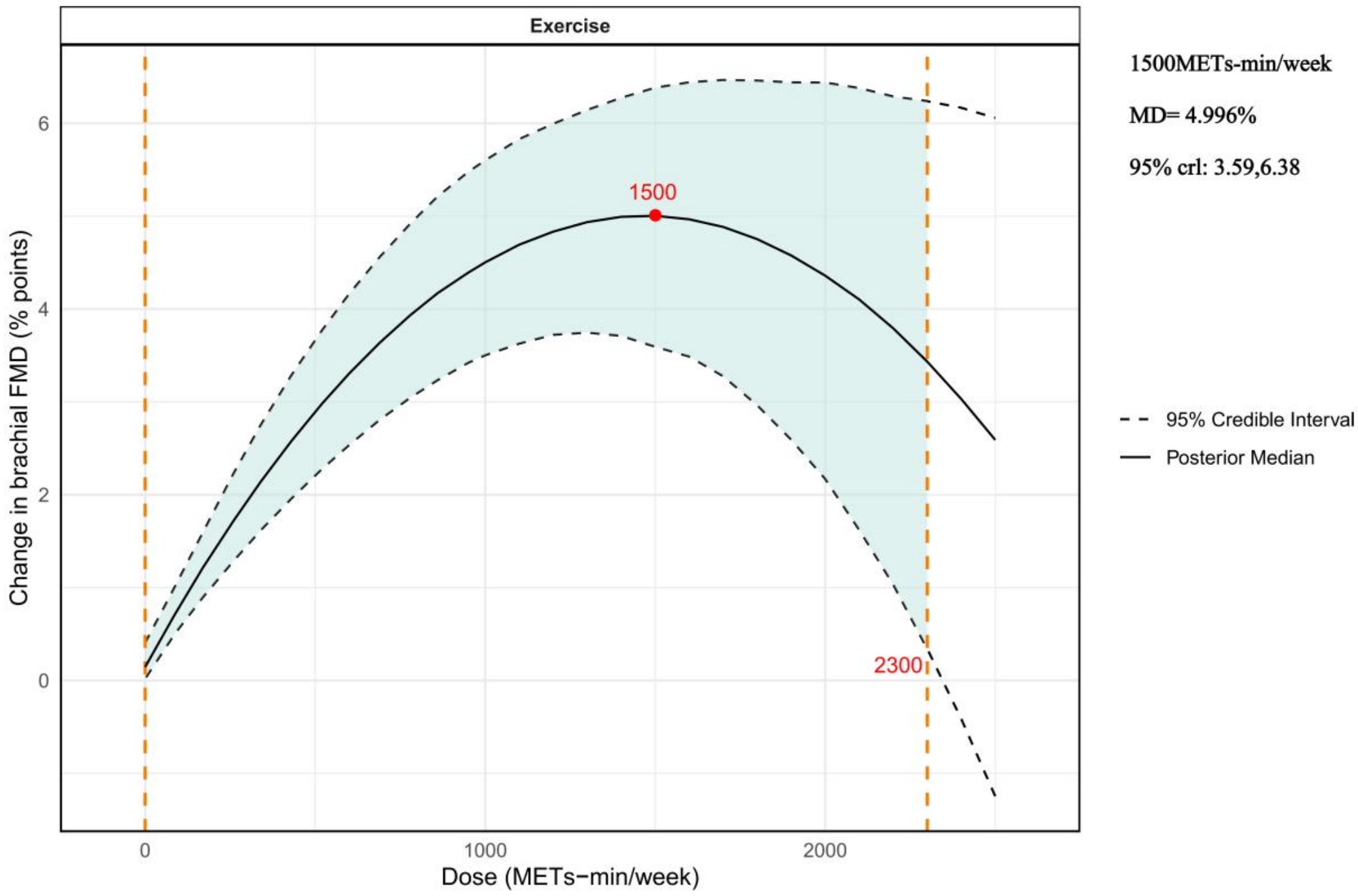

Figure S9. Dose-response relationship between exercise and brachial flow-mediated dilation among participants with baseline BMI 25.0-29.9 kg/m²

The orange dashed lines indicate the modeled dose range from ～2300 METs-min/week. The solid line represents the posterior median, and the dashed curves represent the 95% credible interval. The light green shaded area shows the uncertainty band around the dose-response curve. The red dot marks the dose associated with the largest estimated improvement in brachial FMD within the modeled range, 1500 METs-min/week.

6.5.4 Baseline BMI Category (≥30.0 kg/m²)

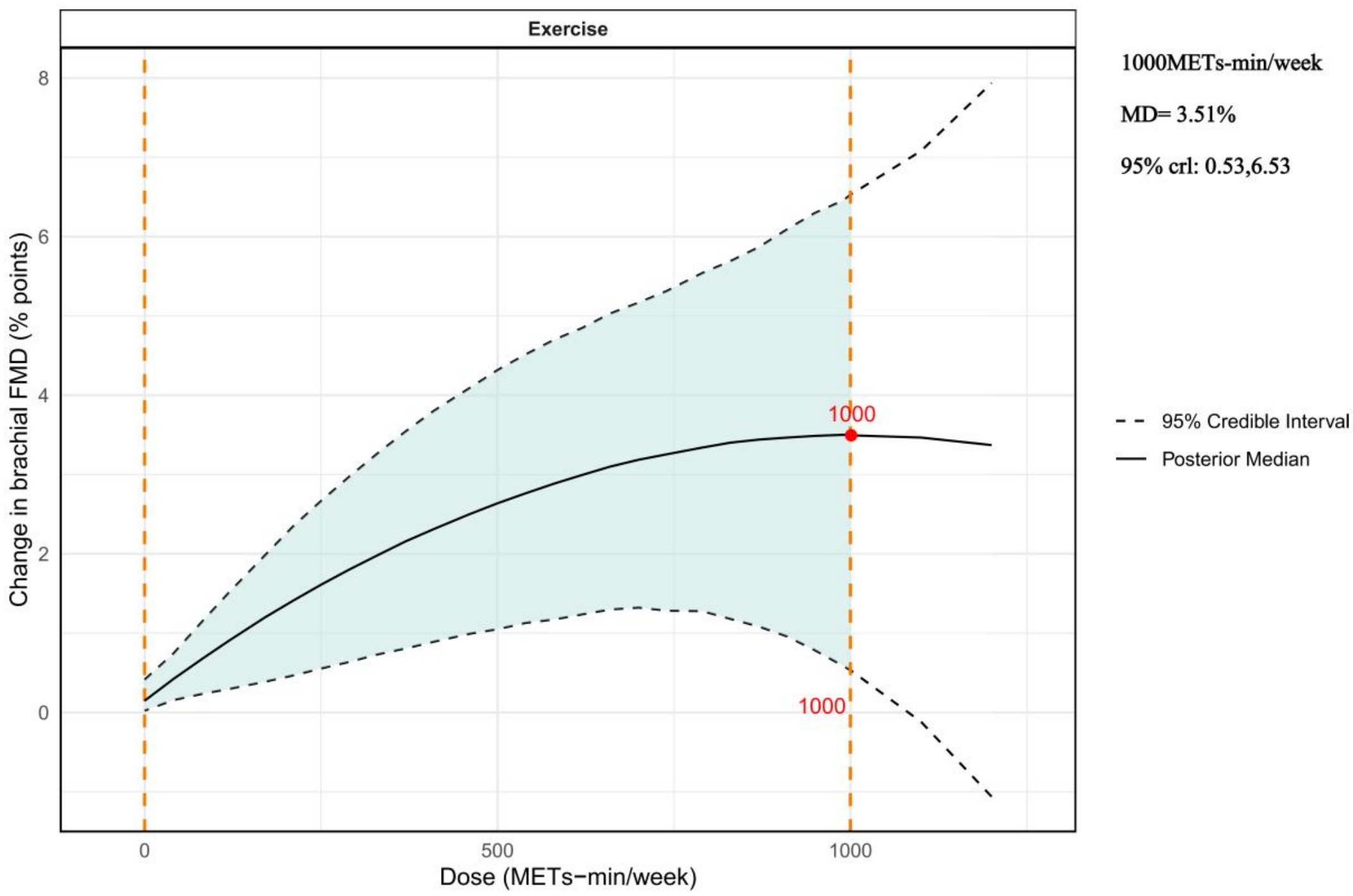

Figure S10. Dose-response relationship between exercise and brachial flow-mediated dilation among participants with baseline BMI ≥30 kg/m²

The orange dashed lines indicate the modeled dose range from ～1000 METs-min/week. The solid line represents the posterior median, and the dashed curves represent the 95% credible interval. The light green shaded area shows the uncertainty band around the dose-response curve. The red dot marks the dose associated with the largest estimated improvement in brachial FMD within the modeled range, 1000 METs-min/week.

6.5.5 Population Status (Apparently Healthy)

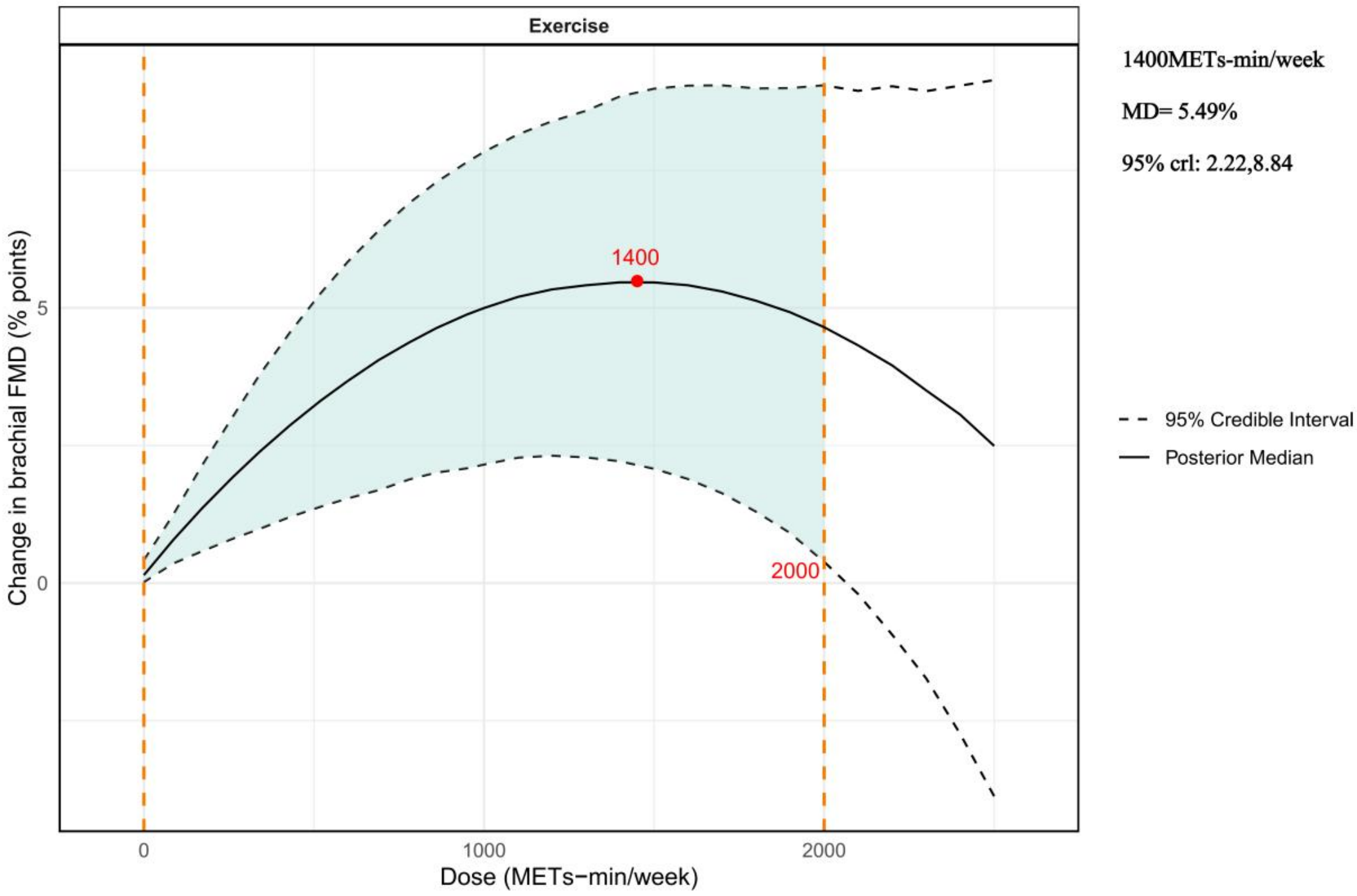

Figure S11. Dose-response relationship between exercise and brachial flow-mediated dilation among apparently healthy adults with overweight or obesity.

The orange dashed lines indicate the modeled dose range from ～2000 METs-min/week. The solid line represents the posterior median, and the dashed curves represent the 95% credible interval. The light green shaded area shows the uncertainty band around the dose-response curve. The red dot marks the dose associated with the largest estimated improvement in brachial FMD within the modeled range, 1400 METs-min/week.

6.5.6 Population Status (Cardiometabolic Disease)

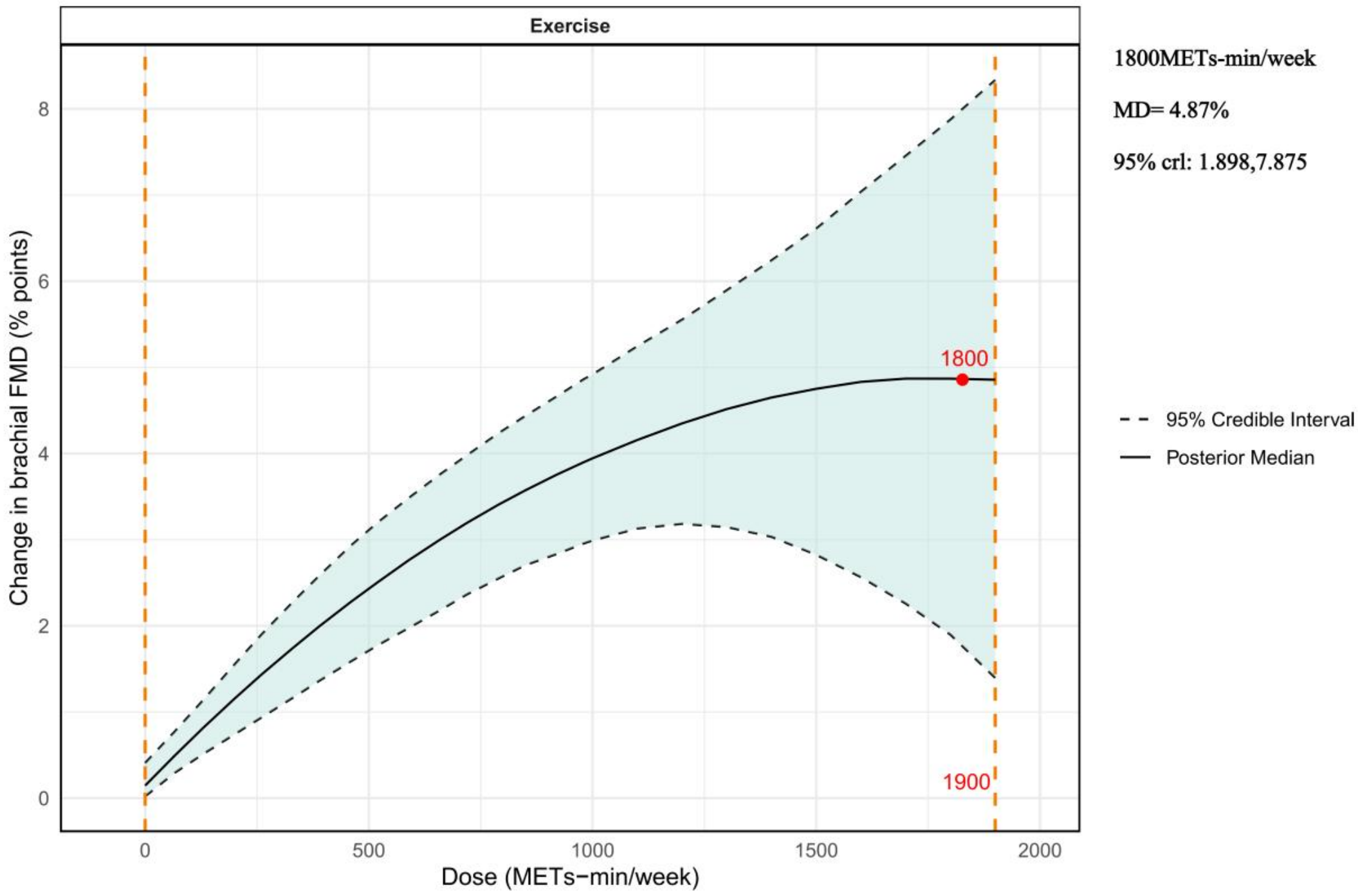

Figure S12. Dose-response relationship between exercise and brachial flow-mediated dilation among adults with overweight or obesity and cardiometabolic disease.

The orange dashed lines indicate the modeled dose range from ～1900 METs-min/week. The solid line represents the posterior median, and the dashed curves represent the 95% credible interval. The light green shaded area shows the uncertainty band around the dose-response curve. The red dot marks the dose associated with the largest estimated improvement in brachial FMD within the modeled range, 1800 METs-min/week.

6.5.7 Intervention Duration (4-8 Weeks)

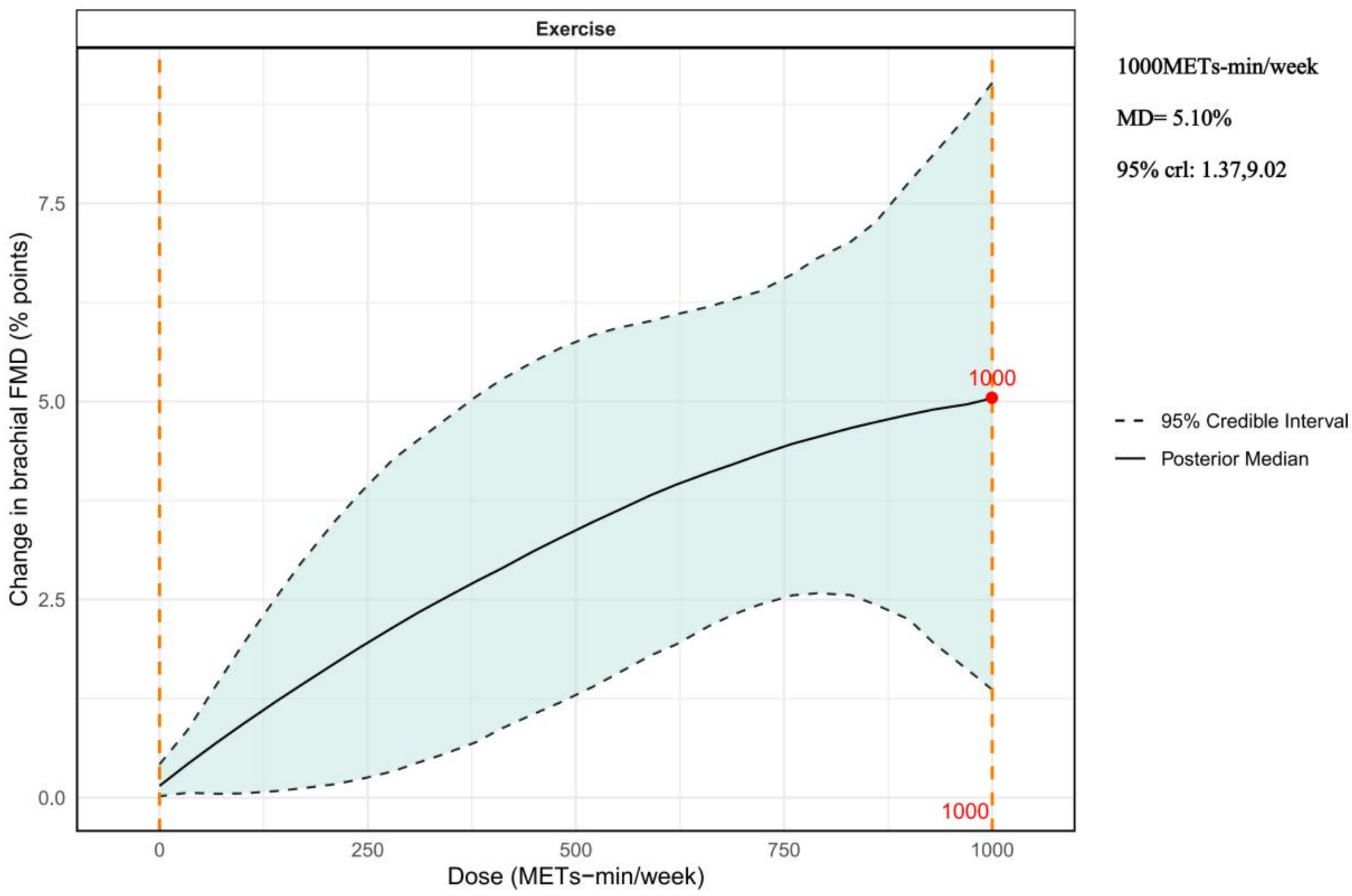

Figure S13. Dose-response relationship between exercise and brachial flow-mediated dilation in interventions lasting 4-8 weeks.

The orange dashed lines indicate the modeled dose range from～1000 METs-min/week. The solid line represents the posterior median, and the dashed curves represent the 95% credible interval. The light green shaded area shows the uncertainty band around the dose-response curve. The red dot marks the dose associated with the largest estimated improvement in brachial FMD within the modeled range, 1000 METs-min/week.

6.5.8 Intervention Duration (10-12 Weeks)

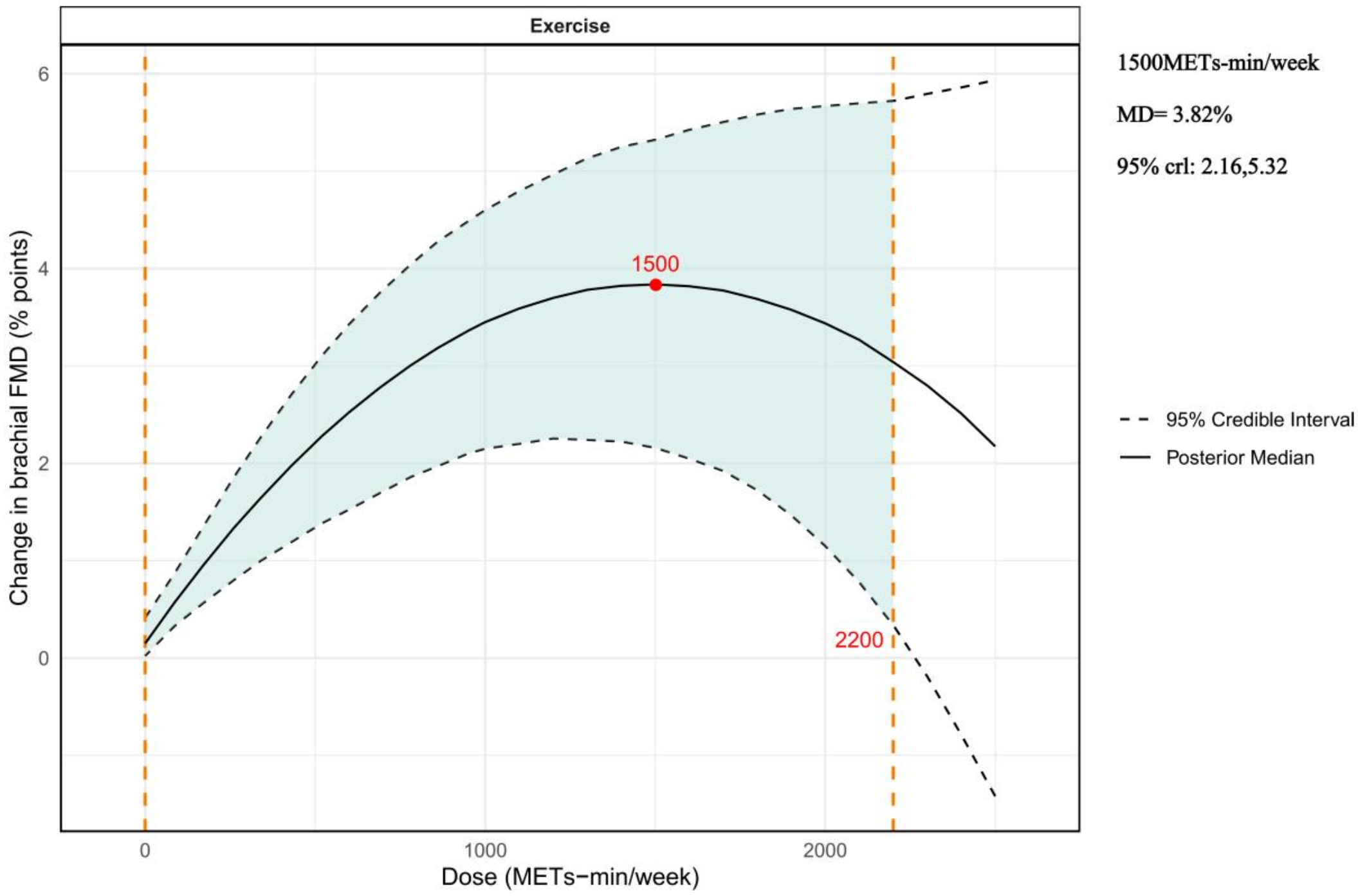

Figure S14. Dose-response relationship between exercise and brachial flow-mediated dilation in interventions lasting 10-12 weeks.

The orange dashed lines indicate the modeled dose range from～2200 METs-min/week. The solid line represents the posterior median, and the dashed curves represent the 95% credible interval. The light green shaded area shows the uncertainty band around the dose-response curve. The red dot marks the dose associated with the largest estimated improvement in brachial FMD within the modeled range, 1500 METs-min/week.

6.5.9 Intervention Duration (>12 Weeks)

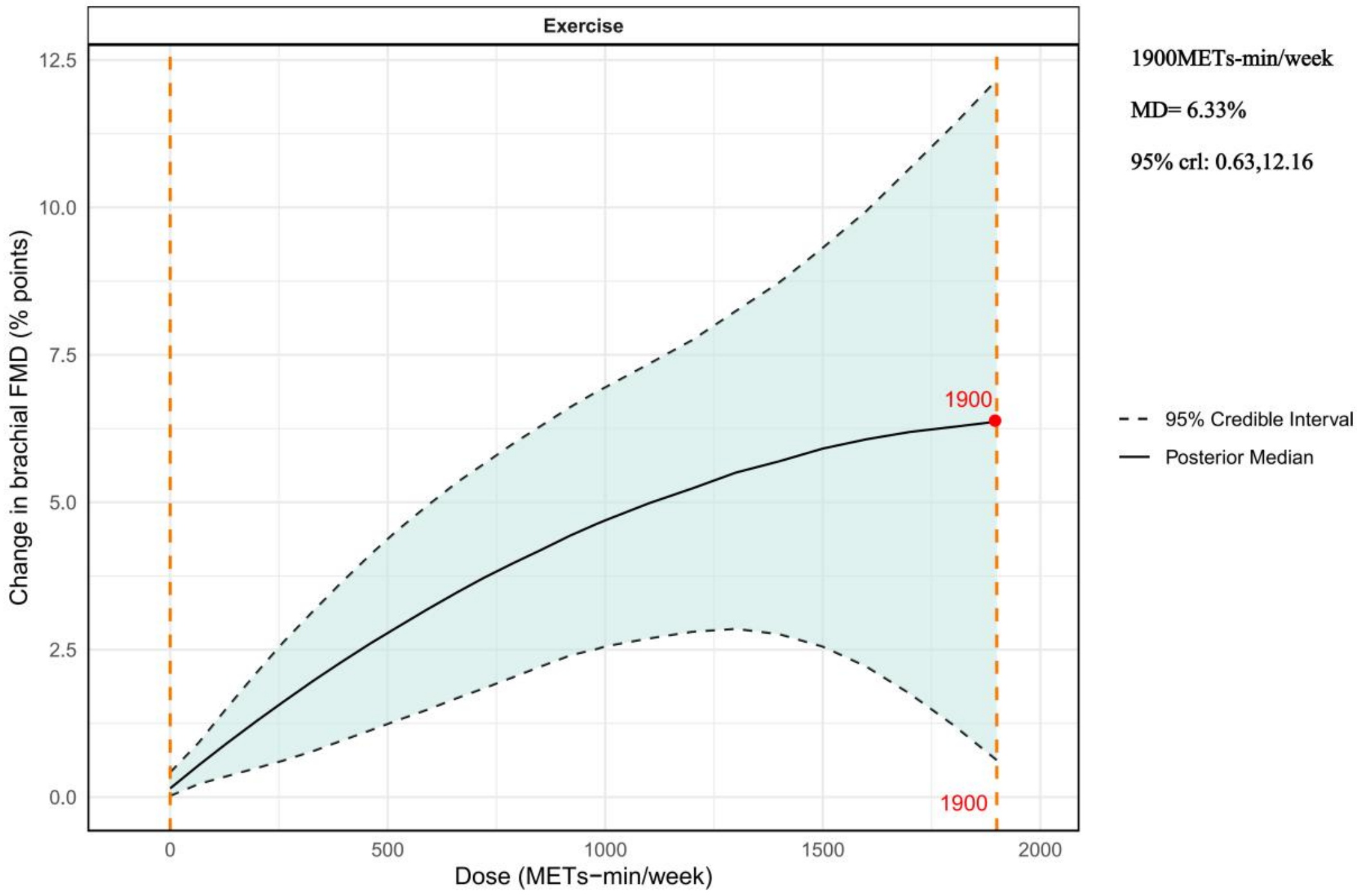

Figure S15. Dose-response relationship between exercise and brachial flow-mediated dilation in interventions lasting ＞12 weeks.

The orange dashed lines indicate the modeled dose range from～1900 METs-min/week. The solid line represents the posterior median, and the dashed curves represent the 95% credible interval. The light green shaded area shows the uncertainty band around the dose-response curve. The red dot marks the dose associated with the largest estimated improvement in brachial FMD within the modeled range, 1900 METs-min/week.

6.6 Sensitivity analysis

6.6.1 Flow-mediated dilation(excluding studies at high risk of bias)

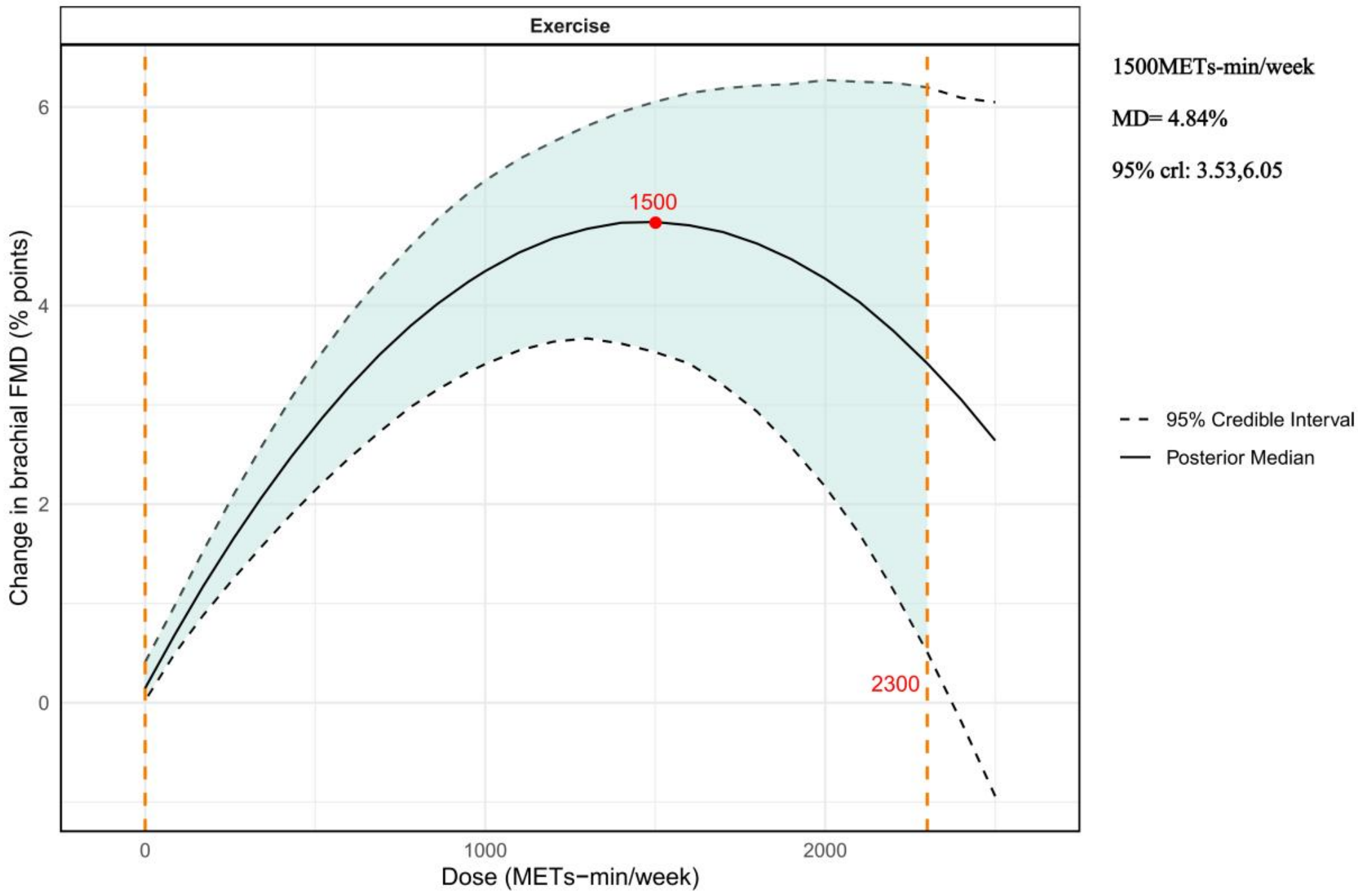

Figure S16. Dose-response relationship between exercise and brachial flow-mediated dilation after excluding studies at high risk of bias.

The orange dashed lines indicate the modeled dose range from ～2300 METs-min/week. The solid line represents the posterior median, and the dashed curves represent the 95% credible interval. The light green shaded area shows the uncertainty band around the dose-response curve. The red dot marks the dose associated with the largest estimated improvement in brachial FMD within the modeled range, 1500 METs-min/week.

6.7 Effectiveness ranking of different exercises and doses

6.7.1 Table S7. Predictive ranking of different exercise doses (from best to worst).

Rank	Treatment	Mean	Median	2.50%	97.50%
1	AE-RT_1400	6.116	4	1	22.025
2	AE-RT_1500	6.383333333	4	1	24
3	AE-RT_1300	6.987	5	1	24
4	AE-RT_1600	7.661333333	5	1	29
5	AE-RT_1200	8.831	7	1	27
6	AE-RT_1700	9.793666667	7	1	35
7	AE-RT_1100	11.26766667	9	3	31
8	AE-RT_1800	12.71	10	1	45.025
9	AE-RT_1000	14.147	12	5	37
10	AE-RT_1900	16.683	12	2	59
11	AE-RT_950	16.308	14	7	40
12	AE-RT_2000	22.06633333	15	3	74
13	AE-RT_860	19.89066667	17	8	47

14	AE-RT_2100	29.40566667	20	4	97
15	AE-RT_780	23.98766667	21	10	53
16	RT_900	30.21133333	26	1	82
17	AE_1200	30.37733333	27	3	71
18	AE-RT_690	29.96733333	27	13	62
19	AE_1100	30.55433333	28	7	66
20	AE_1300	32.15966667	28	2	79
21	RT_870	32.01333333	28	2	81
22	AE-RT_2200	38.95433333	28.5	4	122.025
23	RT_840	33.98933333	30	3	79.025
24	AE_1000	32.61	31	11	65
25	AE_1400	35.75733333	31	1	93
26	AE_950	34.69766667	33	14	66.025
27	RT_810	36.143	33	6	80
28	MBE_500	51.11	35	1	168
29	RT_780	38.48166667	35	9	82.025
30	AE_900	37.20666667	36	16	68
31	AE-RT_600	38.376	36	15	73
32	HIIT_1000	38.95366667	36	1	90
33	AE_850	40.11733333	38	19	71
34	HIIT_970	39.84133333	38	2.975	87
35	MBE_480	53.499	38	2	167
36	RT_740	41.55366667	38	13	84.025
37	HIIT_930	41.137	39	6	84
38	AE-RT_2300	51.01833333	40	6	149
39	HIIT_900	42.57766667	41	9	83
40	MBE_470	55.15333333	41	3	166
41	RT_710	44.27933333	41	16	86
42	AE_800	43.381	42	21	73.025
43	HIIT_860	44.561	44	12	82
44	RT_680	47.12666667	44	19	88
45	MBE_450	57.793	45	4	165
46	AE_750	47.03833333	46	24	77
47	HIIT_830	46.49233333	46	14	82
48	AE-RT_520	48.61033333	48	18	83
49	RT_650	50.21833333	48	22	91
50	HIIT_790	49.00933333	49	17	83
51	AE_700	51.11233333	50	27	81
52	MBE_430	60.60566667	50	5	164
53	HIIT_760	51.34833333	51	20	84
54	RT_620	53.50933333	52	25	93
55	AE_650	55.65233333	55	30	85.025
56	HIIT_720	54.323	55	22	86.025
57	MBE_410	63.617	55	6	163

58	RT_590	56.98766667	55	28	95
59	AE-RT_2400	65.25833333	56	8	167
60	HIIT_690	57.00166667	58	24	88
61	MBE_400	65.627	58	7	162
62	RT_560	60.665	59	31	99
63	AE_600	60.73333333	60	34	90.025
64	HIIT_660	59.801	61	27	90
65	AE-RT_430	63.26733333	63	30	97
66	MBE_380	68.92166667	63	8	161
67	RT_530	64.51033333	63	34	102
68	HIIT_620	63.47733333	64	30	93
69	AE_550	66.299	66	39	95.025
70	HIIT_590	66.604	67	33	95
71	RT_500	68.63833333	67	39	105.025
72	MBE_360	72.46066667	68	9	160
73	HIIT_550	70.759	71	38.975	98
74	AE_500	72.44833333	72	45	102
75	RT_470	72.89433333	72	44	109
76	MBE_340	76.239	73	10	159
77	AE-RT_2500	81.08	75	10	168
78	HIIT_520	74.32533333	75	42	101
79	MBE_330	78.63433333	76	11	158
80	RT_430	78.55266667	78	49	114
81	AE_450	79.17633333	79	51	109
82	HIIT_480	78.98766667	80	48	105
83	AE-RT_340	80.78966667	81	48	112
84	MBE_310	82.737	81	18	157
85	RT_400	83.28	83	53	117
86	HIIT_450	83.00866667	84	53	108
87	MBE_290	87.07566667	86	22	156
88	AE_400	86.51666667	87	57	115.025
89	RT_370	88.198	88	58	121
90	HIIT_410	88.36266667	89	60	113.025
91	MBE_280	89.753	89	25	155
92	RT_340	93.325	93	64	125
93	HIIT_380	92.90033333	94	66	118
94	MBE_260	94.44266667	94	29	154
95	AE_350	94.51266667	95	66	122
96	RT_310	98.701	98	71	128
97	AE-RT_260	98.67766667	99	69	127
98	MBE_240	99.38233333	99	34	153
99	MICT_790	101.3343333	99	41	166
100	HIIT_340	99.077	100	73	123
101	MICT_760	101.3193333	100	44	165

102	MICT_820	101.609	100	38	167
103	MICT_850	102.1593333	100	34.975	168
104	MICT_730	101.621	101	46.975	163
105	MICT_700	102.191	102	51	162
106	AE_300	103.332	103	76	129
107	MICT_670	103.0526667	103	54	160
108	RT_280	104.4273333	104	76.975	132
109	HIIT_310	104.3156667	105	78	127
110	MBE_220	104.5413333	105	41.975	152
111	MICT_640	104.1733333	105	58	158
112	MICT_620	105.2606667	107	61	156
113	MBE_210	107.5976667	108	47	151
114	MICT_590	106.8326667	108	65	154
115	RT_250	110.411	109	82	136
116	HIIT_280	109.7933333	110	84	132
117	MICT_560	108.6426667	110	69.975	151
118	AE_250	112.868	113	84	136
119	MICT_530	110.662	113	74	147.025
120	MBE_190	112.974	114	57	150
121	MICT_500	112.8723333	115	78	145
122	RT_220	116.7096667	117	87	140
123	HIIT_240	117.215	118	89.975	137
124	MICT_470	115.2563333	118	82.975	142
125	MBE_170	118.5086667	120	67	152
126	MICT_440	117.7576667	121	86.975	141
127	MICT_410	120.451	123	91	141
128	AE-RT_170	121.4446667	124	90	142
129	HIIT_210	123.4363333	125	95	141
130	MBE_160	121.77	125	72.975	154
131	AE_200	123.224	126	92	143
132	MICT_380	123.2836667	126	95.975	141.025
133	RT_190	123.3766667	126	92	144
134	MICT_350	126.218	129	100	143
135	MBE_140	127.5173333	131	84	155
136	MICT_320	129.268	132	104	145
137	RT_160	130.352	134	97	148
138	HIIT_170	131.6266667	135	104.975	146.025
139	MICT_290	132.4293333	135	108	147
140	MBE_120	133.3053333	138	96	157.025
141	AE_150	134.126	139	106	150
142	MICT_260	135.6756667	139	111	149
143	HIIT_140	138.0943333	142	114	151
144	MICT_230	139.0106667	142	115	152
145	MBE_100	139.238	143	106	159

146	MICT_210	141.5963333	144	118	153
147	RT_120	139.467	144	113.975	154
148	MBE_86	143.6806667	146	113	161
149	MICT_180	145.0946667	148	121	156
150	AE-RT_86	144.763	149	120	156
151	AE_100	145.6363333	150	121.975	157
152	RT_93	146.301	150	121.975	158
153	HIIT_100	146.97	151	123	157
154	MBE_69	148.839	151	121	163
155	MICT_150	148.712	151	124	158
156	MICT_120	152.459	155	128	161
157	MBE_52	153.9483333	156	127	165
158	HIIT_69	153.6106667	157	130	161
159	RT_62	153.6353333	157	129	162
160	MICT_88	156.4553333	159	131	163
161	AE_50	156.9946667	160	133	163
162	MBE_34	159.099	161	135.975	166
163	MICT_59	160.146	162	135	166
164	HIIT_34	161.07	164	136	166
165	RT_31	160.949	164	135	166
166	MBE_17	163.6123333	166	140	167
167	MICT_29	164.0606667	166	138	167
168	Placebo_0	166.1476667	168	139	168

Note: The number that follows the exercise intervention indicates the exercise dose (MET-min/week).AE: Aerobic Exercise; AE-RT: Combined Aerobic and Resistance Training; RT: Resistance Training;MBE:Mind-body exercise;HIIT: High-Intensity Interval Training;MICT:Moderate-Intensity Continuous Training;CG: Control group

6.8 Meta-regression

Table S11. Dose-response network meta-regression for brachial FMD.

Covariate	DIC	Residual deviance	Brachial FMD<br>Shared beta median (95% CrI)	SD
Age (years)	360.1	145.1	-0.07 (-0.18, 0.05)	0.26 (0.20, 0.37)
Baseline brachial FMD (%)	369.3	152.4	0.23 (-0.17, 0.61)	0.98 (0.72, 1.35)
Intervention length (weeks)	350.1	137.2	-0.13 (-0.29, 0.02)	0.31 (0.23, 0.44)
Baseline BMI (kg/m^2)	332.4	124.1	-0.15 (-0.47, 0.15)	0.73 (0.54, 1.00)

8. Publication bias

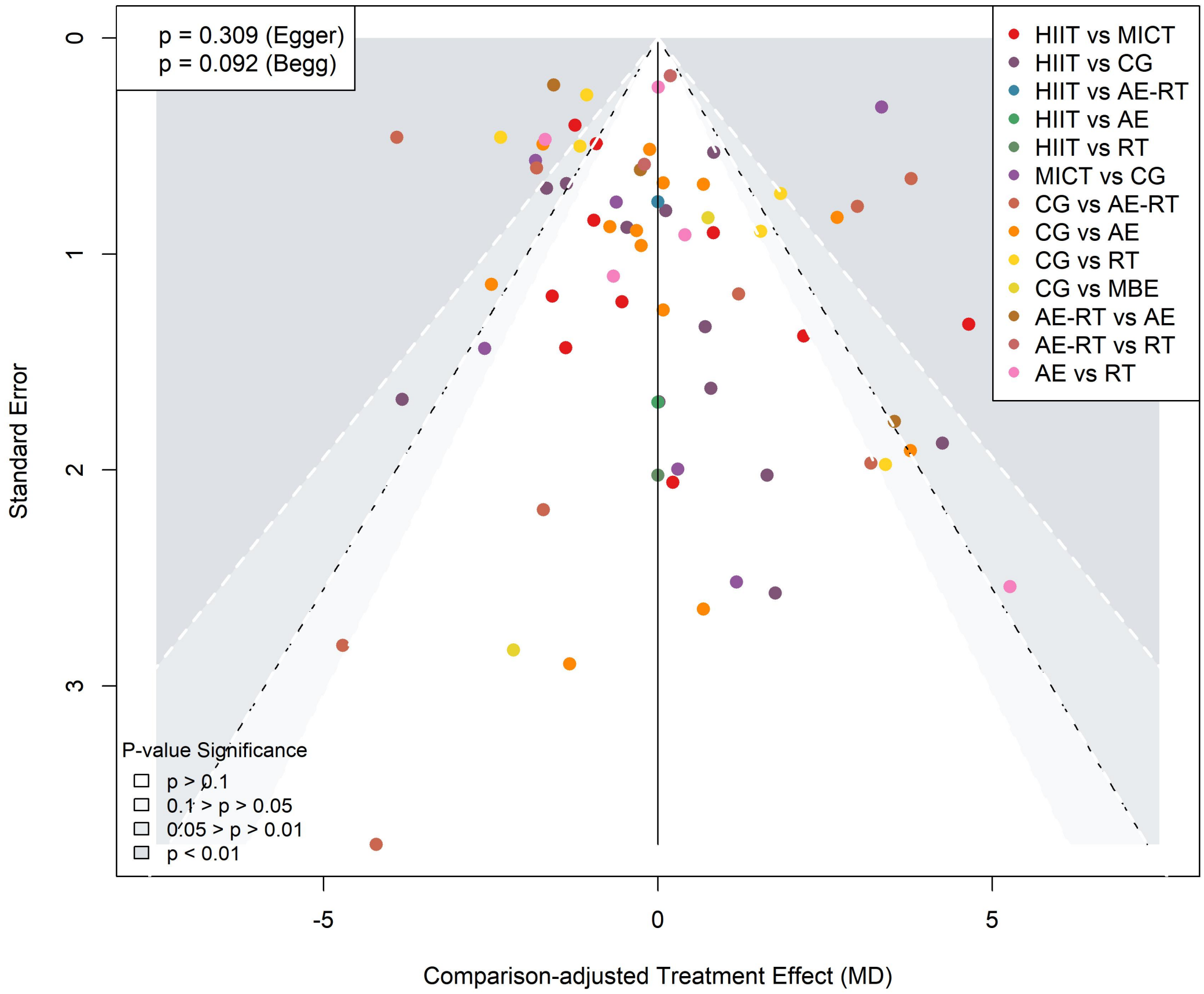

Figure S17. Funnel plot

AE: Aerobic Exercise; AE-RT: Combined Aerobic and Resistance Training; RT: Resistance Training;MBE:Mind-body exercise;HIIT: High-Intensity Interval Training;MICT:Moderate-Intensity Continuous Training;CG: Control group

9. GRADE Summary of All Studies

Based on the CINeMA online guidelines (https://cinema.ispm.unibe.ch/), we evaluated the certainty of evidence for the primary outcome, brachial artery flow-mediated dilation (FMD). Downgrading decisions were made according to the following domains: within-study bias, reporting bias, indirectness, imprecision, heterogeneity, and incoherence.

9.1 Within-study Bias

Each included study was assessed using the Cochrane RoB 2 tool. Studies were classified as low risk of bias, some concerns, or high risk of bias. For each network comparison, the overall within-study bias judgment was informed by the contribution of studies with different risk-of-bias levels. No downgrade was applied when there were no important concerns; one level was downgraded when some concerns were present; and two levels were downgraded when major concerns or a substantial contribution from high-risk studies was identified.

9.2 Reporting Bias

Reporting bias was assessed by considering publication bias and selective outcome reporting.

We searched multiple bibliographic databases and one trial registry, and additionally checked relevant systematic reviews and reference lists to minimize the risk of missing eligible trials. Publication bias was evaluated using comparison-adjusted funnel plots and, when appropriate, Egger's or Begg's tests. Selective reporting was judged by comparing the outcomes described in the methods, trial registration records, or protocols with the reported FMD results. If there was no evidence of small-study effects or selective reporting, this domain was not downgraded.

9.3 Indirectness

Indirectness was assessed according to the clinical and methodological relevance of the included studies to the research question. Eligible studies included adults with overweight or obesity, evaluated structured exercise interventions lasting at least 4 weeks, used appropriate control or comparator groups, and reported brachial artery FMD as the outcome. Potential sources of indirectness included variation in cardiometabolic disease status, baseline BMI, baseline endothelial function, intervention duration, and FMD measurement protocols. These factors were further examined in subgroup and sensitivity analyses. Downgrading was applied when the evidence for a comparison was mainly derived from populations, interventions, or measurement conditions that were not closely aligned with the target question.

9.4 Imprecision

Brachial FMD was analyzed as a continuous outcome and expressed as change in FMD percentage points. Mean difference was used as the effect measure. Because there is no universally accepted minimum clinically important difference for changes in brachial FMD in adults with overweight or obesity, the null value of 0 was used as the primary reference threshold. Evidence was downgraded for imprecision when credible intervals were wide, crossed the null value, or were compatible with both clinically meaningful benefit and little or no effect.

9.5 Heterogeneity

Heterogeneity was assessed by considering both statistical and clinical heterogeneity. Clinical heterogeneity may arise from differences in baseline FMD, BMI category, cardiometabolic disease status, age, sex distribution, intervention duration, exercise supervision, and exercise dose. Statistical heterogeneity was assessed using model-based heterogeneity estimates and prediction intervals where available. Evidence was downgraded when heterogeneity was substantial and materially affected the interpretation of the estimated exercise effect or dose-response relationship.

9.6 Incoherence

Incoherence was assessed by examining disagreement between direct and indirect evidence in the network. Local and global inconsistency checks were used when feasible. No downgrade was applied when there was no evidence of important incoherence; one level was downgraded when possible incoherence was detected; and two levels were downgraded when clear incoherence was present and could not be explained by clinical or methodological differences across studies.

9.7 Summarizing Judgments Across CINeMA Domains

The CINeMA framework assesses confidence in evidence across six domains: within-study bias, reporting bias, indirectness, imprecision, heterogeneity, and incoherence. These domain-level judgments were summarized into an overall certainty rating using the GRADE

approach: high, moderate, low, or very low. When the same issue affected multiple domains, such as clinical heterogeneity contributing to both indirectness and imprecision, judgments were considered together to avoid redundant downgrading. The final certainty rating reflected the overall confidence that the estimated effects of exercise modalities and doses on brachial FMD were close to the true effects.

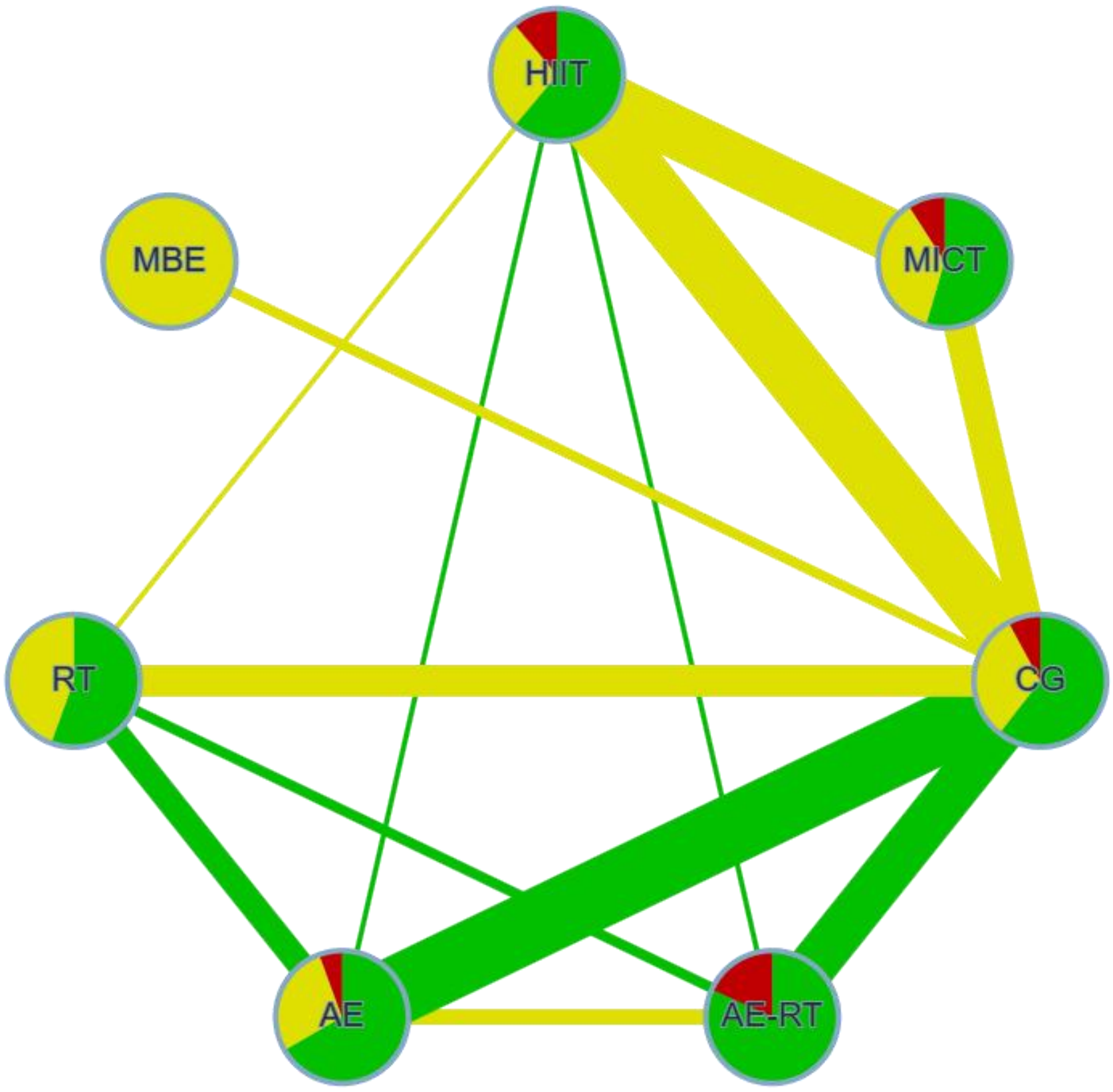

Figure S18. Network plot of study limitations of the included studies

The colors in the circles indicate the percentage of low RoB studies (green), moderate RoB studies (yellow), high RoB studies (red) about each physical activity type. Edge width by the number of studies. Edge color by average RoB. The colors of the lines indicate the summative RoB assessment of each comparison. Low RoB is green, moderate RoB is yellow, high RoB is red.AE: Aerobic Exercise; AE-RT: Combined Aerobic and Resistance Training; RT: Resistance Training;MBE:Mind-body exercise;HIIT: High-Intensity Interval Training;MICT:Moderate-Intensity Continuous Training;CG: Control group

Table S9 The confidence of evidence using the CINeMA

Comparison	Within-study bias	Reporting bias	Indirectness	Imprecision	Heterogeneity	Incoherence	Confidence rating	Reason(s) for downgrading
AE:AE-RT	No concerns	Low risk	No concerns	Major concerns	No concerns	No concerns	**Low**	["Imprecision"]
**AE:CG**	**No concerns**	**Low risk**	**No concerns**	**No concerns**	**Major concerns**	**No concerns**	**Low**	**["Heterogeneity"]**
AE:HIIT	No concerns	Low risk	No concerns	Major concerns	No concerns	No concerns	**Low**	["Imprecision"]
AE:RT	No concerns	Low risk	No concerns	Major concerns	No concerns	No concerns	Low	["Imprecision"]
**AE-RT:CG**	**No concerns**	**Low risk**	**No concerns**	**No concerns**	**Major concerns**	**No concerns**	**Low**	**["Heterogeneity"]**
AE-RT:HIIT	No concerns	Low risk	No concerns	Major concerns	No concerns	No concerns	**Low**	["Imprecision"]
AE-RT:RT	No concerns	Low risk	No concerns	Major concerns	No concerns	No concerns	**Low**	["Imprecision"]
**HIIT:CG**	**No concerns**	**Low risk**	**No concerns**	**No concerns**	**Major concerns**	**No concerns**	**Low**	**["Heterogeneity"]**
**MBE:CG**	**Some concerns**	**Low risk**	**No concerns**	**Major concerns**	**No concerns**	**Major concerns**	**Very low**	**["Within-study bias","Imprecision","Incoherence"]**
**MICT:CG**	**Some concerns**	**Low risk**	**No concerns**	**No concerns**	**Major concerns**	**No concerns**	**Very low**	**["Within-study bias","Heterogeneity"]**
**RT:CG**	**No concerns**	**Low risk**	**No concerns**	**No concerns**	**Major concerns**	**No concerns**	**Low**	**["Heterogeneity"]**
HIIT:MICT	Some concerns	Low risk	No concerns	Major concerns	No concerns	Major concerns	**Very low**	["Within-study bias","Imprecision","Incoherence"]
HIIT:RT	No concerns	Low risk	No concerns	Major concerns	No concerns	No concerns	**Low**	["Imprecision"]

AE: Aerobic Exercise; AE-RT: Combined Aerobic and Resistance Training; RT: Resistance Training;MBE:Mind-body exercise;HIIT: High-Intensity Interval Training; MICT:Moderate-Intensity Continuous Training;CG: Control group

Table S13 Summary of dose-response network meta-analysis result.

Category	Data points	Outcome	Modeled dose range (MET-min/week)	Optimal dose (MET-min/week)	Observed dose range (MET-min/week)	Mean difference and 95% CrI (optimal dose)
Overall exercise	107	FMD	~2300	1500	150-2500	4.72 %-points (3.43, 5.91)
Baseline FMD ≤6.5%	60	FMD	~1400	1400	200-1450	4.30 %-points (1.87, 6.85)
Baseline FMD >6.5%	40	FMD	~2200	1400	150-2500	5.94 %-points (3.86, 8.14)
BMI 25.0-29.9 kg/m²	65	FMD	~2300	1400	150-2500	4.99 %-points (3.71, 6.27)
BMI ≥30 kg/m²	30	FMD	~1000	1000	250-1200	3.51 %-points (0.53, 6.53)
Apparently healthy	32	FMD	~2000	1400	150-2500	5.49 %-points (2.22, 8.84)
Cardiometabolic disease	75	FMD	~1900	1800	200-1900	4.87 %-points (1.90, 7.88)
Length (4-8 weeks)	30	FMD	~1000	1000	150-1000	5.10 %-points (1.37, 9.02)
Length (10-12 weeks)	54	FMD	~2200	1500	150-2500	3.82 %-points (2.16, 5.32)
Length (>12 weeks)	23	FMD	~1900	1900	300-1900	6.33 %-points (0.63, 12.16)
Sensitivity analysis	98	FMD	~2300	1500	150-2500	4.84 %-points (3.53, 6.05)
AE	18	FMD	~1400	1200	400-1450	4.15 %-points (2.53, 5.68)
RT	9	FMD	~900	900	450-900	4.16 %-points (2.25, 6.05)
HIIT	18	FMD	~1000	1000	150-1000	3.75 %-points (1.93, 5.65)
MICT	11	FMD	~760	760	300-850	1.86 %-points (0.04, 3.62)
AE-RT	11	FMD	~2400	1500	250-2500	5.77 %-points (4.04, 7.52)
MBE	2	FMD	~140	140	300-500	1.31%-points (0.002, 2.65)

Note: Data points in the modality-specific rows denote the arm-level data points of each modality; the overall and subgroup rows additionally include the shared control arms;Optimal doses falling outside the observed dose range should be regarded as extrapolations of limited reliability; this applies most notably to mind-body exercise, whose estimated optimal dose (140

MET-min/week) lies below its observed dose range (300 – 500 MET-min/week).AE: Aerobic Exercise; AE-RT: Combined Aerobic and Resistance Training; RT: Resistance Training;MBE:Mind-body exercise;HIIT: High-Intensity Interval Training; MICT:Moderate-Intensity Continuous Training

Table S14. Model-based exercise-dose translations for improving brachial FMD.

Outcome	Exercise modality	Energy expenditure (METs)[a]	Recommended accumulation (min/week)	Optimal recommended Accumulation (min/week)	Certainty of evidence (grade)
Brachial FMD	Cycling	7 (code: 01016)[b]	~170	200	Low
	Jogging	4.8(code:12025)[c]	~290	250	Low
	Walking	3.5(code:17160)[d]	~400	345	Low
	Resistance training	3.5(code:02054)[e]	~255	255	Low
	Cycling combined with resistance training	7(code: 01016)<br>3.5(code:02054)	Cycling: ~170；<br>Resistance training: ~345	Cycling: 105；<br>Resistance training: 215	Low
	Jogging combined with resistance training	4.8(code:12025)<br>3.5(code:02054)	Jogging: ~250；<br>Resistance training: ~345	Jogging: 155；<br>Resistance training: 215	Low
	Yoga	2.3(code:02175)[f]	~60	60	Very low
	HIIT	11.0(code:02214)[g]	~90	90	Low

Note: *Exercise intensity was coded based on the 2024 standardized physical activity intensity tables.[a]METs-min: Metabolic Equivalents of Task minutes. [b]Cycling: self-selected moderate pace. [c]Jogging, in place. [d]Walking for pleasure. [e]Resistance (weight) training, multiple exercises, 8-15 reps at varied resistance.[f]Yoga:general.[g]HIIT:High intensity interval exercise, burpees, mountain climbers, squat jumps, Tabata, vigorous effort. 3-6 METs represents moderate-intensity activity, and >6 METs represents high-intensity activity, serving as a reference for absolute exercise intensity.

10. Network transitivity assessment

To visually assess the plausibility of the transitivity assumption, boxplots were used to examine the distribution of key effect modifiers across treatment groups. Between-group differences were explored using statistical tests to identify potential imbalance. Data normality was assessed using the Shapiro-Wilk test; one-way ANOVA was applied for normally distributed variables, and the Kruskal-Wallis test was applied for non-normally distributed variables. A P-value <0.05 was considered indicative of statistically significant imbalance that could challenge the plausibility of transitivity.

No statistically significant imbalance was observed for age (P=0.88), BMI (P=0.94), baseline brachial FMD (P=0.66), or female percentage (P=0.57) across treatment groups (Figures S19-S22). These findings supported the plausibility of transitivity with respect to these measured study-level characteristics. However, health status, medication use, disease severity, exercise supervision, and exercise tolerance were not uniformly reported or fully balanced across trials. Therefore, the transitivity assumption was considered plausible but not definitively established.

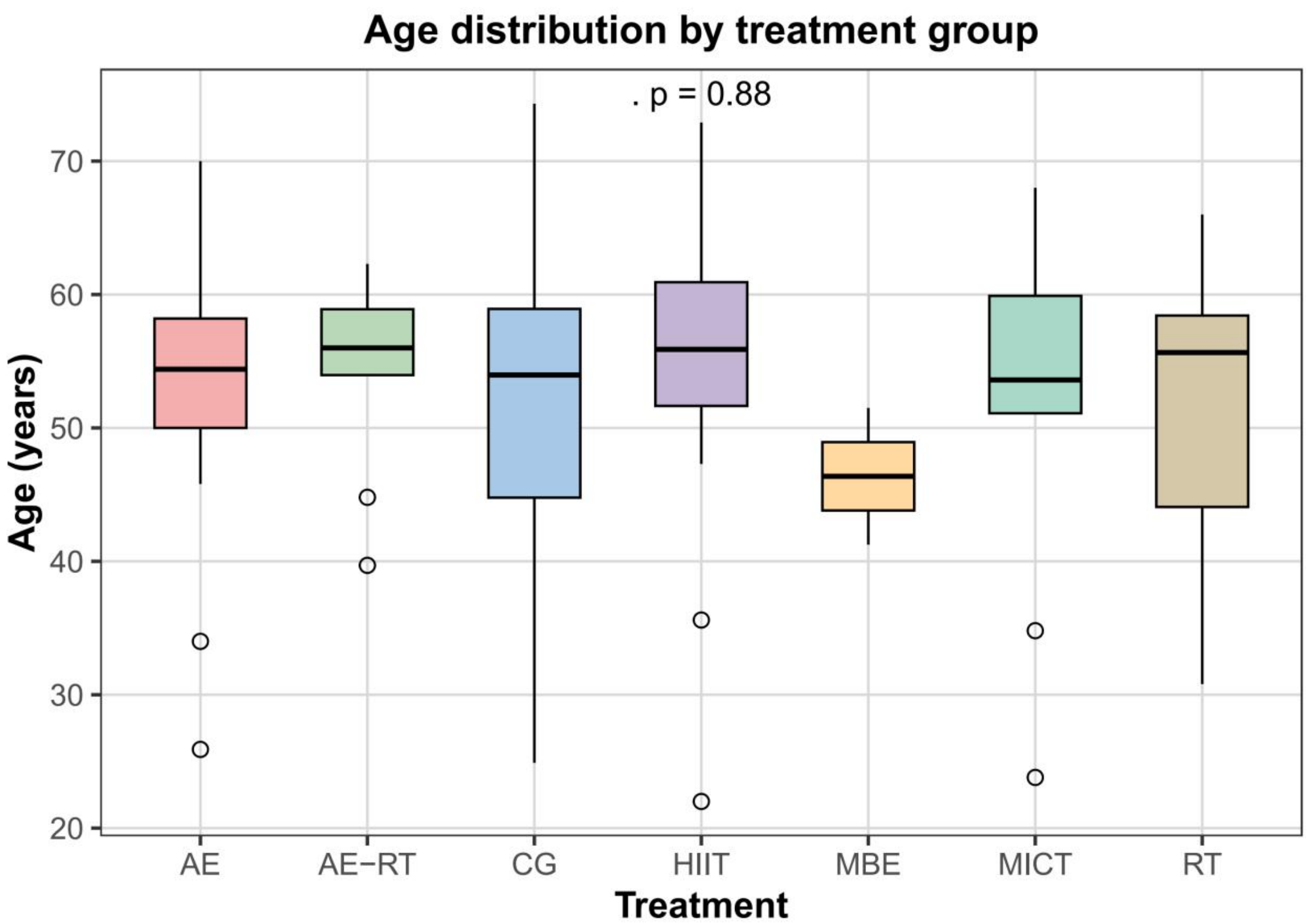

Figure S19. Comparison of age distributions across treatment groups for evaluating transitivity.

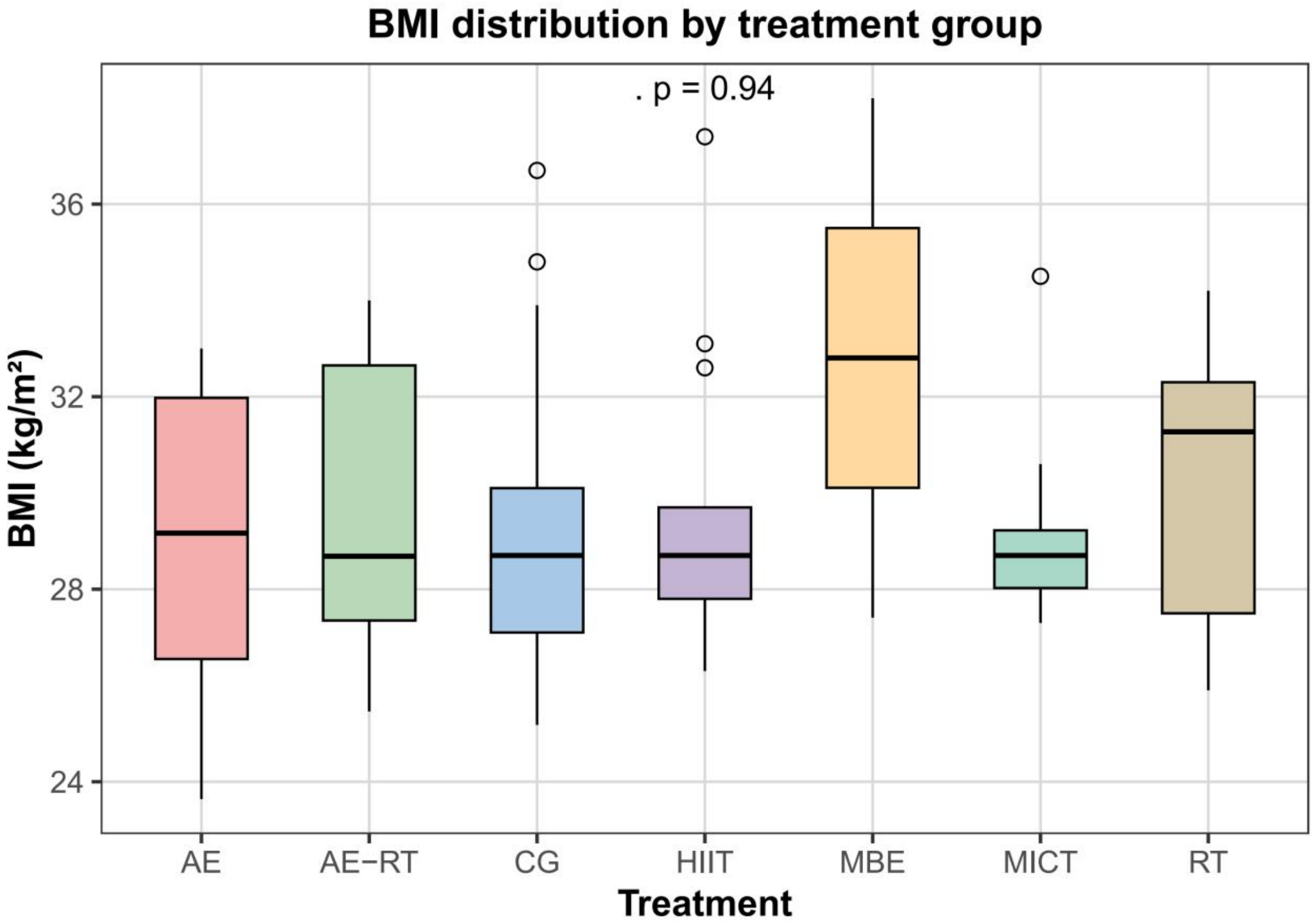

Figure S20. Comparison of BMI distributions across treatment groups for evaluating transitivity.

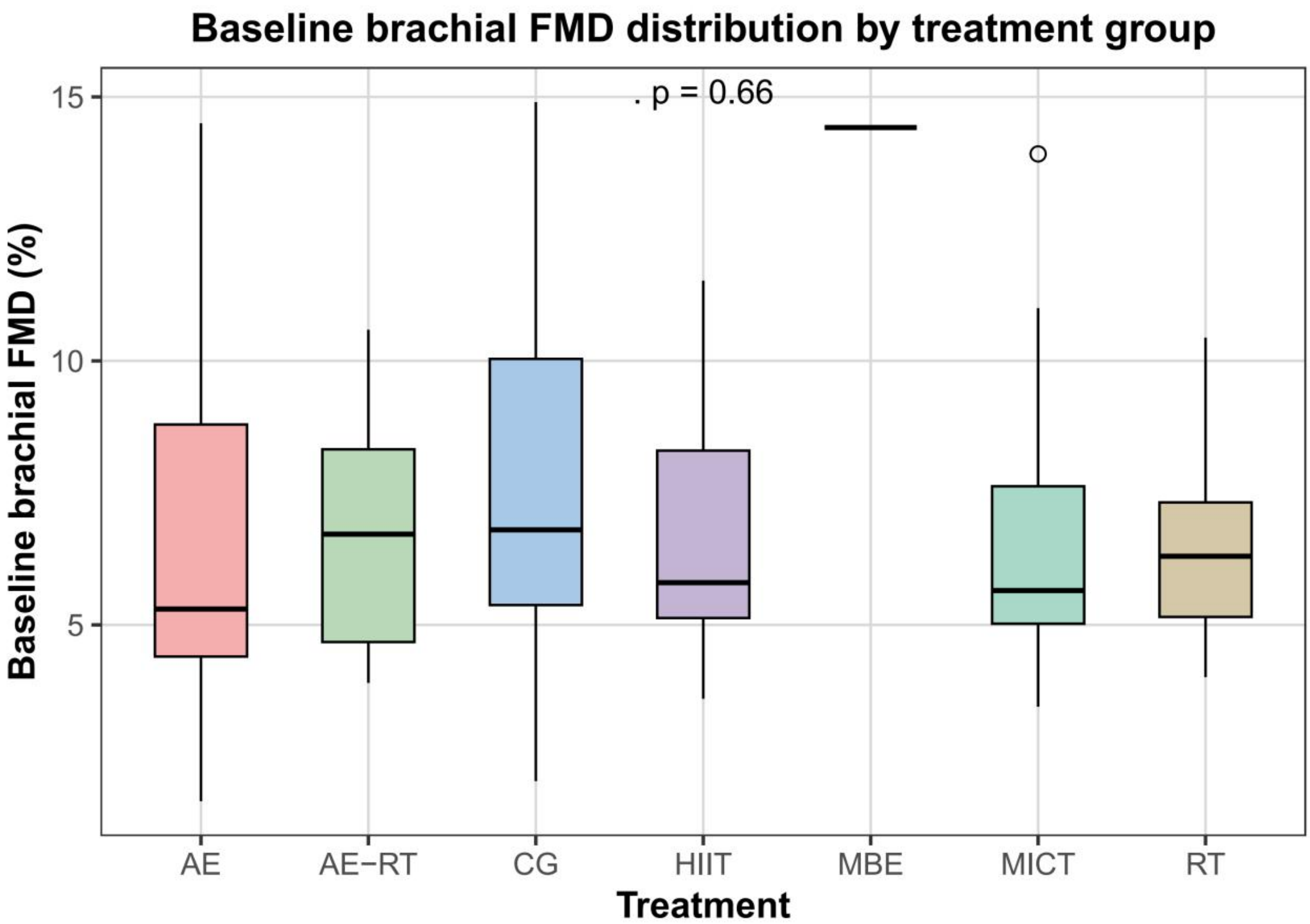

Figure S21. Comparison of baseline brachial FMD distributions across treatment groups for evaluating transitivity.

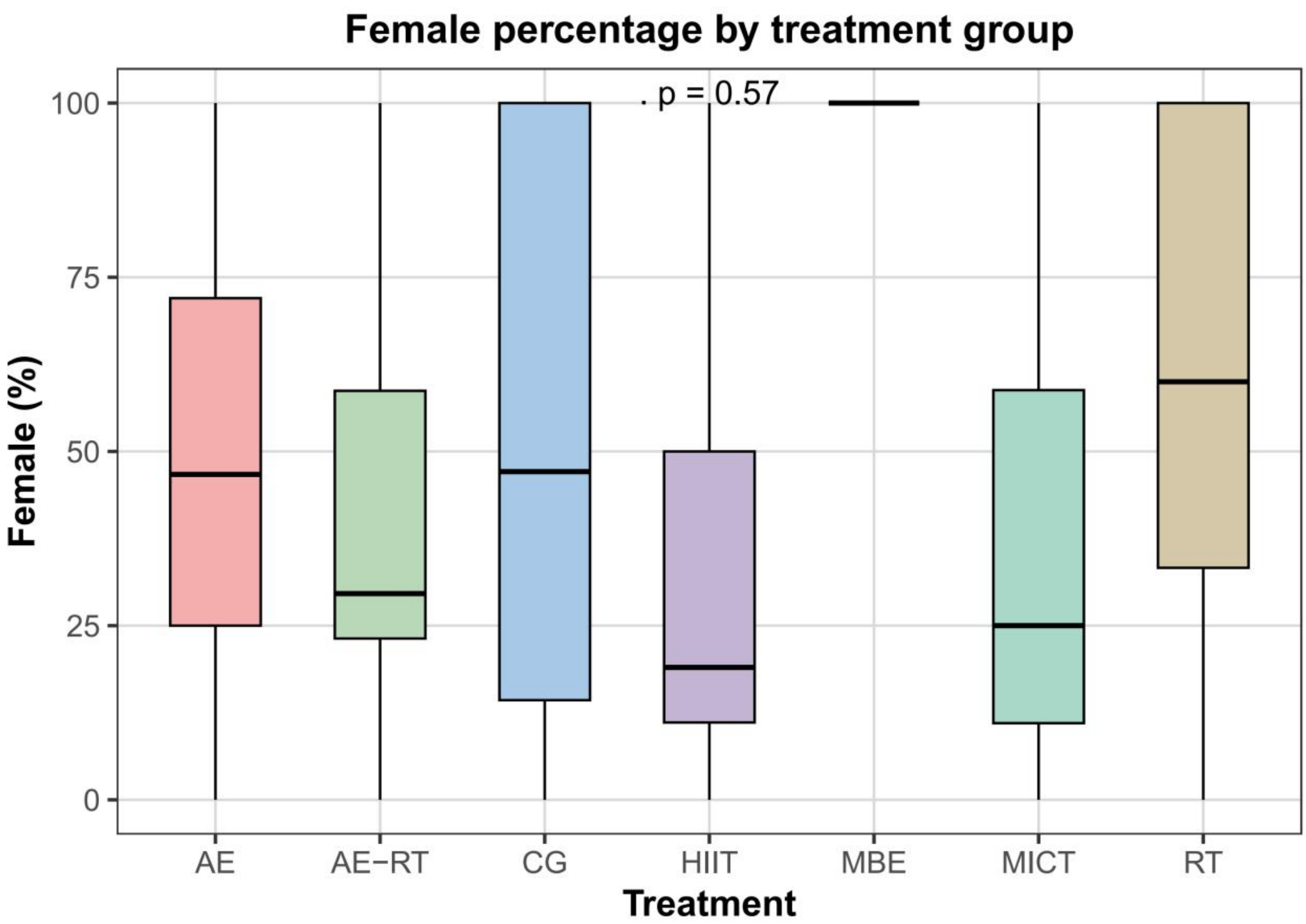

Figure S22. Comparison of female percentage distributions across treatment groups for evaluating transitivity.

Table S15. Distribution of potential effect modifiers across treatment nodes

Treatment node	Arms (K)[a]	Age, y, mean (range)	BMI, kg/m², mean (range)	Female, %, mean (range)	Baseline FMD, %, mean (range)
AE	18	51.5 (25.9-70.0)	29.2 (25.5-33.0)	51.5 (0-100)	6.4 (1.7-14.5)
RT	9	49.1 (30.0-66.0)	30.2 (25.9-34.2)	64.5 (0-100)	6.1 (4.0-10.4)
AE-RT	11	52.9 (37.5-62.3)	29.2 (25.5-34.0)	35.7 (0-100)	7.1 (3.9-10.6)
HIIT	18	52.6 (20.2-72.9)	29.3 (26.3-37.4)	39.9 (6.7-100)	6.7 (3.6-11.5)
MICT	11	52.8 (23.8-68.0)	29.4 (27.3-34.5)	39.8 (0-100)	6.6 (3.5-13.9)
MBE	2	46.4 (41.2-51.5)	32.8 (27.4-38.2)	89.5 (78.9-100)	9.2 (3.9-14.4)
Control	38	50.9 (20.7-74.3)	29.4 (25.2-36.7)	52.0 (0-100)[b]	7.2 (2.0-14.9)

Note: Note: Values are presented as mean (range). [a] K denotes the number of arms contributing data. [b] Female percentage was recorded at the study level (proportion of women among all randomized participants, including the control arm) and is therefore shown per node using the same study-level value. AE, aerobic exercise; AE-RT, combined aerobic and resistance training; BMI, body mass index; FMD, flow-mediated dilation; HIIT, high-intensity interval training; MBE, mind-body exercise; MICT, moderate-intensity continuous training; RT, resistance training.

Reference

1. Ghardashi Afousi A, Izadi MR, Rakhshan K, Mafi F, Biglari S, Gandomkar Bagheri H. Improved brachial artery shear patterns and increased flow-mediated dilatation after low-volume high-intensity interval training in type 2 diabetes. *Experimental Physiology*. 2018;103(9):1264-1276. doi:10.1113/EP087005

2. Alvarez C, Peñailillo L, Ibacache-Saavedra P, et al. Six weeks of a concurrent training therapy improves endothelial function and arterial stiffness in hypertensive adults with minimum non-responders. *Hipertensión y Riesgo Vascular*. 2024;41(4):240-250. doi:10.1016/j.hipert.2024.07.001

3. Anagnostakou V, Chatzimichail K, Dimopoulos S, et al. Effects of Interval Cycle Training With or Without Strength Training on Vascular Reactivity in Heart Failure Patients. *Journal of Cardiac Failure*. 2011;17(7):585-591. doi:10.1016/j.cardfail.2011.02.009

4. Azadpour N, Tartibian B, Koşar ŞN. Effects of aerobic exercise training on ACE and ADRB2 gene expression, plasma angiotensin II level, and flow-mediated dilation: a study on obese postmenopausal women with prehypertension. *Menopause*. 2017;24(3):269-277. doi:10.1097/GME.0000000000000762

5. Barone Gibbs B, Dobrosielski DA, Bonekamp S, Stewart KJ, Clark JM. A randomized trial of exercise for blood pressure reduction in type 2 diabetes: Effect on flow-mediated dilation and circulating biomarkers of endothelial function. *Atherosclerosis*. 2012;224(2):446-453. doi:10.1016/j.atherosclerosis.2012.07.035

6. Benda NMM, Seeger JPH, Stevens GGCF, et al. Effects of High-Intensity Interval Training versus Continuous Training on Physical Fitness, Cardiovascular Function and Quality of Life in Heart Failure Patients. Hosoda T, ed. *PLoS ONE*. 2015;10(10):e0141256. doi:10.1371/journal.pone.0141256

7. Bircher S, Mucha C. Einfluss körperlicher Aktivität auf die Fettoxidation und Endothelfunktion bei Adipösen - eine randomisiert kontrollierte Studie. *Phys Rehab Kur Med*. 2007;17(6):313-319. doi:10.1055/s-2007-973832

8. Boeno FP, Ramis TR, Munhoz SV, et al. Effect of aerobic and resistance exercise training on inflammation, endothelial function and ambulatory blood pressure in middle-aged hypertensive patients. *Journal of Hypertension*. 2020;38(12):2501-2509. doi:10.1097/HJH.0000000000002581

9. Bouaziz W, Lang PO, Schmitt E, et al. Effects of a short-term interval aerobic training program with recovery bouts on vascular function in sedentary aged 70 or over: A randomized controlled trial. *Archives of Gerontology and Geriatrics*. 2019;82:217-225. doi:10.1016/j.archger.2019.02.017

10. Braith RW, Schofield RS, Hill JA, Casey DP, Pierce GL. Exercise Training Attenuates Progressive Decline in Brachial Artery Reactivity in Heart Transplant Recipients. *The Journal*

of Heart and Lung Transplantation. 2008;27(1):52-59. doi:10.1016/j.healun.2007.09.032

11. Choi JH, Kim SW, Seo J, et al. Effects of a Mobile-Health Exercise Intervention on Body Composition, Vascular Function, and Autonomic Nervous System Function in Obese Women: A Randomized Controlled Trial. *JMDH*. 2023;Volume 16:1601-1615. doi:10.2147/JMDH.S406905

12. Collins BEG, Donges C, Robergs R, Cooper J, Sweeney K, Kingsley M. Moderate continuous- and high-intensity interval training elicit comparable cardiovascular effect among middle-aged men regardless of recovery mode. *European Journal of Sport Science*. 2023;23(8):1612-1621. doi:10.1080/17461391.2023.2171908

13. Cox ER, Gajanand T, Keating SE, et al. Effect of low-volume combined aerobic and resistance high-intensity interval training on vascular health in people with type 2 diabetes: a randomised controlled trial. *Eur J Appl Physiol*. 2024;124(9):2819-2833. doi:10.1007/s00421-024-05473-8

14. Van Craenenbroeck EM, Frederix G, Pattyn N, et al. Effects of aerobic interval training and continuous training on cellular markers of endothelial integrity in coronary artery disease: a SAINTEX-CAD substudy. *American Journal of Physiology-Heart and Circulatory Physiology*. 2015;309(11):H1876-H1882. doi:10.1152/ajpheart.00341.2015

15. Currie KD, Dubberley JB, McKELVIE RS, MacDONALD MJ. Low-Volume, High-Intensity Interval Training in Patients with CAD. *Medicine & Science in Sports & Exercise*. 2013;45(8):1436-1442. doi:10.1249/MSS.0b013e31828bbbd4

16. Davoodi M, Hesamabadi BK, Ariabood E, et al. Improved blood pressure and flow-mediated dilatation via increased plasma adropin and nitrate/nitrite induced by high-intensity interval training in patients with type 2 diabetes. *Experimental Physiology*. 2022;107(8):813-824. doi:10.1113/EP089371

17. Franklin NC, Robinson AT, Bian JT, et al. Circuit Resistance Training Attenuates Acute Exertion-Induced Reductions in Arterial Function but Not Inflammation in Obese Women. *Metabolic Syndrome and Related Disorders*. 2015;13(5):227-234. doi:10.1089/met.2014.0135

18. Haykowsky M, Taylor D, Kim D, Tymchak W. Exercise Training Improves Aerobic Capacity and Skeletal Muscle Function in Heart Transplant Recipients. *American Journal of Transplantation*. 2009;9(4):734-739. doi:10.1111/j.1600-6143.2008.02531.x

19. Hermann TS, Dall CH, Christensen SB, Goetze JP, Prescott E, Gustafsson F. Effect of High Intensity Exercise on Peak Oxygen Uptake and Endothelial Function in Long-Term Heart Transplant Recipients. *American Journal of Transplantation*. 2011;11(3):536-541. doi:10.1111/j.1600-6143.2010.03403.x

20. Hildreth KL, Schwartz RS, Vande Griend J, Kohrt WM, Blatchford PJ, Moreau KL. Effects of testosterone and progressive resistance exercise on vascular function in older men. *Journal of Applied Physiology*. 2018;125(6):1693-1701. doi:10.1152/japplphysiol.00165.2018

21. Hovsepian V, Marandi SM, Esfarjani F, Zavar R, Sadeghi M. The Effect of All Extremity High Intensity Interval Training on Athero-Protective Factors and Endothelial Function in Overweight and Obese Women. *International Journal of Preventive Medicine*. 2021;12(1). doi:10.4103/ijpvm.IJPVM_248_19

22. Isaksen K, Halvorsen B, Munk PS, Aukrust P, Larsen AI. Effects of interval training on inflammatory biomarkers in patients with ischemic heart failure. *Scandinavian Cardiovascular Journal*. 2019;53(4):213-219. doi:10.1080/14017431.2019.1629004

23. Jang YJ, Park WB, Lee MG. Effects of a 12-week Equipment-based Pilates Training on Physical Fitness, Cardiovascular Function, and Vascular Endothelial Function in Middle-aged Obese Women. *Korean Journal of Sport Science*. Published online March 31, 2023:1-12. doi:10.24985/kjss.2023.34.1.1

24. Kitzman DW, Brubaker PH, Herrington DM, et al. Effect of Endurance Exercise Training on Endothelial Function and Arterial Stiffness in Older Patients With Heart Failure and Preserved Ejection Fraction. *Journal of the American College of Cardiology*. 2013;62(7):584-592. doi:10.1016/j.jacc.2013.04.033

25. Kwon HR, Min KW, Ahn HJ, et al. Effects of Aerobic Exercise vs. Resistance Training on Endothelial Function in Women with Type 2 Diabetes Mellitus. *Diabetes Metab J*. 2011;35(4):364. doi:10.4093/dmj.2011.35.4.364

26. Lavrenčič A, Salobir BG, Keber I. Physical Training Improves Flow-Mediated Dilation in Patients With the Polymetabolic Syndrome. *ATVB*. 2000;20(2):551-555. doi:10.1161/01.ATV.20.2.551

27. Lee K, Kang I, Mack WJ, et al. Effects of high-intensity interval training on vascular endothelial function and vascular wall thickness in breast cancer patients receiving anthracycline-based chemotherapy: a randomized pilot study. *Breast Cancer Res Treat*. 2019;177(2):477-485. doi:10.1007/s10549-019-05332-7

28. Mohamed MAEN, Helmy ZM, Elrefaey BH, Abdusalam IH, Abd Elghany AM. Long term effect of physical therapy program in refractory anginal patients: A randomized controlled trial. Fizjoterapia Polska. 2021;21(5):190-194.

29. Molmen-Hansen HE, Stolen T, Tjonna AE, et al. Aerobic interval training reduces blood pressure and improves myocardial function in hypertensive patients. *Eur J Prev Cardiolog*. 2012;19(2):151-160. doi:10.1177/1741826711400512

30. Olson TP, Dengel DR, Leon AS, Schmitz KH. Moderate Resistance Training and Vascular Health in Overweight Women. *Medicine & Science in Sports & Exercise*. 2006;38(9):1558-1564. doi:10.1249/01.mss.0000227540.58916.0e

31. Orio F, Muscogiuri G, Giallauria F, et al. Oral contraceptives *versus* physical exercise on cardiovascular and metabolic risk factors in women with polycystic ovary syndrome: a randomized controlled trial. *Clinical Endocrinology*. 2016;85(5):764-771.

doi:10.1111/cen.13112

32. Pattyn N, Vanhees L, Cornelissen VA, et al. The long-term effects of a randomized trial comparing aerobic interval versus continuous training in coronary artery disease patients: 1-year data from the SAINTEX-CAD study. *Eur J Prev Cardiolog*. 2016;23(11):1154-1164. doi:10.1177/2047487316631200

33. Peng L, Chen L, Xia J, et al. The Effects of Time-Restricted Feeding and Exercise on Cardiometabolic Health in Sedentary Older Adults: A Randomized Controlled Trial. *Medicine & Science in Sports & Exercise*. 2026;58(7):1337-1351. doi:10.1249/MSS.0000000000003962

34. Pugh CJA, Sprung VS, Kemp GJ, et al. Exercise training reverses endothelial dysfunction in nonalcoholic fatty liver disease. *American Journal of Physiology-Heart and Circulatory Physiology*. 2014;307(9):H1298-H1306. doi:10.1152/ajpheart.00306.2014

35. Rahimianmashhad et al. 2020.

36. Robinson AT, Franklin NC, Norkeviciute E, et al. Improved arterial flow-mediated dilation after exertion involves hydrogen peroxide in overweight and obese adults following aerobic exercise training. *Journal of Hypertension*. 2016;34(7):1309-1316. doi:10.1097/HJH.0000000000000946

37. Sawyer BJ, Tucker WJ, Bhammar DM, Ryder JR, Sweazea KL, Gaesser GA. Effects of high-intensity interval training and moderate-intensity continuous training on endothelial function and cardiometabolic risk markers in obese adults. *J Appl Physiol*. 2016;121(1):279-288. doi:10.1152/japplphysiol.00024.2016

38. Oliveira e Silva VR, Stringuetta Belik F, Hueb JC, et al. Aerobic Exercise Training and Nontraditional Cardiovascular Risk Factors in Hemodialysis Patients: Results from a Prospective Randomized Trial. *Cardiorenal Med*. 2019;9(6):391-399. doi:10.1159/000501589

39. Desch S, Sonnabend M, Niebauer J, et al. Effects of physical exercise versus rosiglitazone on endothelial function in coronary artery disease patients with prediabetes. *Diabetes Obes Metab*. 2010;12(9):825-828. doi:10.1111/j.1463-1326.2010.01234.x

40. Turri-Silva N, Vale-Lira A, Verboven K, Quaglioti Durigan JL, Hansen D, Cipriano G. High-intensity interval training versus progressive high-intensity circuit resistance training on endothelial function and cardiorespiratory fitness in heart failure: A preliminary randomized controlled trial. Abdelbasset WK, ed. *PLoS ONE*. 2021;16(10):e0257607. doi:10.1371/journal.pone.0257607

41. Vaccarino V, Kondwani KA, Kelley ME, et al. Effect of Meditation on Endothelial Function in Black Americans With Metabolic Syndrome: A Randomized Trial. *Psychosomatic Medicine*. 2013;75(6):591-599. doi:10.1097/PSY.0b013e31829ac4f4

42. Valentino SE, Dunford EC, Dubberley J, et al. Cardiovascular responses to high-intensity stair

climbing in individuals with coronary artery disease. *Physiological Reports*. 2022;10(10):e15308. doi:10.14814/phy2.15308

43. Vinet A, Obert P, Courteix D, et al. Different modalities of exercise improve macrovascular function but not microvascular function in metabolic syndrome: The RESOLVE randomized trial. *International Journal of Cardiology*. 2018;267:165-170. doi:10.1016/j.ijcard.2018.05.073

44. Vona M, Rossi A, Capodaglio P, et al. Impact of physical training and detraining on endothelium-dependent vasodilation in patients with recent acute myocardial infarction. *American Heart Journal*. 2004;147(6):1039-1046. doi:10.1016/j.ahj.2003.12.023

45. Vona M, Codeluppi GM, Iannino T, Ferrari E, Bogousslavsky J, Von Segesser LK. Effects of Different Types of Exercise Training Followed by Detraining on Endothelium-Dependent Dilation in Patients With Recent Myocardial Infarction. *Circulation*. 2009;119(12):1601-1608. doi:10.1161/CIRCULATIONAHA.108.821736

46. Westhoff TH, Franke N, Schmidt S, et al. Beta-blockers do not impair the cardiovascular benefits of endurance training in hypertensives. *J Hum Hypertens*. 2007;21(6):486-493. doi:10.1038/sj.jhh.1002173

47. Yu Z, Tang S, Hu M, et al. Aquatic High-Intensity Interval Training Improves Vascular Function, Whereas Aquatic Moderate-Intensity Continuous Training Lowers Resting Heart Rate in Overweight and Obese Young Adults: A Randomized Controlled Trial. *jsportscimed*. Published online September 1, 2025:666-675. doi:10.52082/jssm.2025.666
